# CRISPR-Cas9^D10A^ nickase-based genotypic and phenotypic screening to enhance genome editing

**DOI:** 10.1038/srep24356

**Published:** 2016-04-15

**Authors:** Ting-Wei Will Chiang, Carlos le Sage, Delphine Larrieu, Mukerrem Demir, Stephen P. Jackson

**Affiliations:** 1 Wellcome Trust/Cancer Research UK Gurdon Institute, University of Cambridge, Cambridge CB2 1QN, UK; 2 Department of Biochemistry, University of Cambridge, Cambridge CB2 1GA, UK; 3 The Wellcome Trust Sanger Institute, Hinxton, Cambridge CB10 1SA, UK

## Abstract

The RNA-guided Cas9 nuclease is being widely employed to engineer the genomes of various cells and organisms. Despite the efficient mutagenesis induced by Cas9, off-target effects have raised concerns over the system’s specificity. Recently a “double-nicking” strategy using catalytic mutant Cas9^D10A^ nickase has been developed to minimise off-target effects. Here, we describe a Cas9^D10A^-based screening approach that combines an All-in-One Cas9^D10A^ nickase vector with fluorescence-activated cell sorting enrichment followed by high-throughput genotypic and phenotypic clonal screening strategies to generate isogenic knockouts and knock-ins highly efficiently, with minimal off-target effects. We validated this approach by targeting genes for the DNA-damage response (DDR) proteins MDC1, 53BP1, RIF1 and P53, plus the nuclear architecture proteins Lamin A/C, in three different human cell lines. We also efficiently obtained biallelic knock-in clones, using single-stranded oligodeoxynucleotides as homologous templates, for insertion of an *EcoRI* recognition site at the *RIF1* locus and introduction of a point mutation at the histone *H2AFX* locus to abolish assembly of DDR factors at sites of DNA double-strand breaks. This versatile screening approach should facilitate research aimed at defining gene functions, modelling of cancers and other diseases underpinned by genetic factors, and exploring new therapeutic opportunities.

The clustered, regularly interspaced, short palindromic repeats (CRISPR)-Cas (CRISPR-associated) systems function in the adaptive immunity of bacteria and archaea to attack invading foreign genetic elements^1^,^2^,^3^,^4^. Recently, the *Streptococcus pyogenes* type II CRISPR-Cas9 system has been adapted to perform genome engineering by inducing DNA double-strand breaks (DSBs) that can be repaired by either non-homologous end-joining (NHEJ) or homology-directed repair (HDR)^5^,^6^,^7^,^8^. Mutagenic NHEJ can induce insertions or deletions (indels) at repair sites that may cause frame-shifts in open reading frames, yielding truncated proteins and premature stop codons in the mRNA, a key inducer of nonsense-mediated mRNA decay. Alternatively, HDR allows precise knock-in of genetic modifications, such as point mutations, insertions and epitope tagging. The CRISPR-Cas9 system can efficiently generate genome modifications, simply relying on the presence of a proto-spacer adjacent motif (PAM) and a twenty-nucleotide small guide RNA (sgRNA) complementary to the target DNA by Watson-Crick base pairing^9^. While an sgRNA can usually be designed to have high specificity for the desired target site, in large genomes such as those of mammalian cells, there are often related sequences, which contain one or more mismatches with the sgRNA that may be tolerated by Cas9, thereby representing potential undesirable off-target sites. Indeed, there is growing evidence for such off-target effects^10^,^11^,^12^,^13^,^14^,^15^,^16^,^17^,^18^,^19^,^20^,^21^,^22^ that may confound experimental results and limit utility of the CRISPR-Cas9 system, particularly in clinical settings.

Recent findings have indicated that CRISPR-Cas9 off-target effects can be reduced by various methods including the use of shortened sgRNAs^23^, FokI-Cas9 fusion nucleases^24^,^25^, purified Cas9 ribo-nucleoproteins^26^, rationally-engineered Cas9^27^, modified sgRNAs^10^,^16^, or paired catalytic mutant Cas9 nickases^5^,^28^,^29^. However, several of these methods often achieve lower off-target effects at the expense of reducing on-target efficiency.

Significant attention has focused on Cas9 nickases (RuvC^D10A^ or HNH^H840A^) which, unlike wild-type Cas9 that generates blunt DSBs, cut only one strand of the DNA, generate single-strand breaks (SSB) that can be repaired faithfully, without inducing indels. In order to create DSBs, a double-nicking strategy that involves paired nickases targeting adjacent regions has recently been developed, meaning that the potential for off-target DSBs is very much minimised. However, co-transfection of multiple plasmids, including a Cas9 nickase, two sgRNAs and a fluorescent marker (or a drug selection homology vector) may compromise transfection efficiency and targeting mutagenesis, which has so far discouraged the widespread use of this approach. Here, we establish an approach based on an All-in-One plasmid encoding dual sgRNAs and fluorescent protein-coupled Cas9^D10A^ nickase that circumvents these issues and thereby allows efficient genome engineering with minimal off-target effects.

## Results

### Enhanced mutagenic targeting with minimised off-target effects via an All-in-One Cas9^D10A^ nickase vector

**Figure 1:**
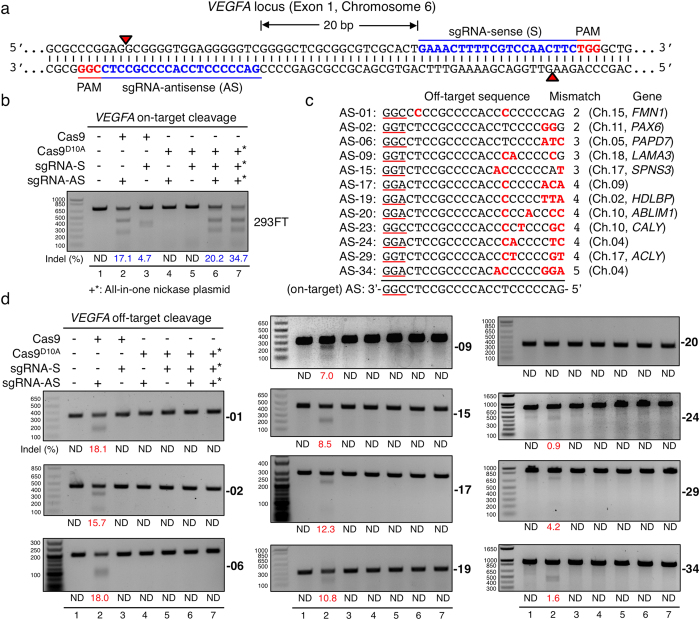
On- and off-target mutagenic efficiencies of sgRNAs targeting *VEGFA* in human cells. (**a**) Double nicking strategy with sense (S) and antisense (AS) sgRNAs separated by 20 base pairs at exon 1 of the *VEGFA* locus on chromosome 6. Red arrows indicate the nicking sites of Cas9^D10A^ nickase, which generates 5′ overhangs at target sites. (**b**) On-target mutagenic efficiencies of *VEGFA* sgRNAs by T7 EI assays in human HEK293FT cells. Cells were either transfected with or without (lane 1) wild-type Cas9 vector containing either the AS (lane 2) or the S sgRNA (lane 3), single sgRNA Cas9^D10A^ nickase vector carrying either the AS (lane 4) or the S sgRNA (lane 5), two single sgRNA nickase vectors each containing Cas9^D10A^ nickase with either the S or the AS sgRNA (lane 6), or dual sgRNA All-in-One Cas9^D10A^ nickase vector (lane 7). Indel frequencies are shown below. ND, not detected. (**c**) Twelve off-target sites of the AS sgRNA were assessed. Mismatch nucleotides are indicated in red, and PAM motifs (NGG) are underlined. The number of mismatches, associated genes and chromosomes are indicated. (**d**) Off-target cleavages by wild-type Cas9 with the AS sgRNA at eleven off-target sites assessed. Note that we were unable to get conclusive results at AS-23 site where PCR products, amplified by two different sets of primers, were unexpectedly degraded by T7 EI across all samples, which may arise from high heterogeneity at this particular genomic locus. +*All-in-One nickase plasmid.

We designed an All-in-One plasmid vector that contains dual U6 promoter-driven sgRNA cassettes and encodes Cas9^D10A^ nickase, coupled via a “ribosomal-skipping” 2A peptide-linker^30^, to the fluorescent marker protein EGFP (enhanced green-fluorescent protein)^5^,^31^ or mCherry (in this study, T2A linked with EGFP, P2A with mCherry). To assess on- and off-target mutagenic efficiencies, we first chose *VEGFA* target sites^19^ to compare our dual-sgRNA All-in-One Cas9^D10A^ nickase vector with a wild-type Cas9 vector and a single sgRNA Cas9^D10A^ nickase vector. A pair of sense (S) and antisense (AS) sgRNAs was designed for the double-nicking strategy (Fig. 1a). Human embryonic kidney (HEK293FT) cells were transfected and, three days later, harvested for T7 endonuclease I assays^32^ to identify targeting efficiencies. As shown in Fig. 1b, cells transfected with the dual sgRNA All-in-One Cas9^D10A^ nickase vector displayed the highest on-target mutagenesis (34.7%, lane 7), which was seven-fold and two-fold higher than that obtained with wild-type Cas9 together with the sense (4.7%, lane 3) or antisense (17.1%, lane 2) sgRNAs, respectively. In addition, the All-in-One Cas9^D10A^ nickase vector was 1.7-fold more effective compared to co-transfecting two single sgRNA Cas9^D10A^ nickase vectors each carrying either the S or the AS sgRNA (lane 6). To investigate whether off-target effects can be reduced in our vector system, we assessed previously established twelve off-target sites of the AS sgRNA with two to five mismatches (Fig. 1c)^19^. We found significant off-target mutagenesis caused by wild-type Cas9 at ten off-target sites (Fig. 1d, lanes 2), which in some cases were comparable to on-target mutagenic efficiency (see AS-01, -02 and -06). Strikingly, despite the All-in-One Cas9^D10A^ nickase vector displaying the highest on-target mutagenic efficiency, we were unable to detect any off-target cleavage at those sites tested (Fig. 1d, lanes 7). In accord with previous studies^28^,^29^, we did not detect any on- or off-target mutagenesis by inducing SSBs (Fig. 1b,d, lanes 4 and 5).

**Figure 2:**
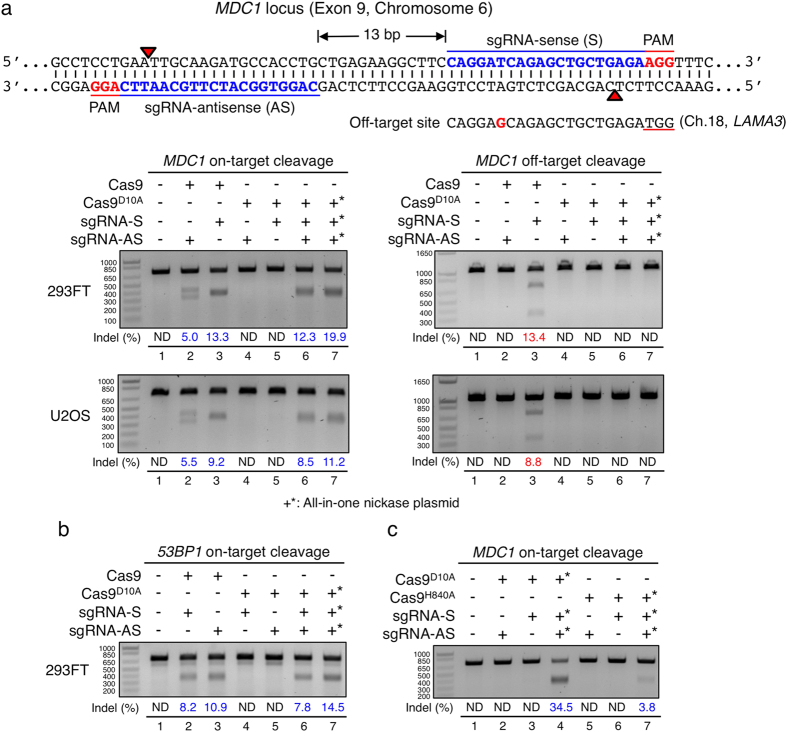
On- and off-target mutagenic efficiencies of sgRNAs targeting *MDC1* or *53BP1*. **(a)** Double nicking strategy with sense (S) and antisense (AS) sgRNAs separated by 13 base pairs at exon 9 of the *MDC1* locus on chromosome 6. One verified off-target site of the S sgRNA (located on chromosome 18, *LAMA3*) is shown below with one mismatch indicated in red. The on- and off-target mutagenic efficiencies were assessed by T7 EI assays in human HEK293FT and U2OS cells. Cells were either transfected with or without (lane 1) wild-type Cas9 vector carrying either the AS (lane 2) or the S sgRNA (lane 3), single-sgRNA Cas9^D10A^ nickase vector containing either the AS (lane 4) or the S sgRNA (lane 5), two single sgRNA nickase vectors each carrying Cas9^D10A^ nickase with either the S or the AS sgRNA (lane 6), or dual sgRNA All-in-One Cas9^D10A^ nickase vector (lane 7). **(b)** On-target mutagenic efficiencies of sgRNAs targeting the exon 10 of *53BP1* locus. **(c)** On-target mutagenic efficiencies by Cas9^D10A^ or Cas9^H840A^ nickases at the *MDC1* locus. Cells were either transfected with or without (lane 1) single sgRNA Cas9^D10A^ nickase vector containing either the AS (lane 2) or the S sgRNA (lane 3), All-in-One Cas9^D10A^ nickase vector (lane 4), single sgRNA Cas9^H840A^ nickase vector carrying either the AS sgRNA (lane 5) or the S sgRNA (lane 6), or All-in-One Cas9^H840A^ nickase vector (lane 7). +*All-in-One nickase plasmid.

To expand our analyses, we decided to target the genes encoding the DNA-damage response (DDR) proteins MDC1 or 53BP1. As for our analysis of *VEGFA*, we determined targeting efficiencies by comparing different vector systems. In line with our previous findings, the All-in-One nickase vector system produced highest on-target mutagenic efficiencies at the *MDC1* locus in both HEK293FT cells and U2OS cells (Fig. 2a left panels, lanes 7). We also discovered an off-target site of the sense sgRNA used to target *MDC1*, which possessed a mismatch at the sixth nucleotide from the 5′ end (Fig. 2a). Notably, when combined with wild-type Cas9, the efficiency of mutagenesis at this off-target site was comparable to on-target efficiency in both cell lines tested (Fig. 2a right panels, lanes 3). By contrast, we did not detect any off-target cleavage with the All-in-One nickase vector system (Fig. 2a, right panels, lanes 7), demonstrating its high specificity. We also performed T7 EI assays on cells targeted for the *53BP1* locus and found the same trend that the All-in-One nickase vector system was the most efficient at inducing indels (Fig. 2b).

Intriguingly, when we compared the mutagenic targeting efficiency of Cas9^D10A^ nickase with that of Cas9^H840A^ nickase in the All-in-One vector system at the *MDC1* locus, Cas9^D10A^ nickase produced nine-fold higher levels of mutagenesis than Cas9^H840A^ (Fig. 2c and Supplementary Fig. 1 for the *53BP1* and *RIF1* loci). This suggested that different overhangs (5′ overhangs created by Cas9^D10A^ and 3′ overhangs by Cas9^H840A^) may influence the efficiency of mutagenesis during DNA end-joining repair. In sum, we conclude that the All-in-One vector system enables efficient gene targeting, particularly when used in the context of Cas9^D10A^ nickase, and that it does so with minimised off-target effects. As discussed further below, we speculate that such a system will be particularly beneficial in targeting relatively hard-to-transfect cell lines, since all the essential elements are assembled in one single vector for each target locus.

### High-throughput PCR-based genotypic and microscopy-based phenotypic screens for gene knockout clones

**Figure 3:**
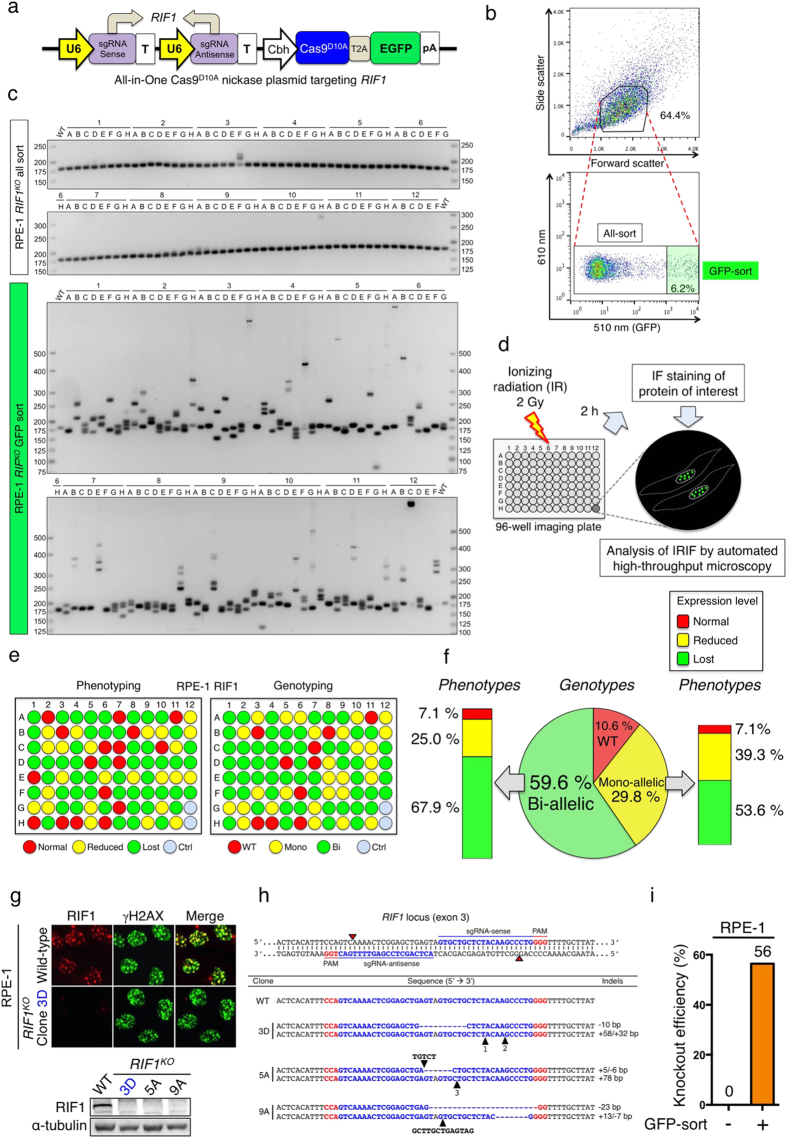
Genotypic and phenotypic screens for *RIF1* knockout clones in RPE-1 cells. (**a**) Schematic of the All-in-One Cas9^D10A^ nickase vector targeting *RIF1*. (**b**) FACS data showing the sequential gating of healthy (all-sort, 64.4%, top panel) and high-EGFP expressing (GFP-sort, 6.2%, bottom panel) cell populations. Each of these populations was sorted separately into 96-well plates at the level of a single cell per well. **(c)** PCR-genotyping of 94 clones from all- and GFP-sorted populations to identify indels at the target site. Wild-type (WT) product size is 182 bp. **(d)** Schematic of the automated high-throughput microscopy-based phenotypic screen for RIF1 knockout clones. Cells were subjected to 2 Gy of IR. After 2 h, immunofluorescent cell staining was carried out for RIF1 protein. IR-induced foci were then analysed by high-throughput automatic microscopy. **(e,f)** Genotypic and phenotypic screening outputs of RIF1 knockout clones were compared in parallel. In genotyping panel: WT, clones with no apparent indel formation. Mono, clones with monoallelic indel mutations. Bi, clones with biallelic indel mutations. Ctrl, wild-type non-targeted clones. In phenotyping panel: Normal, normal foci equivalent to wild-type. Reduced, reduced foci compared to wild-type. Lost, clones with no staining of foci. **(g)** Representative IF images of RPE-1 *RIF1* knockout and wild-type cells from high-throughput microscopy screening. Cells were co-stained with RIF1 and γH2AX antibodies. Level of RIF1 protein was verified by Western blotting, shown below. **(h)** DNA sequencing of *RIF1* knockout clones at both alleles. Red arrowheads indicate the Cas9^D10A^ nicking sites. Each sequence represents one allele. Dashed lines indicate deletions; black arrowheads indicate the precise locations of insertions. Inserted sequences are either tagged with black arrowheads, or numbered and shown in Supplementary Fig. 3. **(i)** Phenotypic RIF1 knockout efficiencies of all-sort and GFP-sort clones in RPE-1 cells.

To further assess the utility of the All-in-One Cas9^D10A^ nickase vector system, we explored how it could be employed for genome editing in telomerase-immortalized, non-transformed human diploid retinal pigment epithelial (RPE-1) cells. We also examined how genotypic and phenotypic screens could be used to facilitate the identification of intended clones (an illustrative step-wise screening method is shown in Supplementary Fig. 2). Cells were transfected with an All-in-One Cas9^D10A^ nickase vector targeting the gene for the DDR protein RIF1 (Fig. 3a) and, after two days, flow-activated cell sorting (FACS) was carried out (Fig. 3b) to collect all healthy cells (“all-sort”, corresponding to 64.4% of the total cell population) or high EGFP-expressing cells (“GFP-sort”, 6.2% of the total cell population) into 96-well plates at the level of a single cell per well. As the 2A peptide linker allows for stoichiometric and concordant expression of the EGFP and Cas9^D10A^ nickase^30^, these data thus indicated that only a relatively small proportion of the total transfected RPE-1 cell population was likely to express Cas9^D10A^ nickase (as expected because RPE-1 cells are relatively difficult to efficiently transfect). After assembly and expansion of viable clones into new 96-well plates, we performed PCR-based genotypic screening across the *RIF1* target locus to assess the mutational status of each cell clone. As shown in Fig. 3c, few mutagenized clones (2%) were identified from the non-EGFP-enriched population (top panel). In marked contrast, for clones derived via EGFP-enrichment, a large proportion (~90%) displayed indels at the target site (bottom panel). These results thus showed that FACS-based sorting can markedly increase mutagenic gene-targeting efficiency.

Many DDR proteins function to elicit DNA-damage checkpoint responses, promote DNA repair and/or control DNA-repair pathway choice^33^,^34^. Furthermore, various DDR proteins are recruited to DNA-damage sites, often forming sub-nuclear structures, termed foci, that assemble in highly orchestrated ways^35^. For instance, ionizing radiation induced foci (IRIF) formed by 53BP1 depend on prior recruitment of MDC1; and in turn, 53BP1 is needed for IRIF formation by RIF1. We therefore speculated that IRIF assembly could be employed as a phenotypic readout for screening when targeting the genes encoding such DDR components. To assess this potential, we treated the clones derived via EGFP-enrichment (the same clones used in the genotypic screening in Fig. 3c, bottom panel) with 2 Gy of IR, and two hours later, subjected them to immunofluorescent staining for RIF1 protein followed by high-throughput confocal microscopy-based screening (depicted in Fig. 3d). Clones were then scored on the basis of their ensuing staining patterns being essentially equivalent to those of control, non-targeted cells (Normal); reduced compared to control cells (Reduced), or displaying no clear IRIF staining for RIF1 (Lost). Figure 3e,f present these screening outputs in comparison to parallel genotyping data that predicted biallelic mutations (indel formation) in 59.6% of the clones, monoallelic mutations in 29.8% of the clones, and no apparent indel formation in the remaining 10.6% of clones. Notably, there was generally good concordance between the two datasets, with 67.9% of the clones with clear biallelic mutations turning out to lack detectable IRIF for RIF1 (Fig. 3f; presumably the others contained indels in one or both alleles that did not change the *RIF1* reading frame nor substantially affect ensuing protein function and stability). Thus, in cases when phenotypic screening is not feasible, genotypic screening for clones with biallelic mutations will often identify intended knockout clones. We also observed that 25% of predicted biallelically mutated clones showed reduced RIF1 foci, suggesting that one allele likely possessed an in-frame mutation. Moreover, 7.1% remained phenotypically wild-type, implying that mutations at both alleles did not affect RIF1 protein function. Furthermore, 53.6% of clones predicted to be only monoallelically mutated turned out to be phenotypically knockout in terms of RIF1 IRIF formation. This likely reflected the agarose-gel electrophoresis not being of sufficient resolution to identify PCR products containing very small indels compared to wild-type. Nonetheless, 39.3% of clones scored by analysis of PCR products as being monoallelically targeted displayed detectable but reduced RIF1 foci, suggesting that they were indeed heterozygous for *RIF1* mutation. In some circumstances where heterozygous clones are desired, genotypic screening for monoallelic-targeted clones should thus be prioritised. As expected, all the clones showing a wild-type band on genotypic screening were scored as having a normal phenotype at the protein level. In conclusion, these studies established that, while genotypic screening alone can identify functional knockout clones, combining both genotypic and phenotypic screening is most effective.

Further analyses showed that wild-type cells exhibited co-localised IRIF of RIF1 and Ser139-phosphorylated H2AX (γH2AX), a marker for DSB sites^36^, while RIF1 knockout cells showed no RIF1 IRIF but normal γH2AX foci (Fig. 3g). When we analysed such RIF1 knockout clones further, we found that they indeed lacked RIF1 expression as measured by Western immunoblotting (Fig. 3g, bottom panel), and possessed biallelic mutations as revealed by DNA sequencing (Fig. 3h and Supplementary Fig. 3). Crucially, when we compared the phenotypic knockout efficiencies of all-sort and GFP-sort clones, we observed that these were 0% and 56%, respectively (Fig. 3i). These phenotyping data therefore corresponded well with the genotyping results (Fig. 3c) and further highlighted how fluorescent protein-expression sorting can dramatically enhance the recovery of cell clones disrupted for target-gene function.

**Figure 4:**
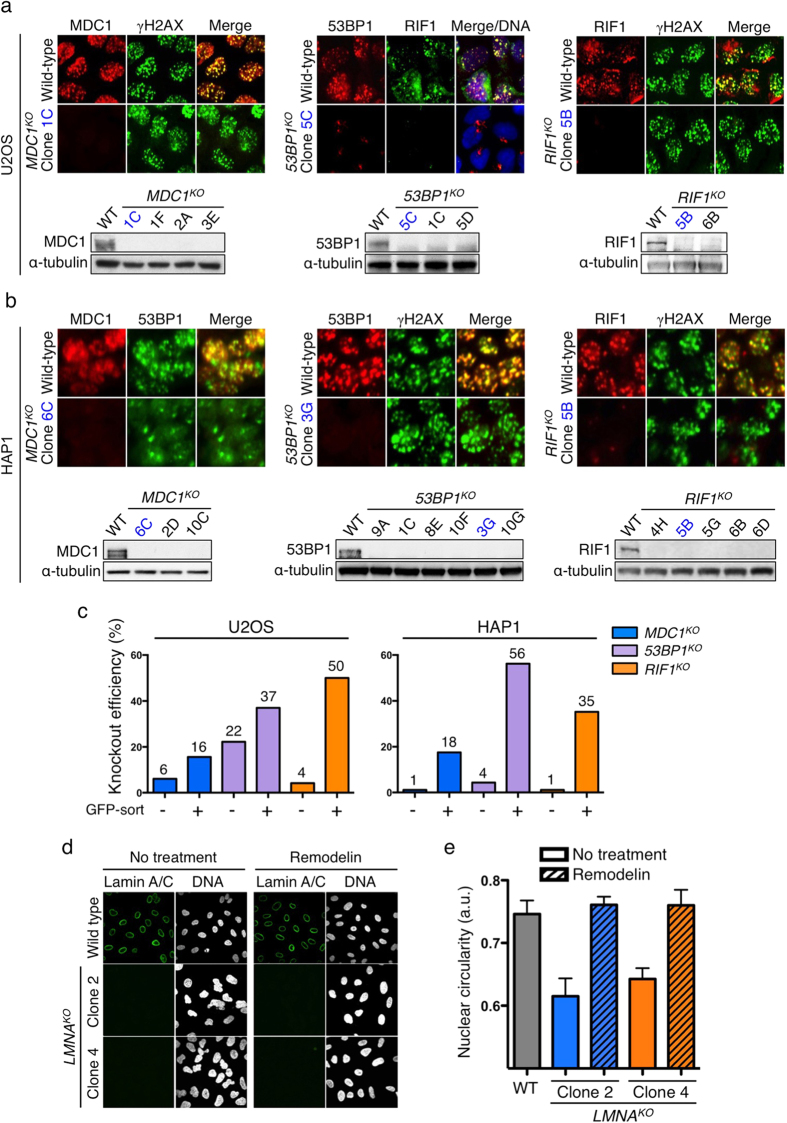
High-throughput microscopy-based phenotypic screening for knockout clones in human U2OS and HAP1 cells. (**a**) Representative IF images of knockout clones for MDC1, 53BP1 and RIF1 in U2OS cells from the automated high-throughput microscopy screen. In the middle panel, merge/DNA indicates the overlay of 53BP1, RIF1 and DAPI (DNA) staining. Western blots are shown below. (**b**) Representative IF images of knockout clones for MDC1, 53BP1 and RIF1 in HAP1 cells. (**c**) Phenotypic knockout efficiencies of all-sort and GFP-sort clones in U2OS and HAP1 cells. (**d**) Confocal microscopy images of *LMNA* knockouts in U2OS cells with or without Remodelin treatment. Cells were co-stained with Lamin A/C antibody and DAPI (DNA). (**e**) Quantification of nuclear circularities of wild-type and *LMNA* knockout cells in the presence or absence of Remodelin.

To test the applicability of the All-in-One Cas9^D10A^ nickase vector system and screening approaches in other cell lines, we targeted the DDR genes *MDC1*, *53BP1* and *RIF1* in human aneuploid osteosarcoma (U2OS) cells and haploid chronic myelogenous leukaemia derived (HAP1) cells. As shown in Fig. 4a,b, the combination of genotypic and phenotypic screening allowed us to effectively identify clones lacking target-protein expression as assessed by high-throughput immunofluorescence microscopy and Western immunoblotting, with ensuing DNA sequencing verifying the biallelic mutational status of selected clones (Supplementary Figs 4c and 8c). In accord with published findings^35^, we did not observe RIF1 foci in *53BP1* knockout U2OS cells (Fig. 4a, middle panel). In addition, since the 53BP1 foci formation requires MDC1, in HAP1 cells disrupted for MDC1 function, we did not observe 53BP1 foci and 53BP1 staining remained pan-nuclear (Fig. 4b, left panel).

Through genotypic screening, we found that 88% and 38% of U2OS clones arising from EGFP-enriched and non-enriched cell populations, respectively, bore readily apparent mutations in *MDC1* (Supplementary Fig. 4). Moreover, through phenotypic screening, 16% and 6% of EGFP-enriched and non-enriched clones, respectively, were scored as being completely negative for MDC1 expression (Fig. 4c). Similarly, for *53BP1*-targeting of U2OS cells, 94% and 42% of clones from EGFP-enriched and non-enriched populations, respectively, were scored as being mutated with various indels (Supplementary Fig. 5), with phenotypic screening identifying 37% and 22% of EGFP-enriched and non-enriched clones as entirely lacking 53BP1 expression (Fig. 4c). The most striking effect of EGFP sorting of U2OS cells, however, was observed when we targeted the *RIF1* gene. Here, 77% and 8% of clones arising from EGFP-enriched and non-enriched populations, respectively, displayed mutagenized alleles (Supplementary Fig. 6), and 50% and 4% of EGFP-enriched and non-enriched clones, respectively, were found to lack RIF1 expression (Fig. 4c). The results show that even though U2OS is a readily transfectable cell line (see FACS profiles in Supplementary Figs 4–6), the EGFP-enrichment step on average led to 4.6-fold and 5.6-fold enhancements of mutagenic efficiencies as measured by genotypic and phenotypic screening, respectively.

Having focused on readily transfectable U2OS cells, we next carried out studies to knock out the genes for MDC1, 53BP1 and RIF1 in relatively difficult-to-transfect human HAP1 cells (see FACS profiles in Supplementary Figs 7–9). Ensuing genotypic-screening analyses revealed that only low levels of gene targeting were achieved without sorting for EGFP expression. Indeed, for the genes we targeted, the proportion of mutagenized clones was increased, on average by over 20-fold via the use of an EGFP-sorting step (Supplementary Figs 7–9). Moreover, phenotypic-screening studies established that EGFP sorting enhanced the recovery of clones lacking detectable MDC1, 53BP1 or RIF1 expression over 22-fold on average (Fig. 4b,c). These findings further underlined our previous conclusions. Furthermore, they highlighted how combining the All-in-One Cas9^D10A^ nickase vector system with a FACS-enrichment step followed by high-throughput genotypic and/or phenotypic screening can be particularly beneficial for studies with difficult-to-transfect cell lines such as HAP1.

To broaden the scope of phenotypic screening, we investigated nuclear morphological changes as a readout for functional disruption of *LMNA*, which encodes for nuclear-architecture proteins Lamin A and C. *LMNA* disruption or mutations lead to misshapen nuclei and altered chromatin organization associated with cancer and laminopathies^37^,^38^. Thus, we used the All-in-One Cas9^D10A^ nickase vector system to target *LMNA* in U2OS cells and performed screens to identify knockout clones. As shown in Fig. 4d, unlike wild-type cells, *LMNA* knockout cells completely lost Lamin A/C nuclear envelope staining. Furthermore, supporting previous work involving siRNA-mediated Lamin A/C depletion^39^, *LMNA* knockout cells exhibited misshapen nuclei as seen by DNA staining (Fig. 4d, left panel), a phenotype that was rescued by treating cells with the small-molecule compound Remodelin^39^ (Fig. 4d, right panel and quantified in Fig. 4e). We further verified two knockout clones by Western immunoblotting and DNA sequencing (Supplementary Fig. 10).

### Efficient clonal screening for double gene knockouts

**Figure 5:**
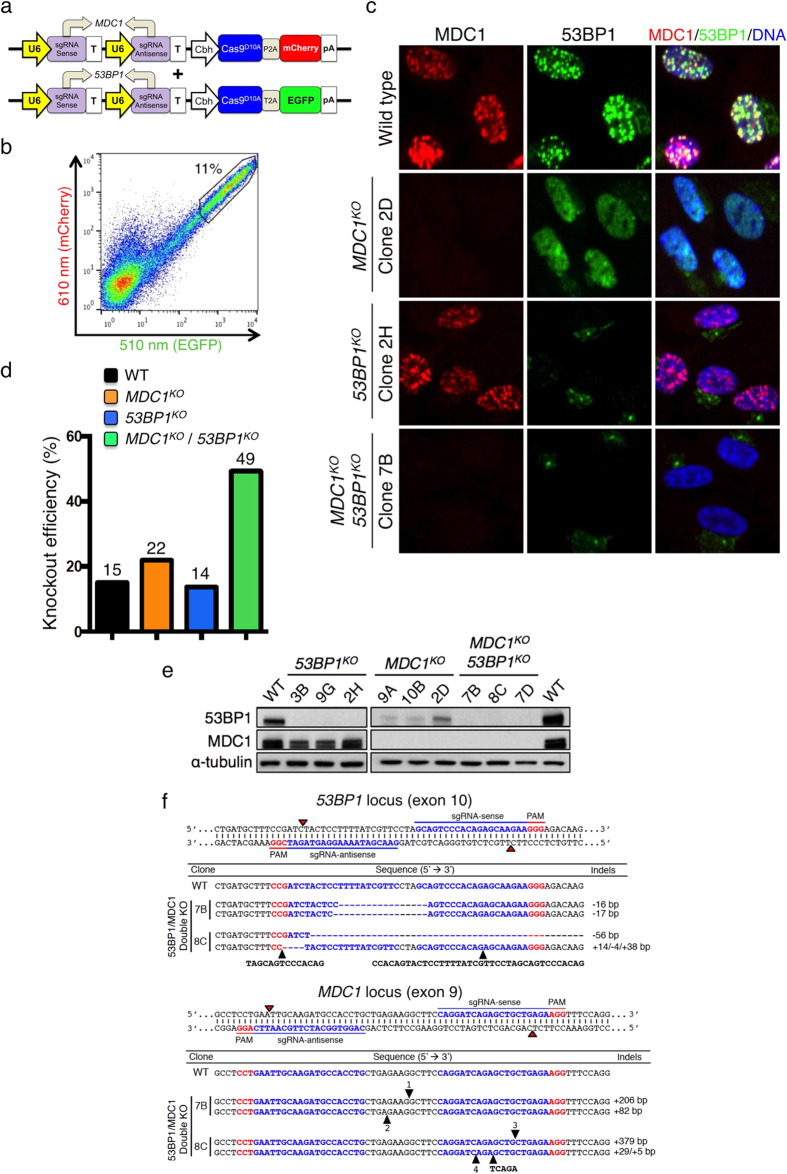
Simultaneous double knockout of *MDC1 and 53BP1*. (**a**) Sense and antisense sgRNAs targeting *MDC1* and *53BP1* were cloned into 2A-linked mCherry and EGFP All-in-One Cas9^D10A^ nickase vectors, respectively. (**b**) FACS single-cell sorting of cell populations co-expressing mCherry and EGFP gated for the top 11%. (**c**) Representative high-throughput microscopy IF images of wild-type, MDC1 knockout, 53BP1 knockout, and MDC1 and 53BP1 double knockout (*MDC1*^*KO*^*/53BP1*^*KO*^) RPE-1 cells. 53BP1/MDC1/DNA indicates the overlay of 53BP1, MDC1 and DAPI (DNA) staining. (**d**) Phenotypic knockout efficiencies of respective knockout populations. (**e**) Western immunoblots of respective knockout clones. (**f**) DNA sequencing of two *MDC1*^*KO*^*/53BP1*^*KO*^ clones across the *MDC1* and *53BP1* targeting loci. The full sequences of numbered insertions are shown in Supplementary Fig. 11f.

Multiplex gene targeting can be useful to study genetic interactions such as epistasis, synthetic lethality or genetic suppression. To determine the efficacy of our system in multiple gene targeting, we attempted to knock out *MDC1* and *53BP1* simultaneously in RPE-1 cells. Thus, we cloned sgRNAs targeting *MDC1* or *53BP1* into All-in-One Cas9^D10A^ nickase vectors containing a 2A peptide-linked mCherry or EGFP marker, respectively (Fig. 5a). Cells were then co-transfected with these two vectors and a cell population co-expressing mCherry plus EGFP was collected by FACS at the level of a single cell per well (Fig. 5b and Supplementary Fig. 11a). Strikingly, in genotypic screening, 97% and 96% of clones were found to be mutagenized with various indels at the *MDC1* and *53BP1* target loci, respectively; and furthermore, 95% were mutagenized at both target genes (Supplementary Fig. 11). As shown in Fig. 5c and summarised in Fig. 5d, phenotypic screenings established that a high proportion (49%) of clones lacked both MDC1 and 53BP1 IRIF (MDC1/53BP1 double-knockout), 22% lacked MDC1 foci but 53BP1 staining remained pan-nuclear (MDC1 knockout), 14% lacked 53BP1 staining but still formed MDC1 IRIF (53BP1 knockout), and 15% remained phenotypically functional for both proteins (wild-type). By examining selected clones, we confirmed their knockout status by Western immunoblotting (Fig. 5e) and DNA sequencing (Fig. 5f and Supplementary Fig. 11f). These results thereby demonstrated that employing the All-in-One Cas9^D10A^ nickase vector system with a multi-channel FACS-enrichment step followed by high-throughput genotypic and/or phenotypic screening methods can achieve highly efficient multiplex gene targeting.

### Drug-based functional screen for gene knockouts

Next we tested our screening method in the context of a drug-based selection strategy in which a cellular response, such as drug sensitivity or resistance can be used as a phenotypic readout. For this, we decided to target the tumour suppressor gene *TP53* using the All-in-One Cas9^D10A^ nickase vector system because, in the absence of functional p53 protein, cells exhibit insensitivity to Nutlin-3 treatment. Nutlin-3 has been reported to inhibit the interaction between MDM2 and p53, leading to p53-dependent cell cycle arrest^40^.

**Figure 6:**
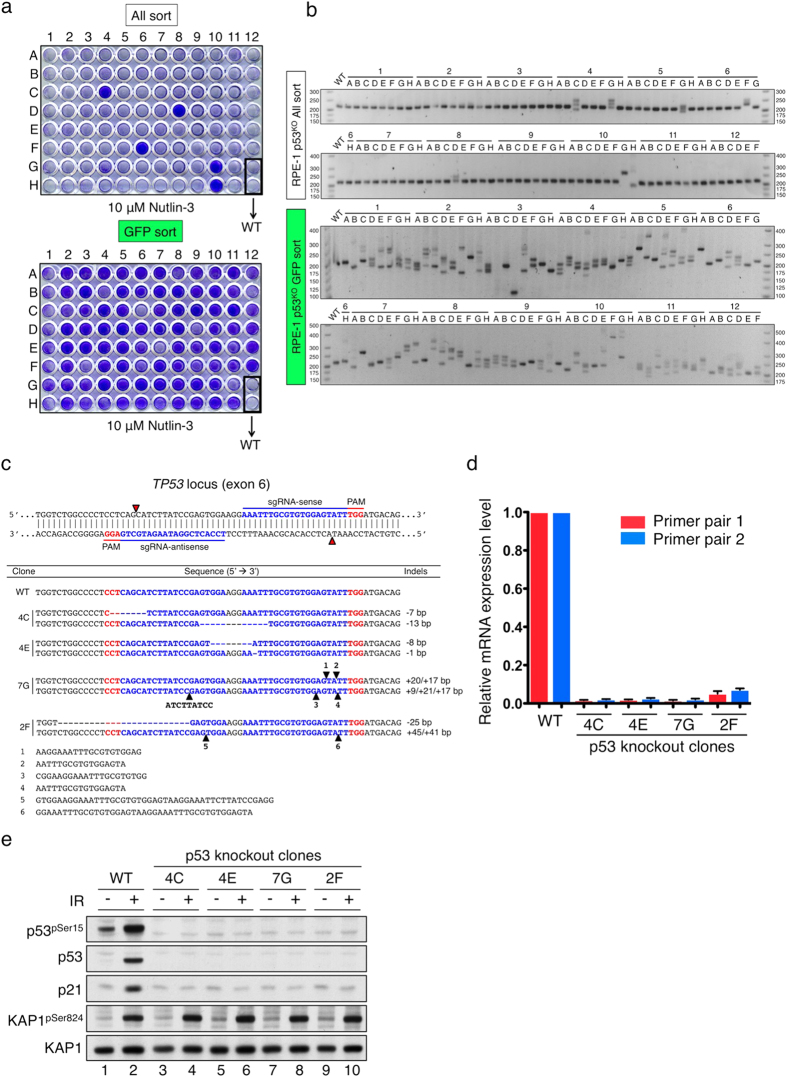
Drug-based (Nutlin-3) functional screen for *TP53* knockouts. (**a**) Nutlin-3 drug screen to identify p53 knockouts from all- and GFP-sort clones that were plated at a similar density and treated with 10 μM Nutlin-3 for five days prior to fixation and crystal violet staining. (**b**) PCR-genotyping of 94 clones from all- and GFP-sort populations to identify indels at the *TP53* target site. Wild-type (WT) product size is 221 bp. (**c**) DNA sequencing of *TP53* knockout clones, confirming biallelic disruption. (**d**) qRT-PCR of *TP53* mRNA levels in wild-type and knockout clones. (**e**) Functional validation of four *TP53* knockout clones with regard to DDR signalling. Cells were damaged with or without 10 Gy of IR. 8 h later, cells were harvested and analysed by Western immunoblotting. KAP1^pS824^ is a marker of DNA damage.

Firstly, we established that 5.3% of non-EGFP-enriched clones and 90.4% of clones arising from EGFP-sorting showed conspicuous resistance to Nutlin-3 in the phenotypic screen (Fig. 6a), suggesting that EGFP sorting enhanced *TP53* disruption 17-fold. In genotypic screening, we found that only 7.4% of non-EGFP-enriched clones (all-sort, Fig. 6b, top panel) displayed mutagenized alleles, whereas 94.7% of EGFP-enriched clones underwent substantial indel mutations at the *TP53* target site (bottom panel). Collectively, the majority (>90%) of mutagenized clones identified by genotypic screening exhibited Nutlin-3 resistance, revealing strong correlations between the genotypic and phenotypic screens. We further analysed several *TP53* knockout clones by DNA sequencing to confirm their biallelic mutational status (Fig. 6c). In line with this, qRT-PCR analyses established that such clones displayed a pronounced reduction (>98%) in *TP53* mRNA levels compared to wild-type cells (Fig. 6d), a phenomenon presumably reflecting nonsense-mediated mRNA decay of *TP53* transcripts bearing premature translational termination codons. To interrogate p53 function in the context of DNA damage, we treated p53 knockout cells with 10 Gy of IR. As shown in Fig. 6e, whereas p53 and phosphorylated p53 (p53^pSer15^) were induced upon DNA damage in p53 wild-type cells (lane 2), neither was observed in *TP53* knockout clones (lanes 3–10). Moreover, p53-dependent up-regulation of p21 (CIP1) was also lost in the *TP53* knockouts, indicating the effective disruption of p53-dependent DDR signalling which accounts for the insensitivity to Nutlin-3 treatment.

### Efficient biallelic knock-ins via Cas9^D10A^ nickase-based screening system

**Figure 7:**
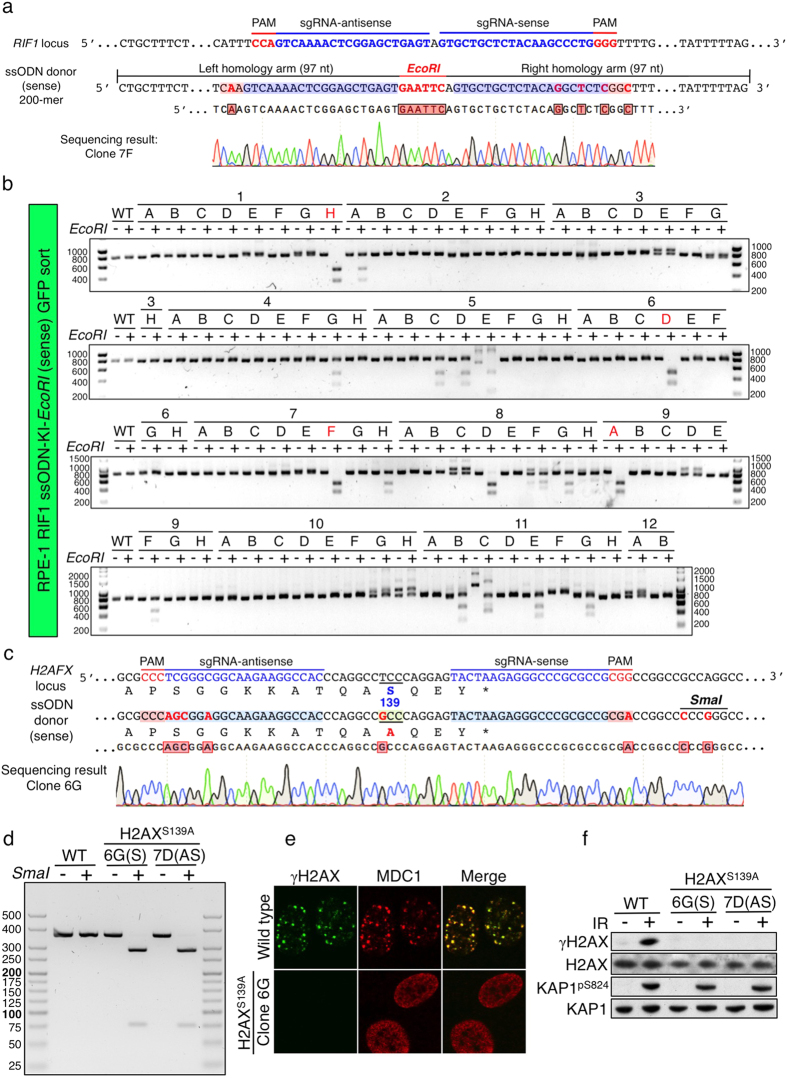
Precise biallelic knock-ins via HDR by Cas9^D10A^ nickase-based system. (**a**) Strategic knock-in insertion of an *EcoRI* site at exon 3 of the *RIF1* locus. Only the coding strand is shown. The intended mutations in the sense ssODN template are indicated in red. DNA sequencing of a homozygous knock-in clone 7F is aligned below. (**b**) Genotyping analysis of the PCR products across the knock-in site by *EcoRI* digestion. Clones indicated in red were verified by DNA sequencing as biallelic knock-ins where *EcoRI*-digested products were 487 bp and 310 bp. (**c**) H2AX^S139A^ point mutation knock-in strategy. Only the coding strand is shown and the corresponding amino acid sequence is aligned below. In the ssODN template, eight nucleotide mutations, including creation of a *SmaI* site, are indicated in red. DNA sequencing for homozygous knock-in clone 6G is aligned at the bottom. Successful knock-in nucleotides are indicated in red boxes. (**d**) Genotyping analysis of the PCR products across the *H2AFX* target site by *SmaI* digestion. Undigested product length is 363 bp and *SmaI*-digested products were 287 bp and 76 bp. (**e**) IRIF of wild-type and H2AX^S139A^ cells. Cells were subjected to 2 Gy of IR and fixed after 2 h, followed by γH2AX and MDC1 immunostaining. (**f**) Western immunoblots of wild-type and H2AX^S139A^ clones subjected to 10 Gy of IR and harvested after 20 min. KAP1^pS824^ is a marker of DNA damage.

The ability to precisely knock in site-specific mutations is of great value for researchers endeavouring to understand gene functions as well as to model disease-causing mutations. We therefore examined the All-in-One Cas9^D10A^ nickase system for its potential to induce site-specific knock-in mutations via HDR-mediated repair using single-stranded oligodeoxynucleotides (ssODN) as homologous templates. To test the system’s efficiency, we first attempted to insert an *EcoRI* recognition site (GAATTC) between the sense and antisense sgRNAs that we previously used to target *RIF1* (Fig. 7a). Thus, RPE-1 cells were co-transfected with the *RIF1*-targeted All-in-One Cas9^D10A^ nickase vector and either sense or antisense ssODN (200-nucleotide) donor containing an *EcoRI* site flanked by sequences homologous to the *RIF1* target site. Importantly, we synonymously mutated one nucleotide in each PAM motif and three nucleotides in the “seed” region (3′ region) of the sense sgRNA sequence without disturbing the encoded amino acid sequence in order to prevent repetitive attack by Cas9 nickase after successful integration of the exogenous sequence (Fig. 7a), although in theory mutating one PAM/sgRNA sequence should be sufficient to preclude induction of a DSB. Two days after transfection, we sorted, with or without an EGFP-enrichment step, cells into 96-well plates at the level of a single cell per well (Supplementary Fig. 12a,c).

We performed genotypic screening of ensuing clones by PCR across the target locus with primers annealing outside the homologous region, followed by *EcoRI* digestion of individual PCR products. We found that through use of the sense or antisense strand ssODN donor, respectively, 20% (Fig. 7b) and 17.4% (Supplementary Fig. 12e) of EGFP-enriched clones were digestible by *EcoRI,* indicating the incorporation of the ssODN donors into one or both targeted alleles either precisely or in combination with indel formation (indicated by unexpected lengths of *EcoRI*-digested products). Through DNA sequencing, we found 4.4% and 2.2% for the sense ssODN and antisense ssODN donors, respectively, to be precise biallelic knock-ins (Fig. 7a, bottom panel and Supplementary Fig. 12f). By contrast, without an EGFP enrichment step, we were only able to detect around 1% of monoallelic knock-ins for either sense or antisense ssODN and did not identify any clones bearing a biallelic knock-in (Supplementary Fig. 12b,d).

Finally, to further examine the applicability of the knock-in system, we attempted to precisely mutate the *H2AFX* gene in a manner that would lead to Ser-139 of histone H2AX being replaced by Ala (H2AX^S139A^, Fig. 7c). H2AX Ser-139 is phosphorylated by DDR kinases such as ataxia telangiectasia mutated (ATM), DNA-dependent protein kinase (DNA-PK) and ATM-Rad3-related (ATR) at sites of DNA double-strand breaks^35^. Thus, mutating H2AX^S139A^ abolishes the formation of phosphorylated H2AX (γH2AX) foci in response to DNA damage. We transfected RPE-1 cells with *H2AFX*-targeted All-in-One Cas9^D10A^ nickase vector and either sense or antisense ssODN, then collected EGFP-enriched populations (Supplementary Fig. 13a,d). In addition to engineering the ODNs to mediate the Ser-139 to Ala mutation, we also mutated the PAM motif of the sense sgRNA sequence and the 3′ seed region of the antisense sgRNA sequence of the ssODN to prevent re-cutting by Cas9 nickase. Critically, to facilitate clonal genotypic screening of knock-ins, we also substituted two nucleotides in the ssODN template to create a *SmaI* restriction enzyme recognition site (CCCGGG) in the 3′ UTR of *H2AFX* (Fig. 7c). We carried out genotypic screening of EGFP-enriched clones by PCR across the target locus, followed by *SmaI* digestion to identify potential knock-ins (Supplementary Fig. 13). As shown in Fig. 7d, PCR products of clone 6G (in the sense-ssODN setup) and clone 7D (in the antisense-ssODN setup) were completely digested by *SmaI*. To confirm their genotypes, we performed long-range PCR (~4.6 kb) covering the upstream and downstream regions of the whole *H2AFX* gene, followed by *SmaI* digestion. This revealed that while clone 7D was successfully knocked-in at one allele with the other allele modified with a deletion (verified by DNA sequencing), clone 6G showed a biallelic knock-in genotype (Supplementary Fig. 14). We confirmed the precise knock-in alleles by DNA sequencing (Fig. 7c) and established that, unlike control cells, H2AX^S139A^ cells no longer formed γH2AX IRIF (Fig. 7e). Furthermore, consistent with MDC1 being recruited to DNA-damage sites via it binding directly to phosphorylated H2AX (γH2AX)^41^, MDC1 IRIF also did not form in H2AX^S139A^ cells (Fig. 7e). Western immunoblots also confirmed the absence of γH2AX formation upon irradiation of H2AX^S139A^ cells (Fig. 7f).

Collectively, these results demonstrated that employing the All-in-One Cas9^D10A^ nickase vector system with either sense or antisense ssODN as homologous templates, combined with a FACS-enrichment step followed by the rapid genotypic screening strategy, can establish biallelic knock-in clones in a highly efficient manner.

## Discussion

We have developed a versatile screening approach to rapidly generate isogenic knockout and knock-in clones in human cells. This work may thus considerably facilitate research aimed at defining gene and cellular functions, target validation from CRISPR-based or other screens, and work focused on providing deeper insights into underlying mechanisms of human disease. The approach we have used combines the All-in-One Cas9^D10A^ nickase vector system with a fluorescent marker FACS enrichment step, followed by high-throughput genotypic and/or phenotypic clonal screening strategies. The All-in-One vector system guarantees that each transfected cell can express all the elements essential for Cas9 nickase function at closely-juxtaposed target sites in order to generate a DSB. Furthermore, through 2A-coupled co-expression of fluorescent protein markers with Cas9^D10A^ nickase (1:1 ratio), the system allows the experimenter to assess uptake of the All-in-One-vector by measuring the level of EGFP or mCherry expression in cells. In agreement with a previous study^31^, we consistently found that FACS enrichment substantially increased knockout efficiency in all three cell lines tested in our work. Indeed, in the case of knock-in screening, we only obtained biallelic knock-ins when we employed FACS enrichment. Although single-cell FACS enrichment may not be possible for certain sensitive cell lines, this issue could be circumvented by using serial dilution to isolate single clones (for instance, to an average of 0.3 cells per well in a 96-well plate format) or by manual picking fluorescent-protein expressing clones. Compared with a conventional vector system that requires four plasmids each expressing Cas9 nickase, sense sgRNA, antisense sgRNA, or fluorescent marker (or drug selection donor vector), our system is simpler and maximises the molar capacity of the All-in-One vector in a single transfection reaction. It also exempts multiple plasmid preparation and, more importantly, obviates transfection of multiple vectors, which could potentially be cytotoxic for sensitive cell lines and increase the risk of undesirable random off-target genome integration of transfected plasmids. Moreover, the system we describe allowed us to use multi-channel FACS enrichment to select cells that had taken up all vectors with different fluorescent markers, thereby enhancing multiple gene targeting in one step. Compared to a previous report describing multiplex genome engineering using a similar all-in-one vector system^42^, our system possesses Cas9 nickases coupled with additional fluorescent protein markers that considerably enhance the success of gene knockout or knock-in experiments. We also noted that transfection and mutagenic efficiencies significantly decreased when we constructed the All-in-One plasmid with more than one sgRNA pair, increasing the size of the plasmid to more than 10 kb. Thus, we think that it will generally be preferable to co-transfect two or more All-in-One plasmids when conducting multiplex genome editing.

Some previous studies have indicated that Cas9 nickase tends to be less efficient than wild-type Cas9^16^,^18^,^29^. However, in our study we consistently observed that paired Cas9^D10A^ nickases displayed comparable or even higher mutagenic efficiency than wild-type Cas9, especially when using the All-in-One vector system. This apparent discrepancy may arise from use of different vector systems, since in most previous studies, use of the nickase system required one or more vectors as discussed above, which may compromise transfection capacity and efficiency. Indeed, as we compared the All-in-One dual sgRNA vector system with the single sgRNA vector system that required two vectors for one target (Figs 1 and 2) or four vectors for two targets (Supplementary Fig. 15), we consistently observed greater mutagenic efficiencies with the All-in-One dual sgRNA Cas9^D10A^ system. The difference between wild-type Cas9 and paired nickases is that the former creates blunt-end breaks and the latter produces “sticky ends” with overhanging DNA termini. It remains to be investigated how the DNA non-homologous end-joining repair machinery deals with different forms of DSBs and whether repairing sticky ends with overhangs of various types leads to enhanced/different mutagenesis than repairing blunt DNA DSBs. In this regard, we note that in line with previous reports^16^,^21^,^28^, when analysed using the All-in-One vector system, paired Cas9^D10A^ nickases creating a DSB with 5′ overhangs displayed substantially higher mutagenesis than paired Cas9^H840A^ nickases that induce 3′ overhangs.

There has been much attention focused on the specificity of the CRISPR-Cas9 system, and several methods have been developed to reduce undesired off-target effects. Here, we demonstrated that unlike the situation when we used wild-type Cas9 in the All-in-One vector system, use of Cas9^D10A^ nickase minimised off-target mutagenesis to undetectable levels while retaining high levels of on-target mutagenesis. We note that employing a nickase vector system to avoid potential off-target effects is straightforward and cost-effective, and owing to its relative simplicity, it should be readily adaptable to many genome-editing settings. In addition, we show that combining the All-in-One nickase vector system with genotypic and/or phenotypic screening strategies, facilitates the rapid generation and identification of intended knockouts and knock-ins in various human cell lines. We also show that there is good correlation between the outcomes of genotypic and phenotypic screening. Thus, while we found that parallel genotypic and phenotypic screening was most efficacious, whenever phenotypic screening is not possible (such as when studying an uncharacterised gene with unknown function, or no suitable high throughput phenotypic screening method is available) one can still readily identify candidate clones based on their initial genotyping outcome and then subsequently confirm their precise genotypes by DNA sequencing. We noticed that in genotypic screening of diploid RPE-1 and haploid HAP1 cells, more than two (RPE-1) or more than one (HAP1) bands of PCR products were detected in samples derived from some clones. This could arise from the possibility of two “sticky” single cells being plated in the same well during FACS sorting, and/or from a single sorted cell that started dividing before the targeted alleles were completely mutated by Cas9 nickases, whose transient expression could last for several days. In light of these potential issues, we avoided picking clones that possessed such allelic heterogeneity.

A limitation of deploying the double-nicking strategy in knock-in experiments is that a good pair of sgRNAs, ideally flanking the desired mutation, is essential for knock-in efficiency, as it has been shown that efficiency decreases as the desired mutation is moved further away from the sgRNA-targeted region^29^. One powerful aspect of site-specific knock-in gene editing is that it enables the experimenter to carry out detailed structure-function analyses of their gene of interest, model disease-associated mutations in cells or organisms, and to reverse mutations to wild-type. We have shown that we are able to establish biallelic knock-in cell lines by combining the All-in-One nickase vector system with a rapid genotypic screening strategy. By genotypic screening, we found that the majority of selected clones had undergone mutagenic DNA repair by end-joining as shown by indel formation (Supplementary Fig. 13), which is active throughout the whole cell cycle, while smaller proportions of clones carried out HDR, which only occurs at S and G2 phases of cell cycle when sister chromatids (or exogenous homologous templates if provided) are used as repair templates. It will be of interest to carry out further studies to find ways to enhance HDR-mediated processes, for example by altering cell cycle parameters^43^ and/or by inhibiting NHEJ or alternative end-joining processes^44^,^45^.

## Methods

### Cell culture

All cell lines were incubated at 37 °C in a humidified atmosphere containing 5% CO_2_. U2OS and HEK293FT cells were cultured in Dulbecco’s modified Eagle medium (DMEM; Sigma-Aldrich) supplemented with 10% (v/v) foetal bovine serum (FBS; BioSera), 100 U ml^−1^ penicillin (Gibco), 100 μg ml^−1^ streptomycin (Gibco) and 2 mM L-glutamine (Gibco), and for HEK293FT with 0.5 mg ml^−1^ G418 (Invitrogen). HAP1 cells were cultured in Iscove’s Modified Dulbecco’s Medium (IMDM; Sigma-Aldrich) supplemented as above. RPE-1 cells were cultured in DMEM and F-12 Ham mix medium (Sigma-Aldrich) supplemented as above and buffered with 0.2% sodium bicarbonate (Life Technologies).

### Construction of All-in-One nickase plasmids

Initially pSpCas9n-T2A-EGFP was purchased from Addgene (#48140). To create convenient 5′ overhangs for sgRNA cloning in our setting, we digested the plasmid with *BbsI* restriction enzyme (NEB) to insert phosphorylated and annealed oligos, 5′-CACCGGGGTCTTCGGATCCATGCGAAGACCT-3′ and 5′-AAACAGGTCTTCGCATGGATCCGAAGACCCC-3′. To insert another U6 promoter-sgRNA cassette, a PCR fragment was amplified from a pU6-sgRNA vector containing *BsaI* recognition sites for sgRNA cloning (a gift from Dr. W. Skarnes, the Wellcome Trust Sanger Institute, Cambridge, UK) using primers: 5′-CCGGTCTAGATTAACCCTCACTAAAGGGA-3′ and 5′-CCGGGGTACCAGAGATTTTGAGACACGGGCC-3′. The fragment was then digested with *XbaI* and *KpnI*, and ligated into the previous vector, pre-digested with the same restriction enzymes and gel-purified.

To generate the All-in-One nickase vector, with P2A-linked mCherry fluorescent marker, the All-in-One nickase vector with T2A-linked EGFP was digested with *BsaI* and subsequently ligated with phosphorylated and annealed oligos 5′-ACCGGGAGACCATCGATGAGAGGGTCTCA-3′ and 5′-AAACTGAGACCCTCTCATCGATGGTCTCC-3′, which substituted an unwanted *EcoRI* site with a unique *ClaI* site within the *BsaI* sgRNA cloning site. Next an overlap extension PCR was carried out to generate the P2A-mCherry fragment: first, since the original mCherry sequence contained a *BbsI* site that was also present in one of the sgRNA cloning sites, a synonymous mutation was introduced to disrupt the *BbsI* site in the mCherry sequence. To do so, primers 5′-CCCGGCCGGCCAGGCAAAAAAGAAAAAGGGCTCCGGAGCCACG-3′ and 5′-CAGCCCATGGTCTTTTTCTGCATTACGGGG-3′ were used to amplify fragment A (the 5′ part of P2A-mCherry), and oligos 5′-CCCGAATTCCTTGTACAGCTCGTCCATGC-3′ and 5′-CCCCGTAATGCAGAAAAAGACCATGGGCTG-3′ to amplify fragment B (the 3′ part of mCherry), using plasmid pMA-NeoR-P2A-mCherry (from our lab) as a template. Subsequently, primers 5′-CCCGGCCGGCCAGGCAAAAAAGAAAAAGGGCTCCGGAGCCACG-3′ and 5′-CCCGAATTCCTTGTACAGCTCGTCCATGC-3′ were used to obtain full-length P2A-mCherry (fragment C). Finally, fragment C was digested with *EcoRI* and *FseI*, and ligated into the All-in-One nickase vector whose *EcoRI* site in the *BsaI* sgRNA cloning sequence had previously been substituted by with a *ClaI* site as described above. DNA Sanger sequencing verified that all plasmid constructs were free of mutations. All-in-One-GFP and All-in-One-mCherry plasmids are available via Addgene (#74119 and #74120, respectively), and the full sequences are shown in Supplementary Fig. 16.

### sgRNA design and cloning

Design of sgRNAs: We used CLC Main Workbench 7 to download the full sequence of *Homo sapiens* chromosomes (GRCh38.p2 Primary Assembly) from NCBI, and located the genes of interest where their complete coding sequences of different isoforms were annotated. We then targeted the most 5′ exon that is shared among all splice variants (preferably the exon chosen is smaller than 200 bp in length and not a multiple of three if possible so that, under circumstances where such a targeted exon is skipped during alternative splicing, it will lead to out-of-frame mutations in the following exons). The exon sequence can be further verified on the Ensembl website (http://www.ensembl.org). The design of optimal sgRNA pairs for nickase targeting and prediction of off-target sites were based on online tools: CRISPR Design (http://crispr.mit.edu/) and WTSI Genome Editing (http://www.sanger.ac.uk/htgt/wge/). We found both sites equally useful in terms of choosing the best sgRNA pairs where different pair candidates are ranked based on predicted off-target effects. It is up to the experimenter to decide which online tool to use. Essentially, we avoided sgRNA pairs with negative offset distances (sgRNA sequences overlapping with each other) and found that offset distances between 0–30 bp work well. The experimenter can select a pair simply by looking for PAM sequences (NGG) within the targeting region and then assessing their off-target effects using the online tools mentioned above. For instance, we used sgRNAs with medium to high quality scores from CRISPR Design. In our studies, we used only one sgRNA pair in each instance, and in every case we had success. However, if the experimenter has doubts about which of alternative pairs to use, several pairs of sgRNAs could be tested by T7 EI assays in HEK293T cells to compare their mutagenic efficiencies.

Golden Gate sgRNA cloning: All-in-One nickase vector plasmids were digested with *BbsI* and then dephosphorylated by Alkaline Phosphatase, Calf Intestinal (CIP, NEB). The digested vectors were recovered from 0.8% low-melting temperature agarose gels, and melted at 65 °C for subsequent ligation reactions. Pairs of complementary DNA oligos (4-mer overhang+20-mer of sgRNA sequence; forward and reverse oligos are shown in Supplementary Table 1) were purchased in standard desalted format from Sigma-Aldrich. The DNA oligonucleotides were individually phosphorylated (T4 Polynucleotide Kinase, NEB) and then annealed by pooling together in a thermocycler machine using the following program: 95 °C for 1 minute, followed by slow cooling from 95 °C to 4 °C at a ramp rate of 0.03 °C s^−1^. Each DNA oligo duplex had 5′ overhangs (forward: ACCG, reverse: AAAC) designed to be directly cloned into the *BbsI* or *BsaI*-digested All-in-One vector plasmids. The first DNA oligonucelotide duplex was ligated into *BbsI*-digested All-in-One vectors using the Quick Ligation Kit (NEB). The successful clones should lose a unique *BamHI* recognition site that can be confirmed by *BamHI* digestion alongside with a control of an empty vector. To clone the second DNA oligonucelotide duplex, the same procedure was followed apart from using *BsaI* to digest the All-in-One plasmids. The successful clones should lose a unique *ClaI* recognition site that can be confirmed by *ClaI* digestion alongside with a control of an empty vector. Both sgRNA sequences were verified by DNA Sanger sequencing in a single reaction using a primer: 5′-CTTGATGTACTGCCAAGTGGGC-3′.

### T7 endonuclease I (T7 EI) assay

Cells were seeded in 96-well plates and transfected using either the Neon Transfection System (Life Technologies) or the FuGENE HD Transfection Reagent (Promega) according to the manufacturer’s instructions. Genomic DNA was extracted 3 days after transfection for PCR. PCRs were performed across on- or off-target sites with site-specific primers (Supplementary Table 2). PCR products were purified using QIAquick PCR Purification Kit (Qiagen) and then quantified. The T7 endonuclease I assay was carried out according to the manufacturer’s instructions (NEB, M0302). In brief, 200 ng of amplified PCR products were reannealed to form heteroduplex in 1X NEBuffer 2 using the following program: denaturation at 95 °C for 5 min, reannealing from 95 °C to 85 °C at −2 °C s^−1^, hold at 85 °C for 1 min, cooling from 85 °C to 25 °C at −0.1 °C s^−1^, and hold at 25 °C for 1 min, followed by cooling down to 4 °C. The samples were then subjected to T7 EI at 37 °C for 15 min and analysed on agarose gels. The gels were imaged and quantified by Geldoc XR+ system (Biorad). Indel occurrence was calculated using the following formula as described^5^: indel (%) = 100 × (1−(1 − *f*_cut_)^1/2^). *f*_cut_ is the fraction of the cleaved PCR product.

### DNA oligos and long ssODN

All the DNA oligos used in this study were purchased Sigma-Aldrich, except that for knock-in experiments, PAGE-purified ultramer ssODNs were purchased from Integrated DNA Technologies (IDT).

### Transfection, drug and ionising radiation (IR) treatments

Plasmids were transfected into cells by electroporation using the Neon Transfection System according to the manufacturer’s instructions (Life Technologies). In brief, 10 μg of the All-in-One plasmid were transfected into 1.2 million cells at 1400 Volts in a single 30-millisecond pulse. For knock-in, ssODNs were dissolved in TE buffer and transfected at 0.1 nmol per reaction together with 10 μg of the All-in-One plasmid. Drug treatments: Remodelin was resuspended in DMSO, diluted to 50 μM in DMEM media and added to the cells for 16 hours as described previously^39^. Nutlin-3 (Sigma) was resuspended in DMSO and diluted to 10 μM in DMEM/F12-Ham medium. IR treatment: cells were treated with either 2 Gy or 10 Gy of IR as indicated by Faxitron-CellRad (Faxitron Bioptics, LLC).

### Fluorescence-activated cell sorting (FACS)

Cells were trypsinised, washed with PBS, resuspended in PBS (supplemented with 3% BSA) and filtered through 50 μm CellTrics (Partec). Cells were individually sorted, either based on EGFP, mCherry+EGFP or sorted regardless of colour (MoFlo, Becton Dickinson) into tissue culture treated 96-well plates (Thermo Scientific) at a single-cell-per-well density for clonal expansion. Viable clones were subsequently assembled in new 96-well plates for screening purposes.

### Nuclear circularity and nuclear area quantification

CellProfiler software was used to quantify nuclear circularity and nuclear area from DAPI staining pictures, using the “object size shape” measurement. The AreaShape measurement allowed the calculation of the Form Factor index (4 × π × Area/Perimeter^2^) corresponding to circularity (a Form Factor of 1 reflecting a perfect circle), as well as the calculation of nuclear area.

### Quantitative RT-PCR

Total RNA was extracted from RPE-1 wild-type and *TP53* knockout cells using RNeasy mini kit (Qiagen), according to the manufacturer’s instructions. RNA was resuspended in DEPC‐treated H_2_O. Synthesis of cDNA with Superscript III reverse transcriptase (Invitrogen) was primed with oligo (dT). Primers for *TP53* (set 1: Fwd-CCCTTCCCAGAAAACCTACC and Rev-CTCCGTCATGTGCTGTCGACT, set 2: Fwd-TCTTCTGTCCCTTCCCAGAA and Rev-CTCACAACCTCCGTCATGTG) and *β*-actin (Fwd-CCTGGCACCCAGCACAAT and Rev-GGGCCGGACTCGTCATACT) were designed to amplify 100–200 bp fragments. Analyses were carried out using SYBR Green PCR master mix (Applied Biosystems) on a 7300 Real-Time PCR system (Applied Biosystems). Amounts of target mRNA were normalized to an endogenous housekeeping gene (*β*-actin) and calculated using the formula: 2^−ΔΔCT^.

### Genomic DNA extraction

For genotyping, genomic DNA was extracted from clones in a 96-well plate format using QuickExtract DNA Extraction Solution (Epicentre) according to the manufacturer’s protocol. Briefly, cells were washed with 200 μl PBS and incubated in 50 μl DNA extraction solution for 10 minutes at 37 °C. The lysates were then transferred to a new 96-well PCR plate, agitated thoroughly and incubated at 65 °C for 6 minutes. After 15 seconds of vortex mixing, the samples were heated to 98 °C for 2 minutes, followed by cooling down to 4 °C.

### PCR-genotyping and DNA sequencing

All PCRs in this study were performed with Phusion Hot Start II High-Fidelity DNA Polymerase (F549; Thermo Scientific) according to the manufacturer’s instructions. PCR products were separated on agarose gels and anaylsed by Geldoc XR+ system (Biorad). A list of the PCR primers used for genotypic screening or DNA sequencing is shown in Supplementary Table 3. PCR products were either directly sequenced by using the gene-specific primers (for haploid HAP1), or, to distinguish on-target mutations for each individual allele, cloned into pCR4 Blunt-TOPO vectors (ThermoFisher), followed by DNA sequencing using either T7 or T3 primers.

### Immunoblotting

Proteins were extracted with SDS lysis buffer (4% SDS, 20% glycerol, and 120 mM Tris-HCl, pH 6.8). Samples were then heated to 95 °C for 5 minutes, followed by shearing with 10 strokes through 25-gauge needles. Protein concentrations were determined with a NanoDrop spectrophotometer (Thermo Scientific) at 280 nm followed by addition of bromophenol blue and 2-mercaptoethanol. After 5 min at 95 °C, SDS–PAGE was performed to resolve proteins using the Novex NuPAGE SDS-PAGE gel system (Life Technologies), and then transferred to nitrocellulose or PVDF membranes (GE Healthcare). Membranes were stained with Ponceau S to confirm homogeneous loading, followed by hybridisation with specific primary antibodies and incubation with appropriate secondary antibodies. Antibodies used in this study are listed in Supplementary Table 4.

### IF cell-staining and automated high-throughput microscopy-based screening

Cells were washed with PBS containing 0.1% Tween-20 (PBST) and fixed for 15 min in PBS containing 2% paraformaldehyde. After fixation, cells were permeabilised in PBS containing 0.2–0.5% Triton X-100 for 10–15 min and blocked with blocking buffer (PBST with 5% (w/v) BSA) for 30 min. After three washes with PBST, immunostaining was performed with the indicated primary antibodies (Supplementary Table 4) diluted in blocking buffer for 1 h at room temperature followed by three washes with PBST. Cells were then stained with the appropriate secondary antibodies for 1 h at room temperature diluted in blocking buffer followed by another three washes with PBST. Cells were counterstained with DAPI (1 μg ml^−1^) and mounted using Vectashield (Vector Labs). Images were acquired with a FluoView 1000 confocal microscope (Olympus). For high-throughput microscopy screening for DDR gene knockouts, clones in glass-bottom 96-well imaging plates (Swissci) were treated with 2 Gy of IR prior to the IF cell-staining as described above. A spinning-disc Perkin Elmer Opera platform equipped with a 20x water immersion objective (0.7 numerical aperture, Olympus) was employed to acquire 3 confocal images (fields) for each well in a single optimised focal plane for each of the three fluorescence channels, DAPI (405 nm), Alexa Fluor 488 and Alexa Fluor 594.

## Additional Information

**How to cite this article**: Chiang, T.-W. W. *et al.* CRISPR-Cas9^D10A^ nickase-based genotypic and phenotypic screening to enhance genome editing. *Sci. Rep.* **6**, 24356; doi: 10.1038/srep24356 (2016).

## Supplementary Material

Supplementary Information
