# Supplementary material for: CRISPR-Cas9D10A nickase-based genotypic and phenotypic screening to enhance genome editing
Source: Sci Rep. 2016 Apr 15;6:24356. doi: 10.1038/srep24356 (PMC4832145; doi:10.1038/srep24356)
Supplement: Supplementary Information [file srep24356-s1.pdf]

CRISPR-Cas9^{D10A} nickase-based genotypic and phenotypic screening to enhance genome editing

Ting-Wei Will Chiang[1,2,4], Carlos le Sage[1,4], Delphine Larrieu[1], Mukerrem Demir[1] and Stephen P. Jackson[1,2,3]

[1]Wellcome Trust/Cancer Research UK Gurdon Institute, University of Cambridge, Cambridge CB2 1QN, UK. [2]Department of Biochemistry, University of Cambridge, Cambridge CB2 1GA, UK. [3]The Wellcome Trust Sanger Institute, Hinxton, Cambridge CB10 1SA, UK.

[4]These authors contributed equally to this work. Correspondence should be addressed to Stephen P. Jackson (s.jackson@gurdon.cam.ac.uk)
Keywords: CRISPR-Cas9, nickase, genotypic screening, phenotypic screening, DNA damage response

Figure S1

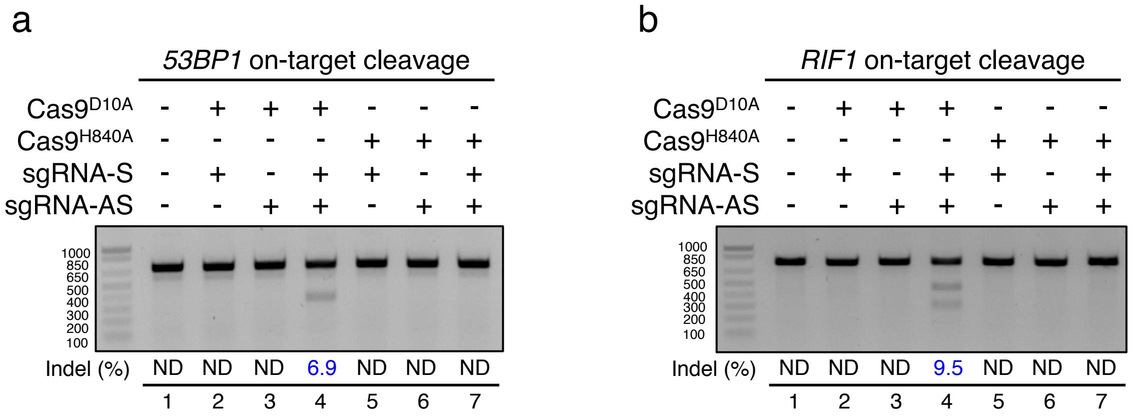

Supplementary Figure 1 Mutagenic efficiencies of Cas9^{D10A} vs. Cas9^{H840A}

On-target mutagenic efficiencies by Cas9^{D10A} or Cas9^{H840A} nickases at the *53BP1* (**a**) or the *RIF1* (**b**) locus in human HEK293T cells. Cells were either transfected with or without (lane 1) single sgRNA Cas9^{D10A} nickase vector containing either the S (lane 2) or the AS sgRNA (lane 3), the All-in-One Cas9^{D10A} nickase vector (lane 4), single sgRNA Cas9^{H840A} nickase vector carrying either the S sgRNA (lane 5) or the AS sgRNA (lane 6), or the All-in-One Cas9^{H840A} nickase vector (lane 7).

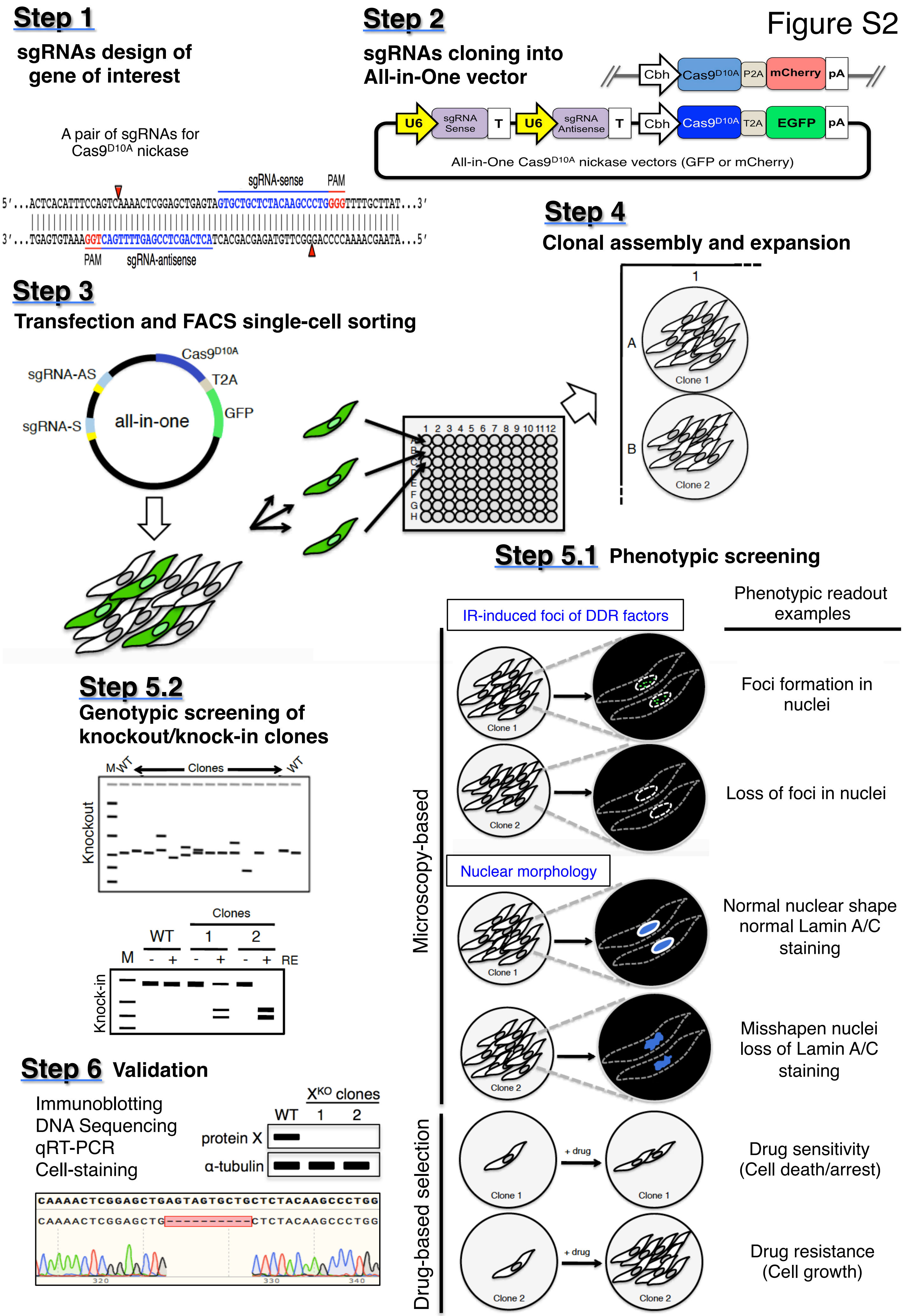

Figure S2
Step 1
sgRNAs design of gene of interest
A pair of sgRNAs for Cas9D10A nickase
sgRNA-sense
PAM
5'...ACTCACATTTCCAGTCAAAACTCGGAGCTGAGTAGTGCTGCTCTACAAGCCCTGGGGTTTTGCTTAT...3'
3'...TGAGTGTAAAGGTCAGTTTTGAGCCTCGACTCATCACGACGAGATGTTCGGGACCCCAAAACGAATA...5'
PAM
sgRNA-antisense
Step 2
sgRNAs cloning into All-in-One vector
Cbh
Cas9D10A
P2A
mCherry
pA
U6
sgRNA Sense
T
U6
sgRNA Antisense
T
Cbh
Cas9D10A
T2A
EGFP
pA
All-in-One Cas9D10A nickase vectors (GFP or mCherry)
Step 3
Transfection and FACS single-cell sorting
Cas9D10A
sgRNA-AS
T2A
GFP
sgRNA-S
all-in-one
1 2 3 4 5 6 7 8 9 10 11 12
A B C D E F G H
Step 4
Clonal assembly and expansion
1
A
B
Clone 1
Clone 2
Step 5.1 Phenotypic screening
Phenotypic readout examples
IR-induced foci of DDR factors
Microscopy-based
Clone 1
Foci formation in nuclei
Clone 2
Loss of foci in nuclei
Nuclear morphology
Clone 1
Normal nuclear shape normal Lamin A/C staining
Clone 2
Misshapen nuclei loss of Lamin A/C staining
Drug-based selection
Clone 1
+ drug
Clone 1
Drug sensitivity (Cell death/arrest)
Clone 2
+ drug
Clone 2
Drug resistance (Cell growth)
Step 5.2
Genotypic screening of knockout/knock-in clones
M WT
Clones
WT
Knockout
Clones
WT
1
2
M
- + - + - +
RE
Knock-in
Step 6 Validation
Immunoblotting
DNA Sequencing
qRT-PCR
Cell-staining
XKO clones
WT 1 2
protein X
α-tubulin
CAAAACTCGGAGCTGAGTAGTGCTGCTCTACAAGCCCTGG
CAAAACTCGGAGCTG----------CTCTACAAGCCCTGG
320
330
340

Figure S2

Supplementary Figure 2 Schematic of $Cas9^{D10A}$ nickase-based knockout and knock-in screening strategies.

Step 1: Design of a pair of sgRNAs for $Cas9^{D10A}$ nickase-based genome editing for gene of interest. Nicks on each strand of DNA generated by a $Cas9^{D10A}$ nickase mimic a double-strand break which can be repaired by NHEJ or HR.

Step 2: Cloning sense and antisense sgRNA sequences into the all-in-one vector containing $Cas9^{D10A}$ nickase coupled with either EGFP or mCherry via a “self-cleaving” 2A peptide linker.

Step 3: Cells were transfected with all-in-one vectors and two to three days after, FACS single-cell sorting into 96-well plates was performed based on the fluorescent protein marker (EGFP or mCherry).

Step 4: Clones were grouped and expanded in a 96-well format.

Step 5.1: High-throughput phenotypic screening approaches to assess knockout or knock-in mutational status of individual clones in a 96-well format. Shown examples are screens performed in this work, including microscopy-based IF cell-staining and drug-based selection strategies.

Step 5.2: Genomic DNA extraction of individual clones from 96-well plates and genotypic screening by PCR across target loci. Separation of PCR products by high-percentage agarose gel electrophoresis reveals clones with biallelic indel mutations as potential knockout (top gel) or with RE-digested alleles as potential knock-in (bottom gel) candidates. RE, restriction endonuclease.

Step 6: Validation of knockout or knock-in candidate clones by immunoblotting, immunostaining, qRT-PCR and on-target DNA sequencing.

Figure S3

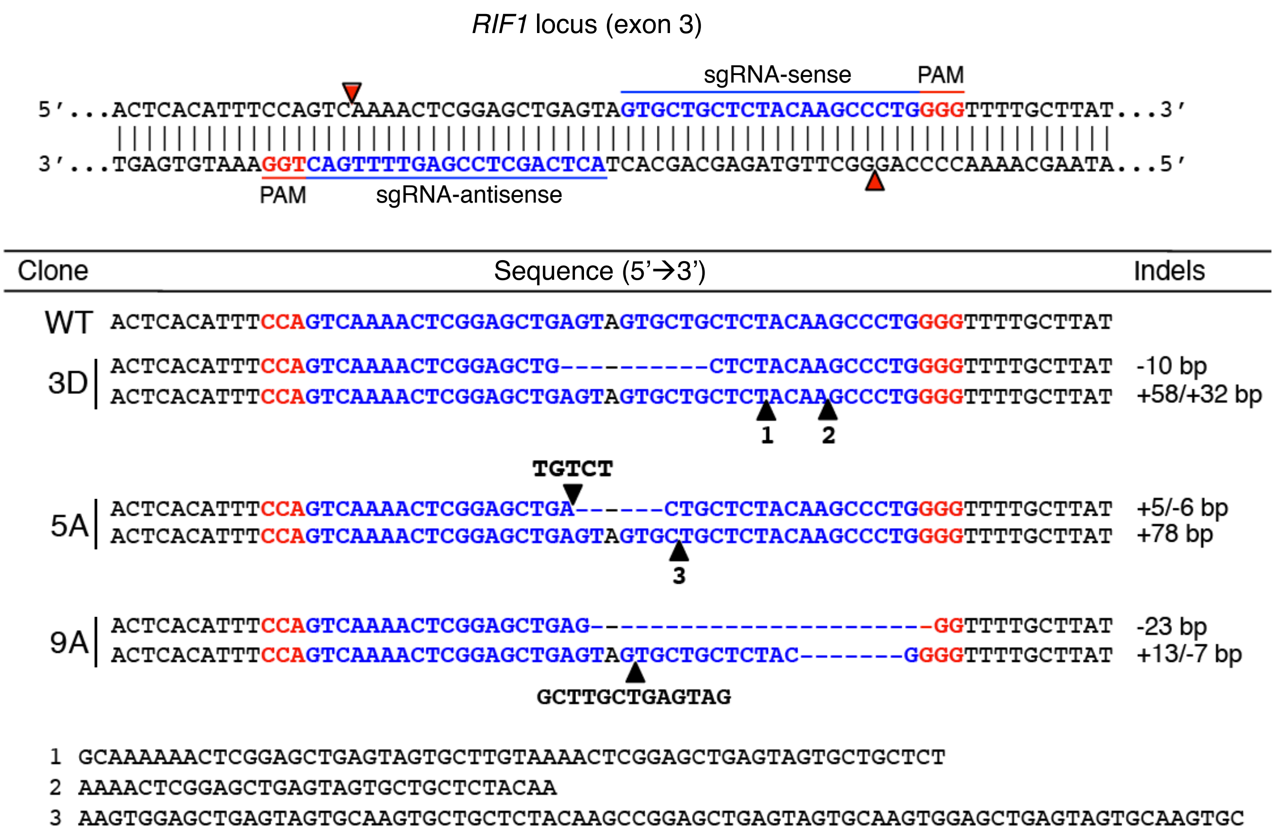

Supplementary Figure 3 Sequencing of RIF1 knockout clones in RPE-1 cells.

On-target sequence of the *RIF1* locus (exon 3) where PAM sequences are indicated in red and sgRNA target sequences in blue. Red arrowheads indicate the Cas9^{D10A} nicking sites. Each sequence represents one allele. Dashed lines indicate deletions; black arrowheads indicate the precise locations of insertions. Inserted sequences are either tagged with black arrowheads, or numbered and shown below. The exact sizes of indels for each allele are shown on the right.

Figure S4

a

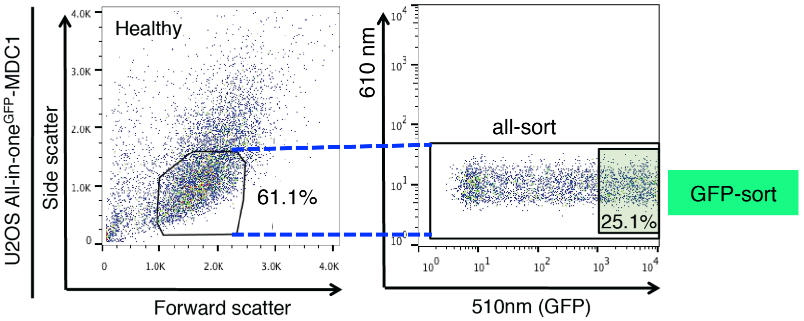

b

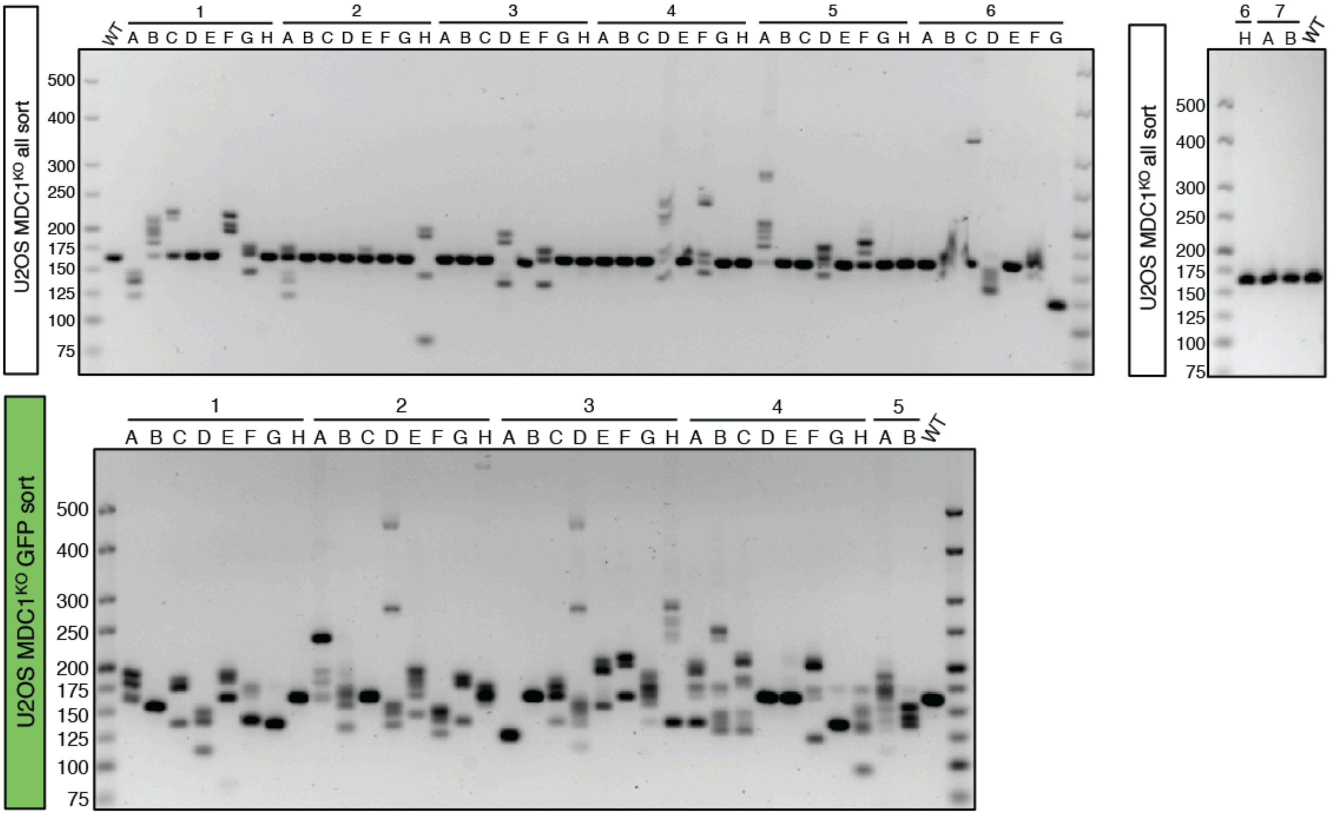

c

MDC1 locus (exon 9)

sgRNA-sense PAM

5'...GCCTCCTGAATTGCAAGATGCCACCTGCTGAGAAGGCTTCCAGGATCAGAGCTGCTGAGAAGGTTTCCAGG...3'

3'...CGGAGGACTTAACGTTCTACGGTGGACGACTCTTCCGAAGGTCCTAGTCTCGACGACTCTTCCAAAGGTCC...5'

PAM sgRNA-antisense

Clone	Sequence (5' → 3')	Indels
WT	GCCTCCTGAATTGCAAGATGCCACCTGCTGAGAAGGCTTCCAGGATCAGAGCTGCTGAGAAGGTTTCCAGG	
1C	GCCTCCTGAATTGCAAGATGCCACCTGCTGAGAAGGCTTCCAGGATCAGAGCTGCTGAGAAGGTTTCCAGG (insertion: GCAAGATGCCA)	+11 bp
	GCCTCCTGAATTGC-----------------------------CAGGATCAGAGCTGCTGAGAAGGTTTCCAGG	-26 bp
2A	---------------------------------TTCCAGGATCAGAGCTGCTGAGAAGGTTTCCAGG	-267 bp
	GCCTCCTGAATTGCAAGATGCCACCTGCTGAGAAGGCTTCCAGGATCAGAGCTGCTGAGAAGGTTTCCAGG (insertions: 1, 2)	+46/+27 bp
3E	GCCTCCTGAATTGCAAGATGCCACCTGCTGAGAAGGCTTCC-----------TGCTGAGAAGGTTTCCAGG	-11 bp
	-------------------------------AAGGCTTCCAGGATCAGAGCTGCTGAGAAGGTTTCCAGG	-189 bp
	GCCTCCTGAATTGCAAGATGCCACCTGCTGAGAAGGCTTCCAGGATCAGAGCTGCTGAGAAGGTTTCCAGG (insertion: 3)	+29 bp

1 CCTGCAAGATGCCACCTGCTGAGAAGGCTTCCAGGATCAGAGCTGC

2 CTTCCAGGATCAGAGCTGCTGAGAAGG

3 TGAGAAGGCTTCCAGGATCAGAGCTGCTG

Figure S4

Supplementary Figure 4 *MDC1* knockout in U2OS cells.

(**a**) FACS data showing the sequential gating of healthy (all-sort, left panel) and high-EGFP expressing (GFP-sort, right panel) cell populations. Each of these populations was sorted into 96-well plates at a single-cell-per-well density. (**b**) PCR-genotyping of individual clones from all- and GFP-sort populations. WT product size is 164 bp. (**c**) On-target sequence of the *MDC1* locus (exon 9) where PAM sequences are indicated in red and sgRNA target sequences in blue. Red arrowheads indicate the Cas9^{D10A} nicking site. Each sequence represents one allele in the knock-out clones. Dashed lines indicate deletions; black arrowheads indicate the precise locations of insertions. Inserted sequences are either tagged with black arrowheads, or numbered and shown below. The exact sizes of indels for each allele are shown on the right.

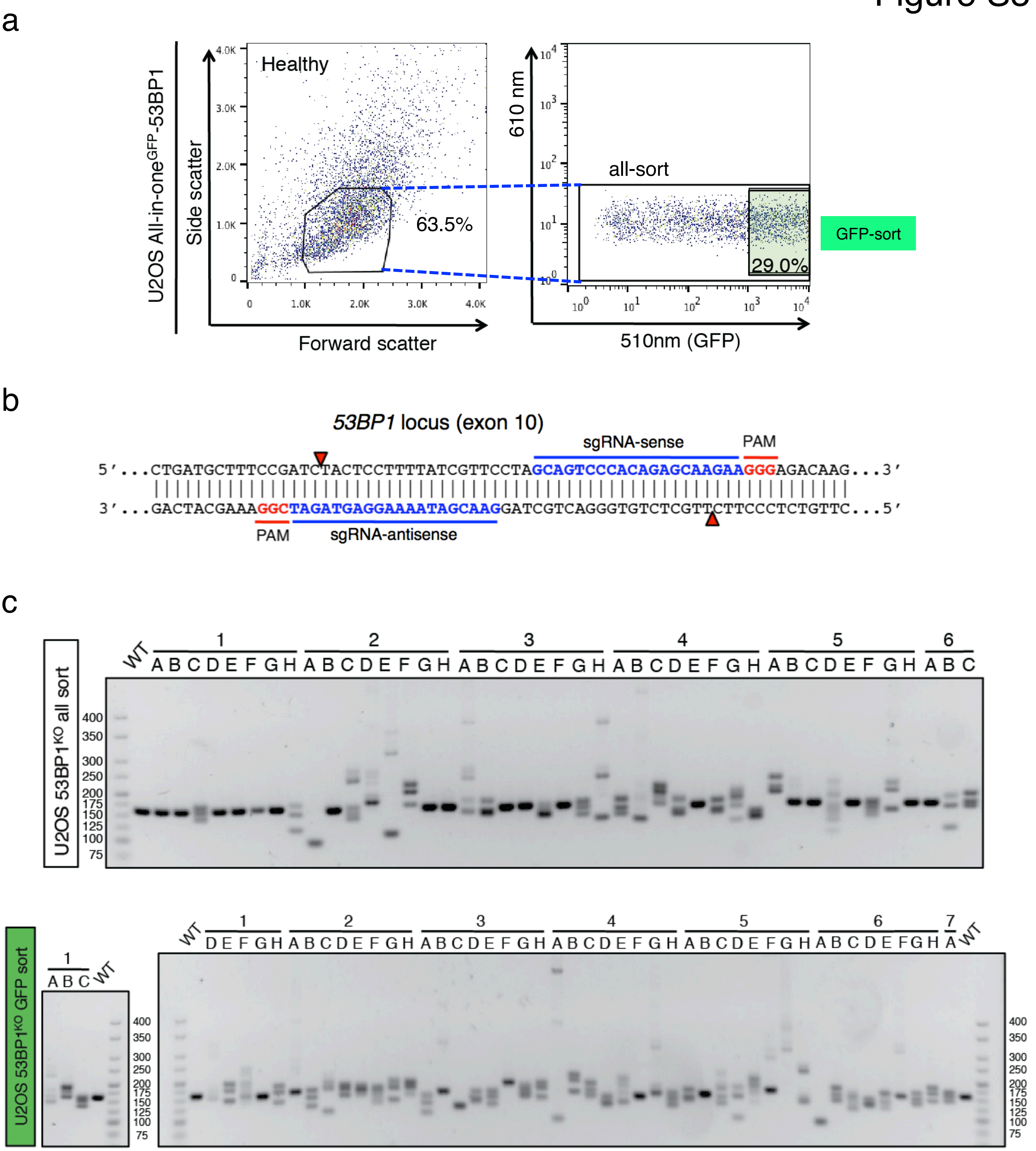

Supplementary Figure 5 *53BP1* knockout in U2OS cells.

(**a**) FACS data showing the sequential gating of healthy (all-sort, left panel) and high-EGFP expressing (GFP-sort, right panel) cell populations. Each of these populations was sorted into 96-well plates at a single-cell-per-well density. (**b**) Schematic of sgRNA target sequences at exon 10 of the *53BP1* locus. PAM sequences are indicated in red and sgRNA target sequences in blue. Red arrowheads indicate the Cas9^{D10A} nicking sites. (**c**) PCR-genotyping of individual clones from all- and GFP-sort populations. WT product size is 162 bp.

Figure S6

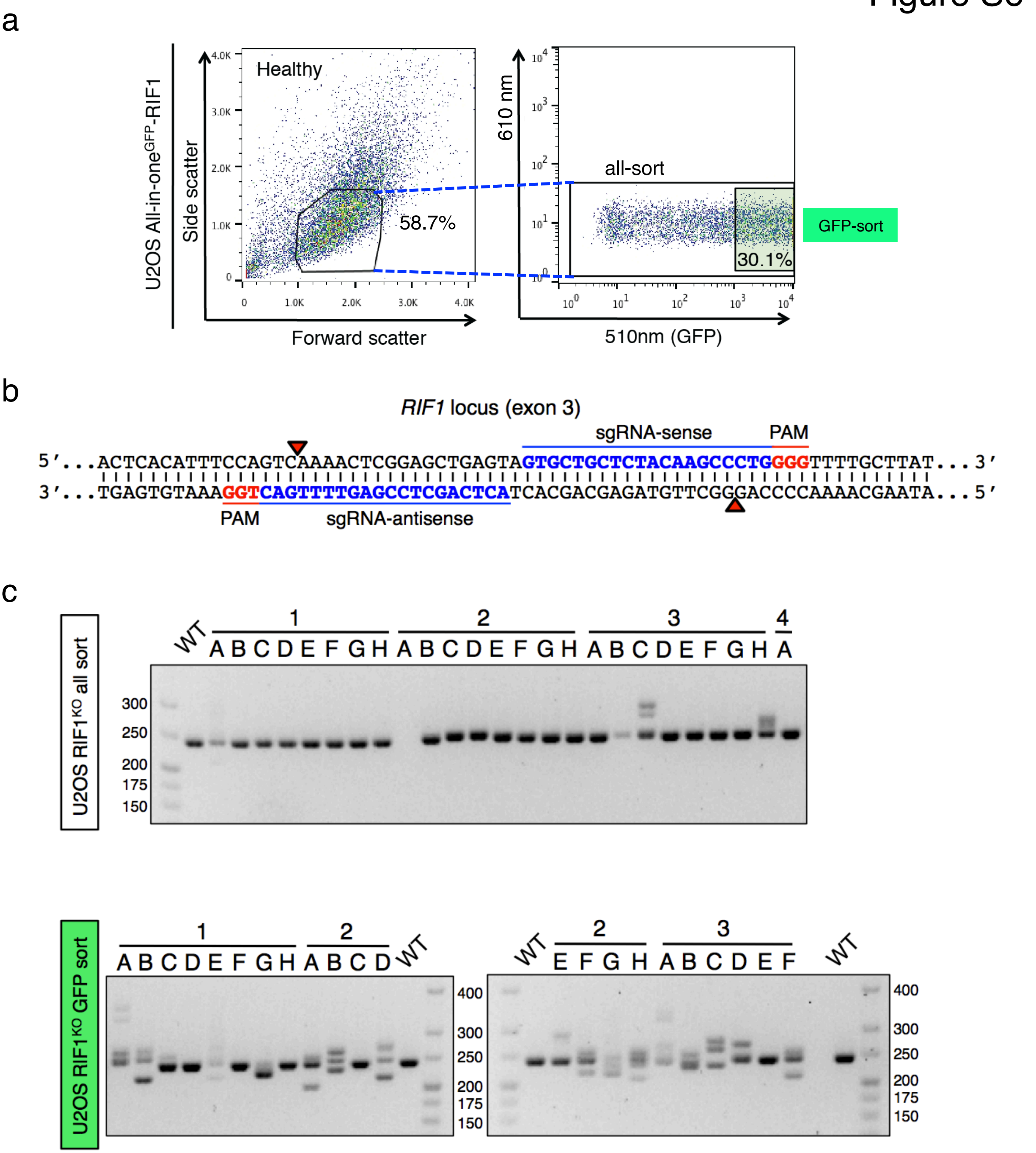

Supplementary Figure 6 *RIF1* knockout in U2OS cells.

(**a**) FACS data showing the sequential gating of healthy (all-sort, left panel) and high-EGFP expressing (GFP-sort, right panel) cell populations. Each of these populations was sorted into 96-well plates at a single-cell-per-well density. (**b**) Schematic of sgRNA target sequences at exon 3 of the *RIF1* locus. PAM sequences are indicated in red and sgRNA target sequences in blue. Red arrowheads indicate the Cas9^{D10A} nicking sites. (**c**) PCR-genotyping of individual clones from all- and GFP-sort populations. WT product size is 244 bp.

Figure S7

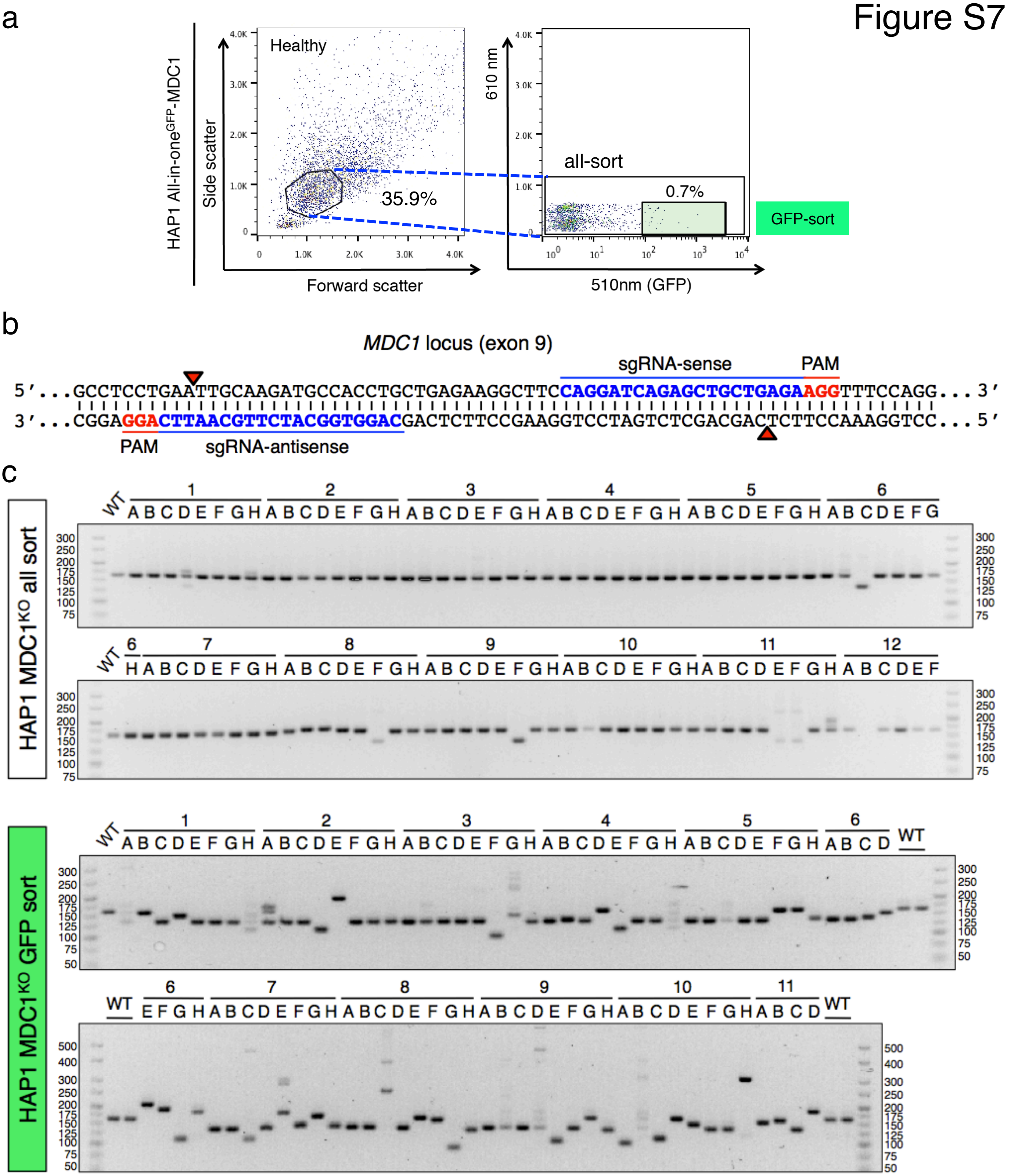

Supplementary Figure 7 *MDC1* knockout in HAP1 cells.

(**a**) FACS data showing the sequential gating of healthy (all-sort, left panel) and high-EGFP expressing (GFP-sort, right panel) cell populations. Each of these populations was sorted into 96-well plates at a single-cell-per-well density. (**b**) Schematic of sgRNA target sequences at exon 9 of the *MDC1* locus. PAM sequences are indicated in red and sgRNA target sequences in blue. Red arrowheads indicate the Cas9^{D10A} nicking sites. (**c**) PCR-genotyping of individual clones from all- and GFP-sort populations. WT product size is 164 bp.

Figure S8

a

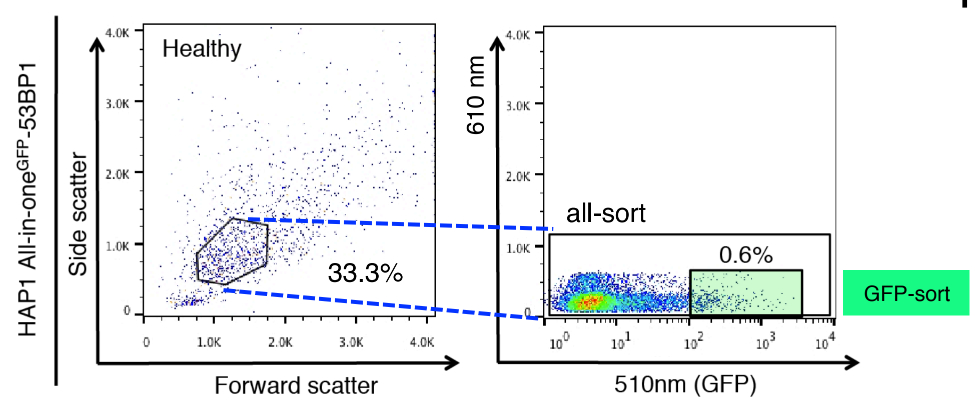

b

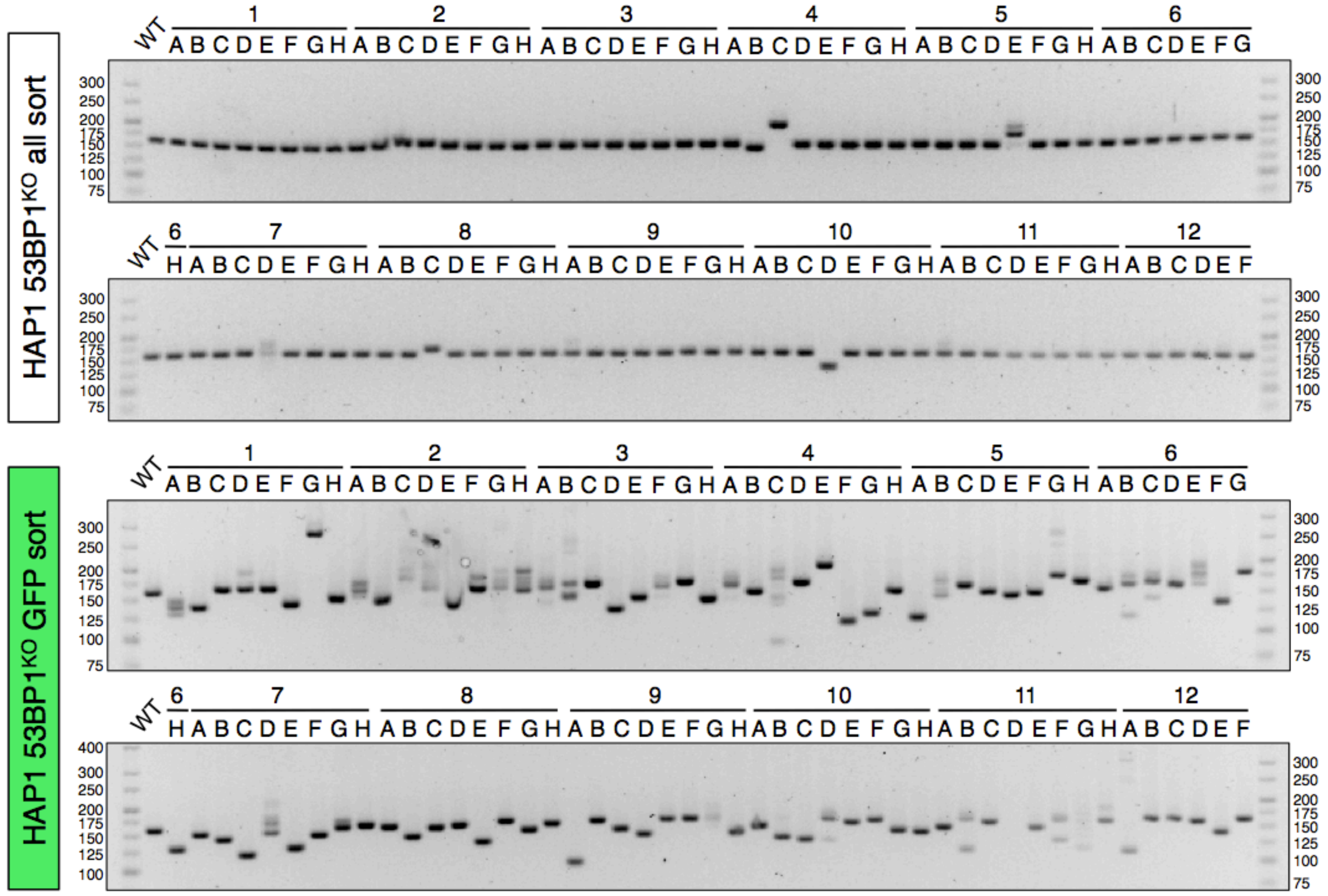

c

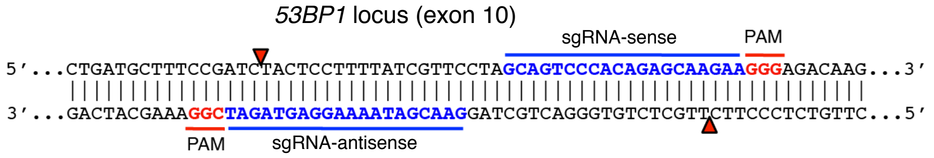

Clone	Sequence (5' → 3')	Indels
WT	CTGATGCTTTCCGATCTACTCCTTTTATCGTTCCTAGCAGTCCCACAGAGCAAGAAGGGAGACAAG	
1C	CTGATGCTTTCCGATCTACTCCTTTTATCGTTCCTAGCAGTCCCACAGAGCAAGAAGGGAGACAAG	+4 bp (CAAG)
3G	CTGATGCTTTCCGATCTACTCCTTTTATCGTTCCTAGCAGTCCCACAGAGCAAGAAGGGAGACAAG	+7 bp (AGAGCAA)
8E	CTGATGCTTTCCGATC----------------------------ACAGAGCAAGAAGGGAGACAAG	-28 bp
9A	CTGA--CAAG	-58 bp
10F	CTGATGCTTTCCGATCTACTCCTTTTATCGTTCCTAGCAGTCCCACAGAGCAAGAAGGGAGACAAG	+4 bp (CAAG)
10G	CTGATGCTTTCCGATCTACTCCTTTTATCGTTC--------------GAGCAAGAAGGGAGACAAG	-14 bp

Figure S8

Supplementary Figure 8 *53BP1* knockout in HAP1 cells.

(**a**) FACS data showing the sequential gating of healthy (all-sort, left panel) and high-EGFP expressing (GFP-sort, right panel) cell populations. Each of these populations was sorted into 96-well plates at a single-cell-per-well density. (**b**) PCR-genotyping of individual clones from all- and GFP-sort populations. WT product size is 162 bp. (**c**) On-target sequence of the *53BP1* locus (exon 10) where PAM sequences are indicated in red and sgRNA target sequences in blue. Red arrowheads indicate the $Cas9^{D10A}$ nicking site. Each sequence represents one allele in the knock-out clones. Dashed lines indicate deletions; black arrowheads indicate the precise locations of insertions. Inserted sequences are tagged with black arrowheads. The exact sizes of indels for each allele are shown on the right.

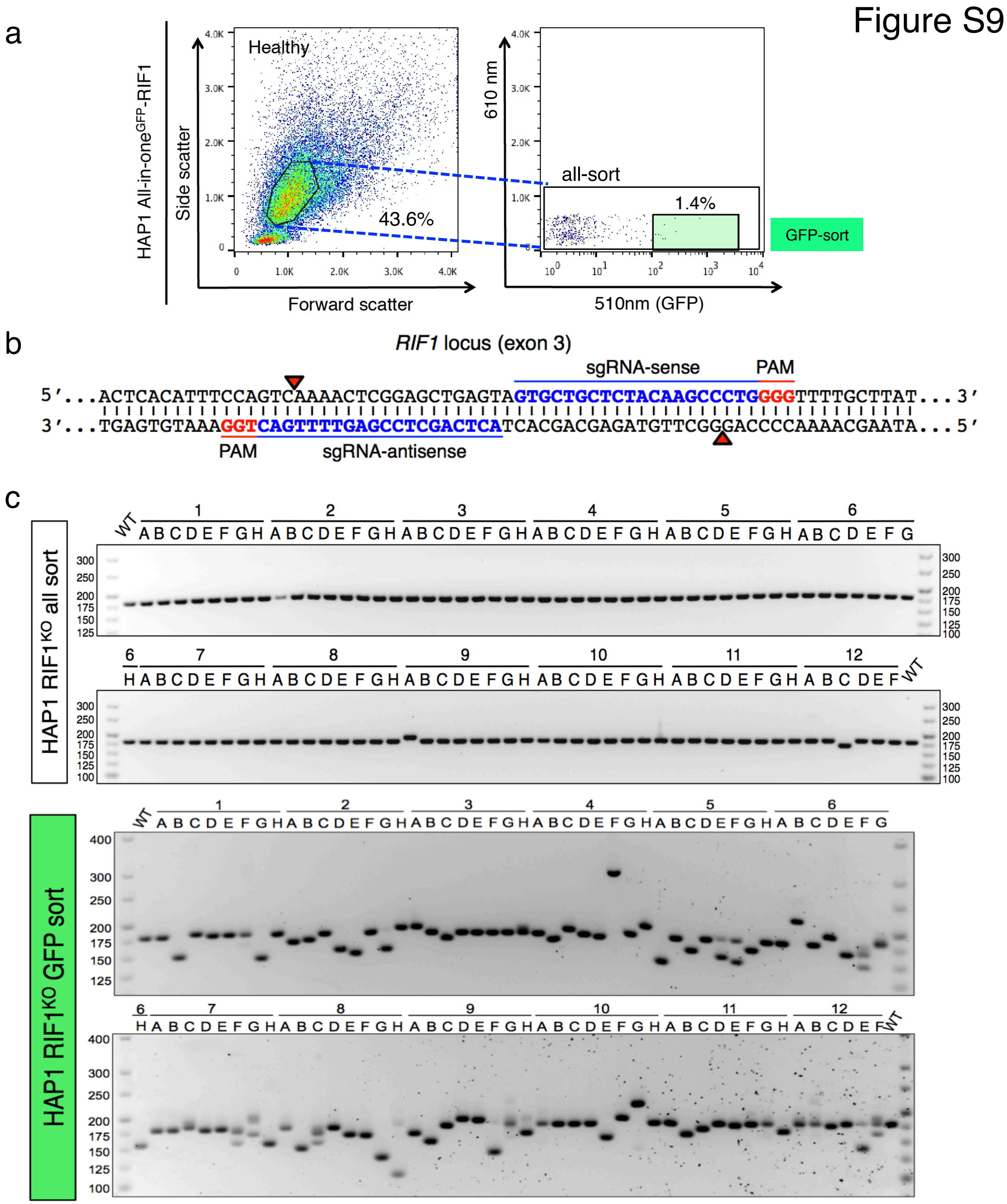

Supplementary Figure 9 *RIF1* knockout in HAP1 cells.

(**a**) FACS data showing the sequential gating of healthy (all-sort, left panel) and high-EGFP expressing (GFP-sort, right panel) cell populations. Each of these populations was sorted into 96-well plates at a single-cell-per-well density. (**b**) Schematic of sgRNA target sequences at exon 3 of the *RIF1* locus. PAM sequences are indicated in red and sgRNA target sequences in blue. Red arrowheads indicate the Cas9^{D10A} nicking sites. (**c**) PCR-genotyping of individual clones from all- and GFP-sort populations. WT product size is 182 bp.

Figure S10

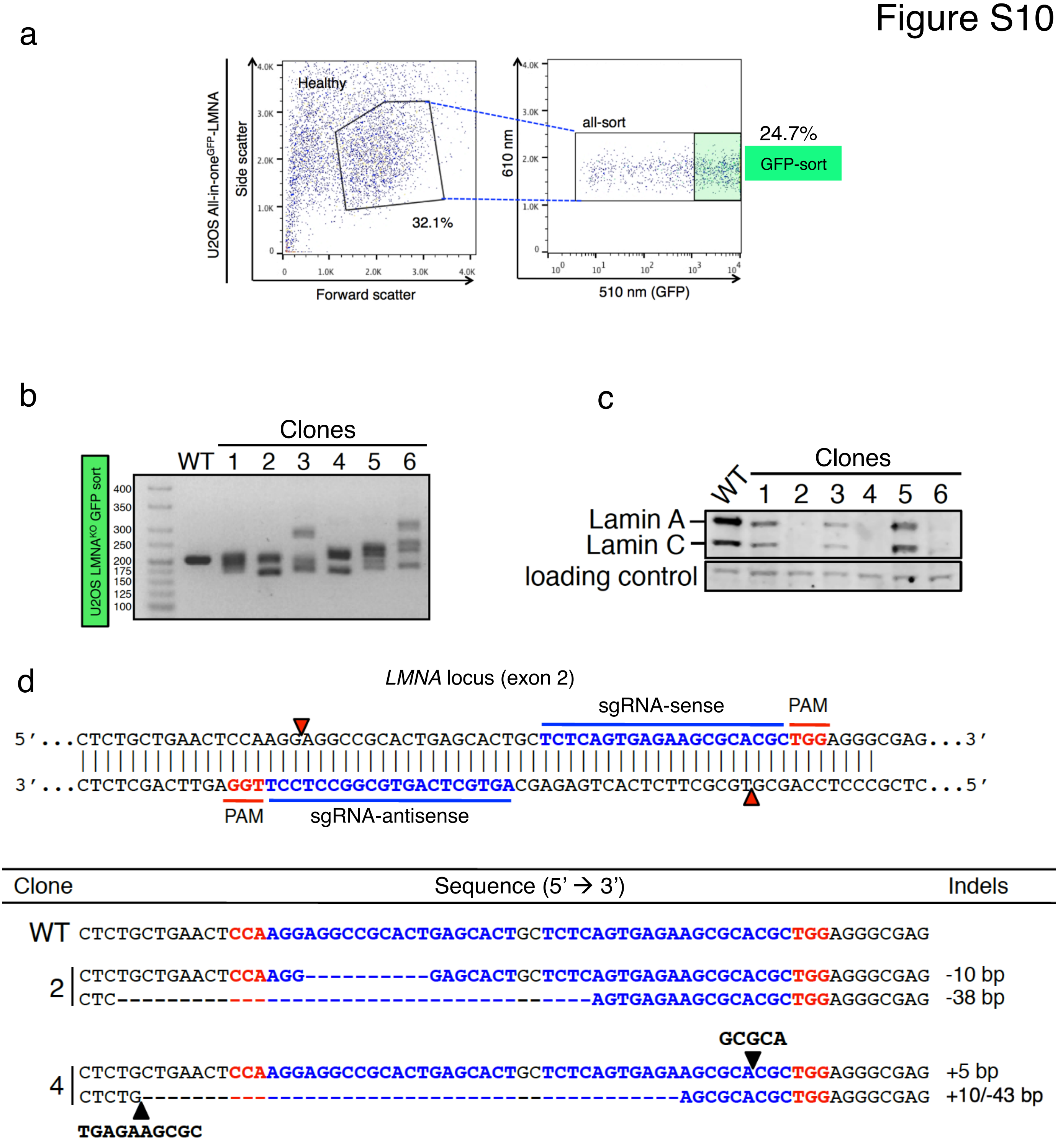

Supplementary Figure 10 *LMNA* knockout in U2OS cells.

(**a**) FACS data showing the sequential gating of healthy (all-sort, left panel) and high-EGFP expressing (GFP-sort, right panel) cell populations. High-EGFP expressing cells were sorted into 96-well plates at a single-cell-per-well density. (**b**) PCR-genotyping of GFP-sort clones. WT product size is 207 bp. (**c**) Western blots of LMNA knock-out candidates. (**d**) On-target sequence of the *LMNA* locus where PAM sequences are indicated in red and sgRNA target sequences in blue. Red arrowheads indicate the Cas9^{D10A} nicking sites. Each sequence represents one allele in the knock-out clones. Dashed lines indicate deletions; black arrowheads indicate the precise locations of insertions. Inserted sequences are tagged with black arrowheads. The exact sizes of indels for each allele are shown on the right.

a

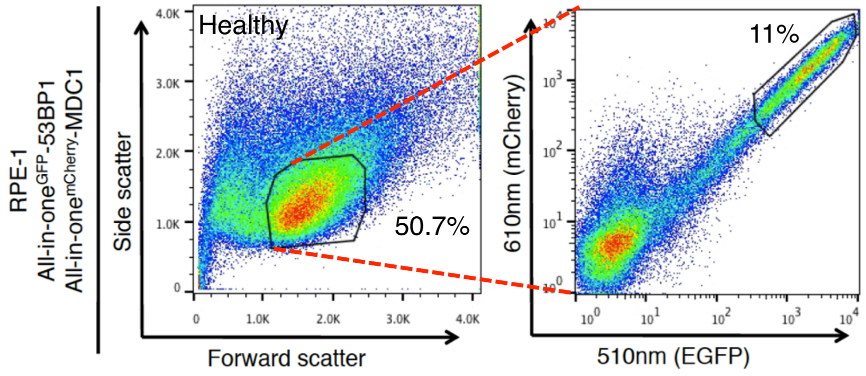

RPE-1
All-in-one GFP-53BP1
All-in-one mCherry-MDC1
Healthy
50.7%
Side scatter
Forward scatter
11%
610nm (mCherry)
510nm (EGFP)

b

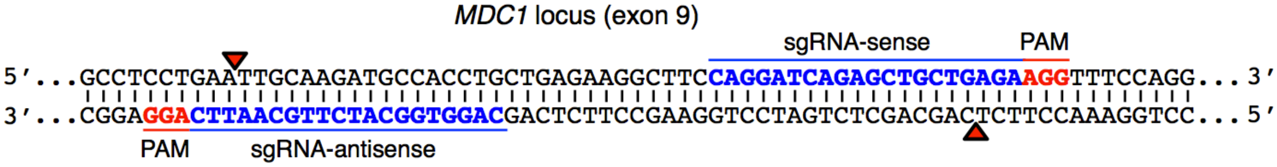

MDC1 locus (exon 9)
sgRNA-sense
PAM
5′...GCCTCCTGAATTGCAAGATGCCACCTGCTGAGAAGGCTTCCAGGATCAGAGCTGCTGAGAAGGTTTCCAGG...3′
3′...CGGAGGACTTAACGTTCTACGGTGGACGACTCTTCCGAAGGTCCTAGTCTCGACGACTCTTCCAAAGGTCC...5′
PAM
sgRNA-antisense

c

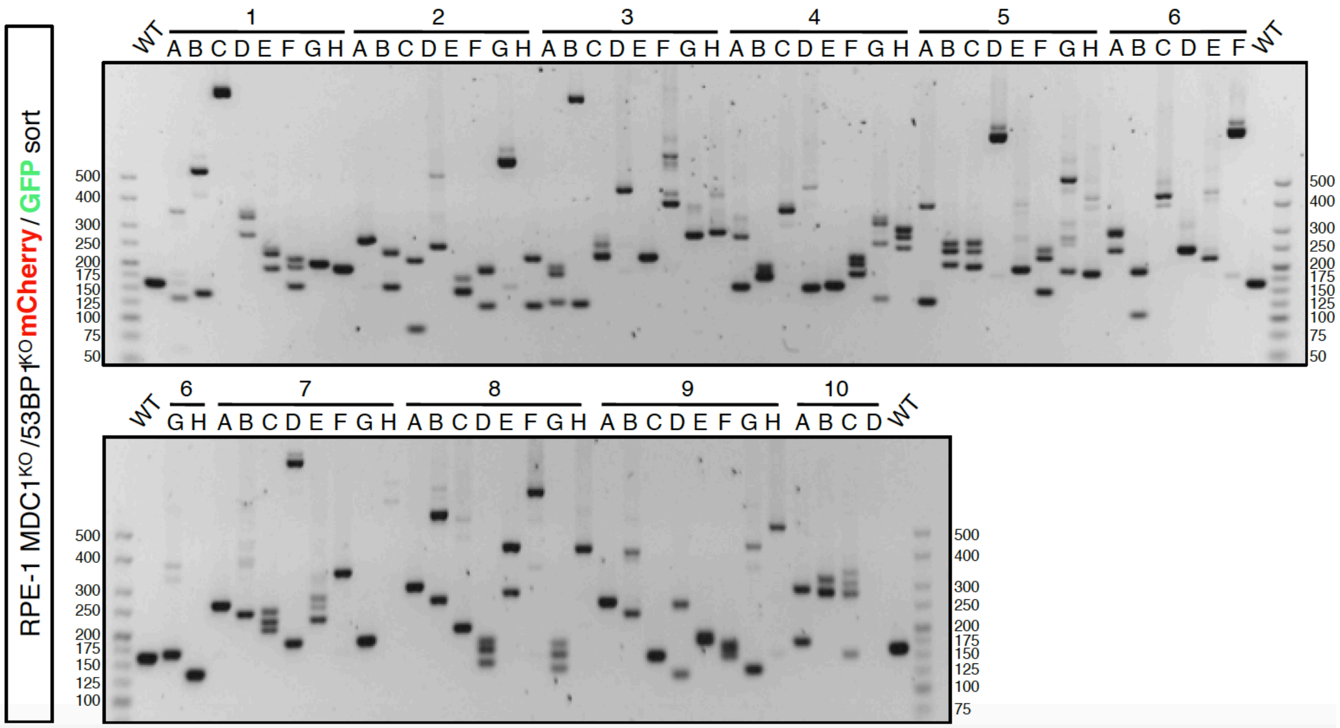

RPE-1 MDC1KO/53BP1KO mCherry/GFP sort
WT
1
2
3
4
5
6
7
8
9
10
500
400
300
250
200
175
150
125
100
75
50

d

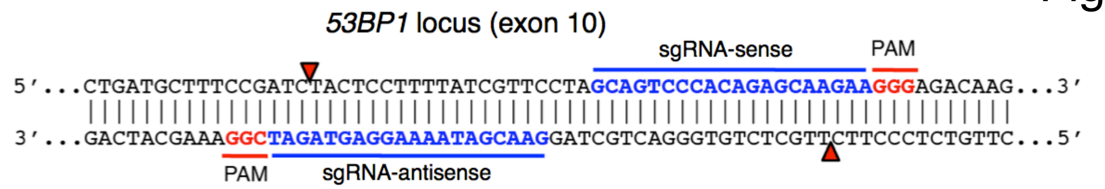

e

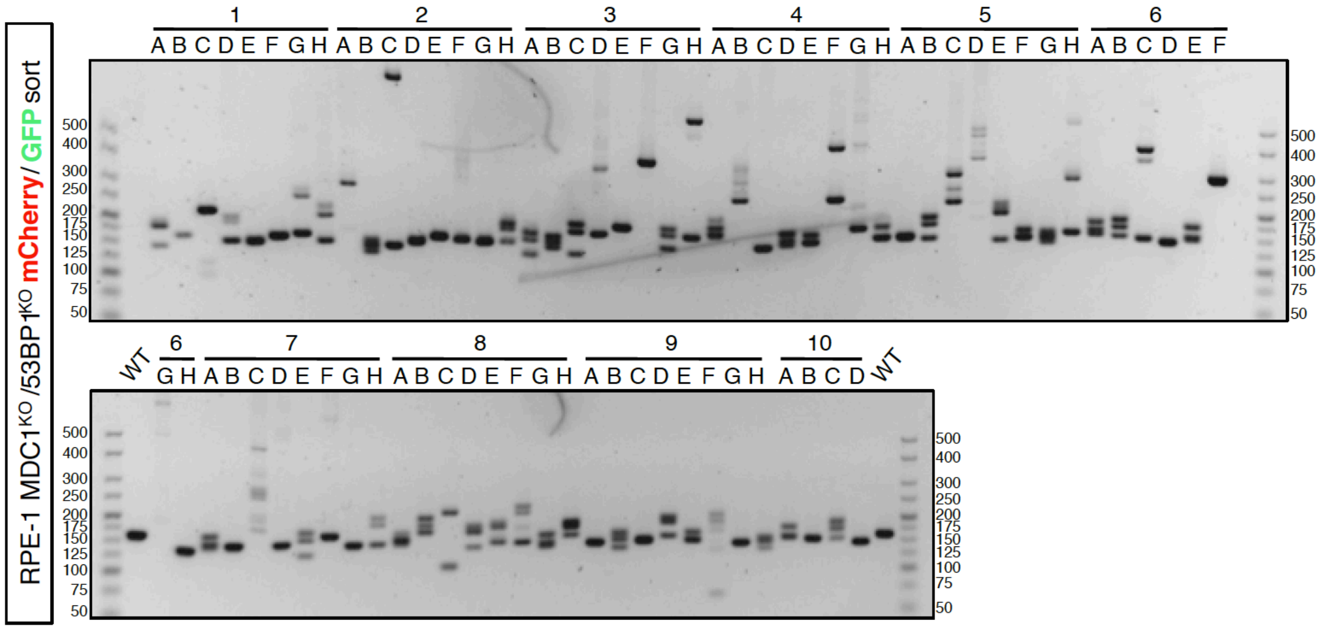

f

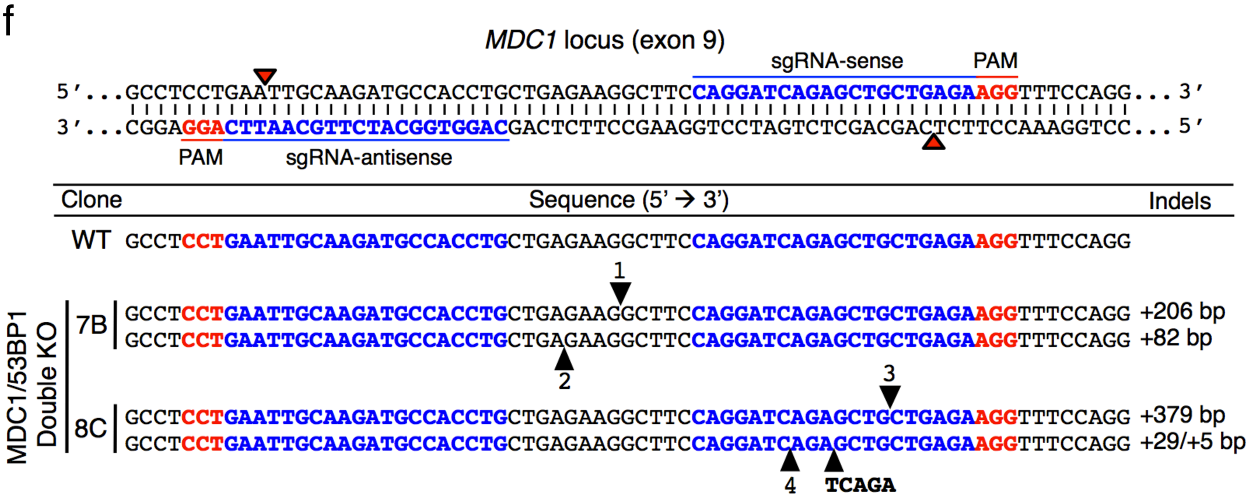

1:
GCTTCCTGCTGAGAAGGCTTCCTGAGAAGGCTTCCTGCTGAGAAGGCTTCCTGAGAAGGCTTCCTGCTGAGAAGGCTTCCTGAG
AAGGCTTCCTGCTGAGAAGGCTTCCAGGCTTCCTGAGAAGGCTTCCTGCTGAGAAGGCTTCCTGAGAAGGCTTCCTGCTGAGAA
GGCTTCCAGGCTTCCTGAGAAGGCTTCCTGCTGAGAAG

2:
GAAGGCTTCCTGCTGAGAAGGCTTCCTGAGAAGGCTTCCTGCTGAGAAGGCTTCCAGGCTTCCTGAGAAGGCTTCCTGCTGA

3:
AGAAGGCTTCCAGGATCAATGCCACCTGCTGAGAAGGCTTCCAGGATCAGAGCTGAGAAGGCTTCCAGGATCAGAGCTGATGCCA
CCTGCTGAGAAGGCTTCCAGGATCAGAGCTGAGAAGGCTTCCAGGATCAATGCCATTGCAAGATGCCACCTGCTGAGAAGGCTTC
CAGGATCAGAGCTGAGAAGGCTTCCAGGATCAATGCCACCTGCTGAGAAGGCTTCCAGGATCAGAGCTGAGAAGGCTTCCAGGAT
CAGAGCTGATGCCACCTGCTGAGAAGGCTTCCAGGATCAGAGCTGAGAAGGCTTCCAGGATCAATGCCACCTGCTGAGAAGGCTT
CCAGGATCAGAGCTGAGAAGGCTTCCAGGATCAGAGCTG

4:
TGCCACCTGCTGAGAAGGCTTCCAGGATC

Figure S11

Supplementary Figure 11 Simultaneous double knockout for MDC1 and 53BP1 in RPE-1 cells.

(a) FACS data showing the sequential gating of healthy (left panel) and high-mCherry/EGFP co-expressing (mCherry/EGFP sort, right panel, as shown in Fig. 5B) cell populations. The mCherry+EGFP population was sorted into 96-well plates at a single-cell-per-well density. (**b**) Schematic of *MDC1* sgRNA target sequences at the exon 9. (**c**) PCR-genotyping across the *MDC1* (exon 9) target locus of individual clones. WT product size is 164 bp. (**d**) Schematic of *53BP1* sgRNA target sequences at exon 10. (**e**) PCR-genotyping across the *53BP1* (exon 10) target locus of individual clones. WT product size is 162 bp. (**f**) The full sequences of insertions at the *MDC1* (exon 9) target site from MDC1/53BP1 double knockout clones 7B (1 and 2) and 8C (3 and 4) as shown in Figure 5F.

Figure S12

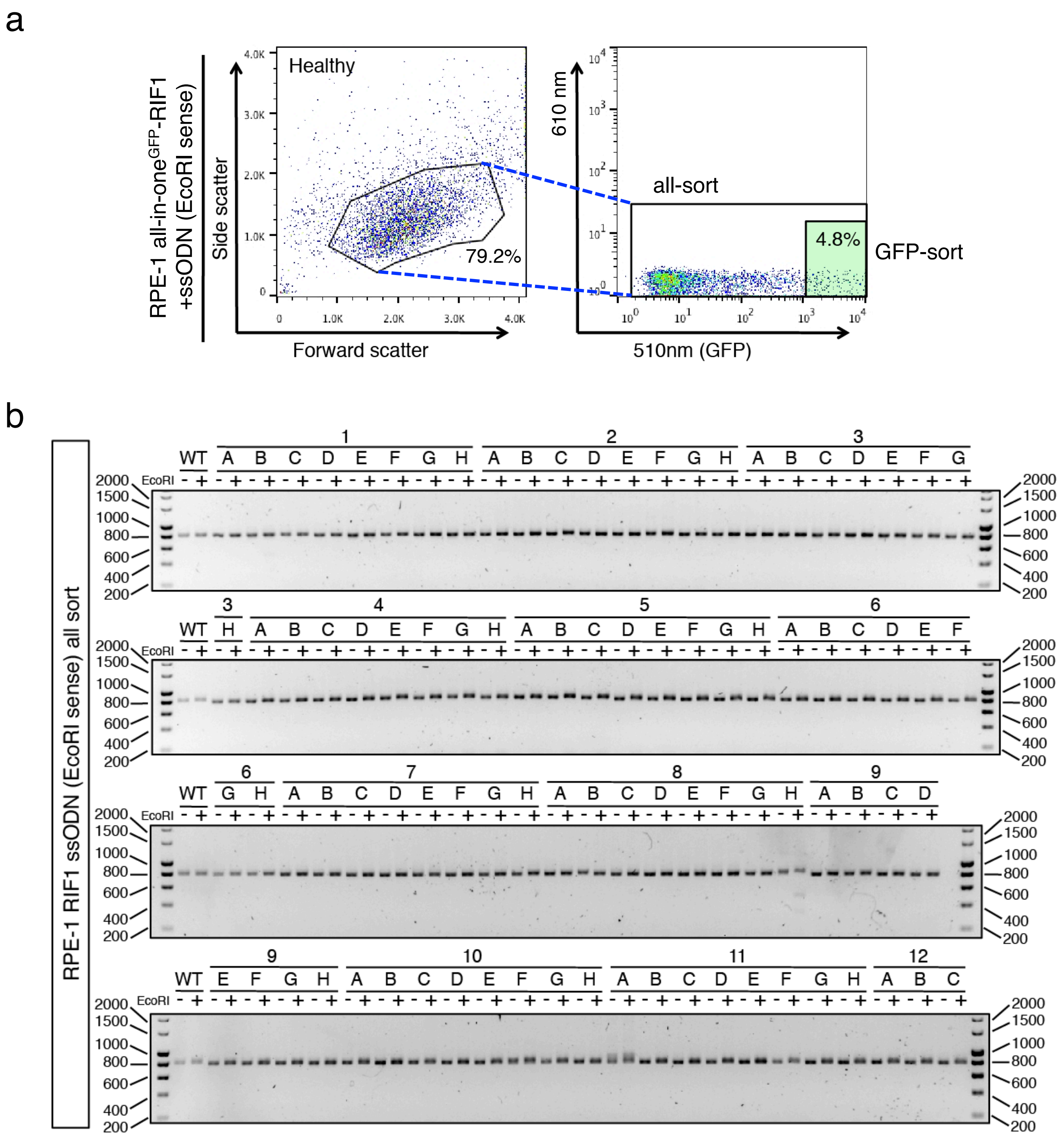
a
RPE-1 all-in-one^{GFP}-RIF1 +ssODN (EcoRI sense)
Healthy
79.2%
Side scatter
Forward scatter
610 nm
510nm (GFP)
all-sort
4.8%
GFP-sort
b
RPE-1 RIF1 ssODN (EcoRI sense) all sort
WT
EcoRI
2000
1500
1000
800
600
400
200

Figure S12

c

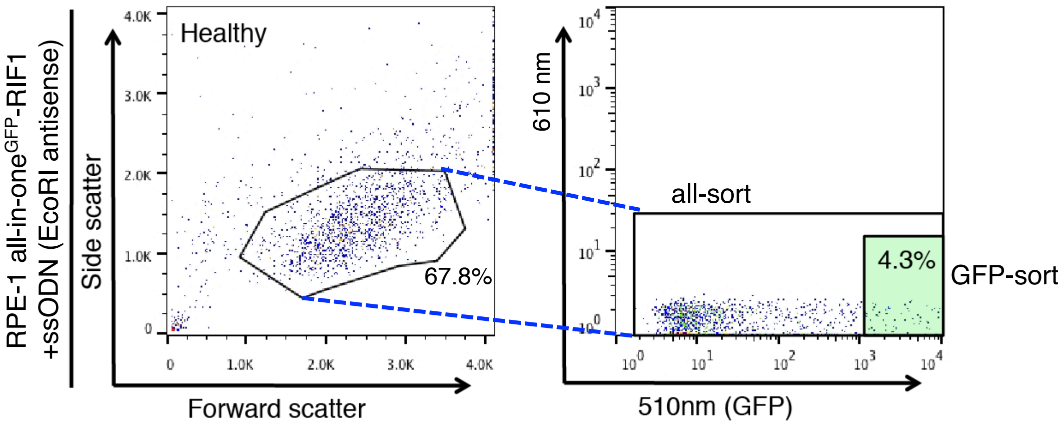

d

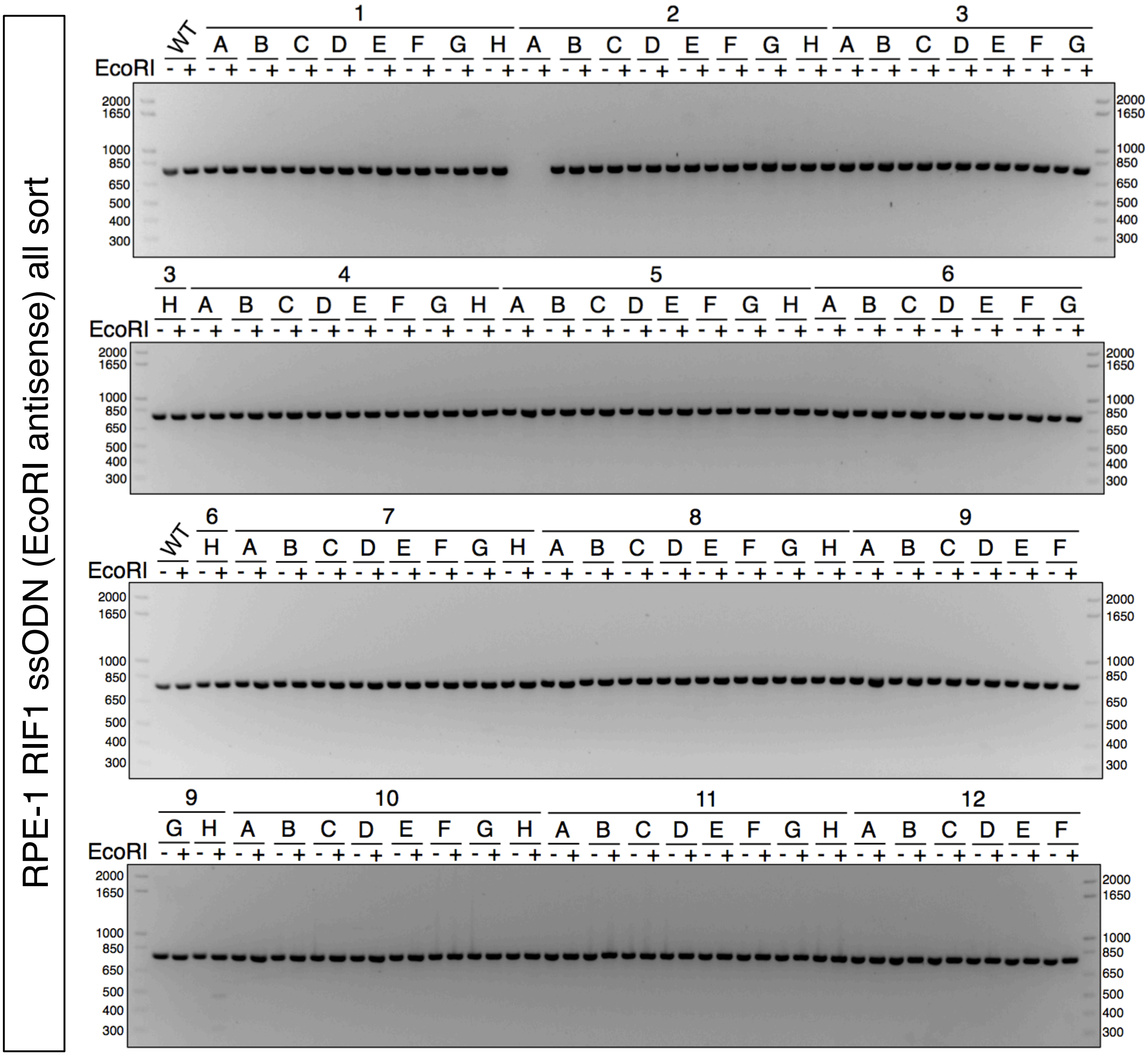

Figure S12

e

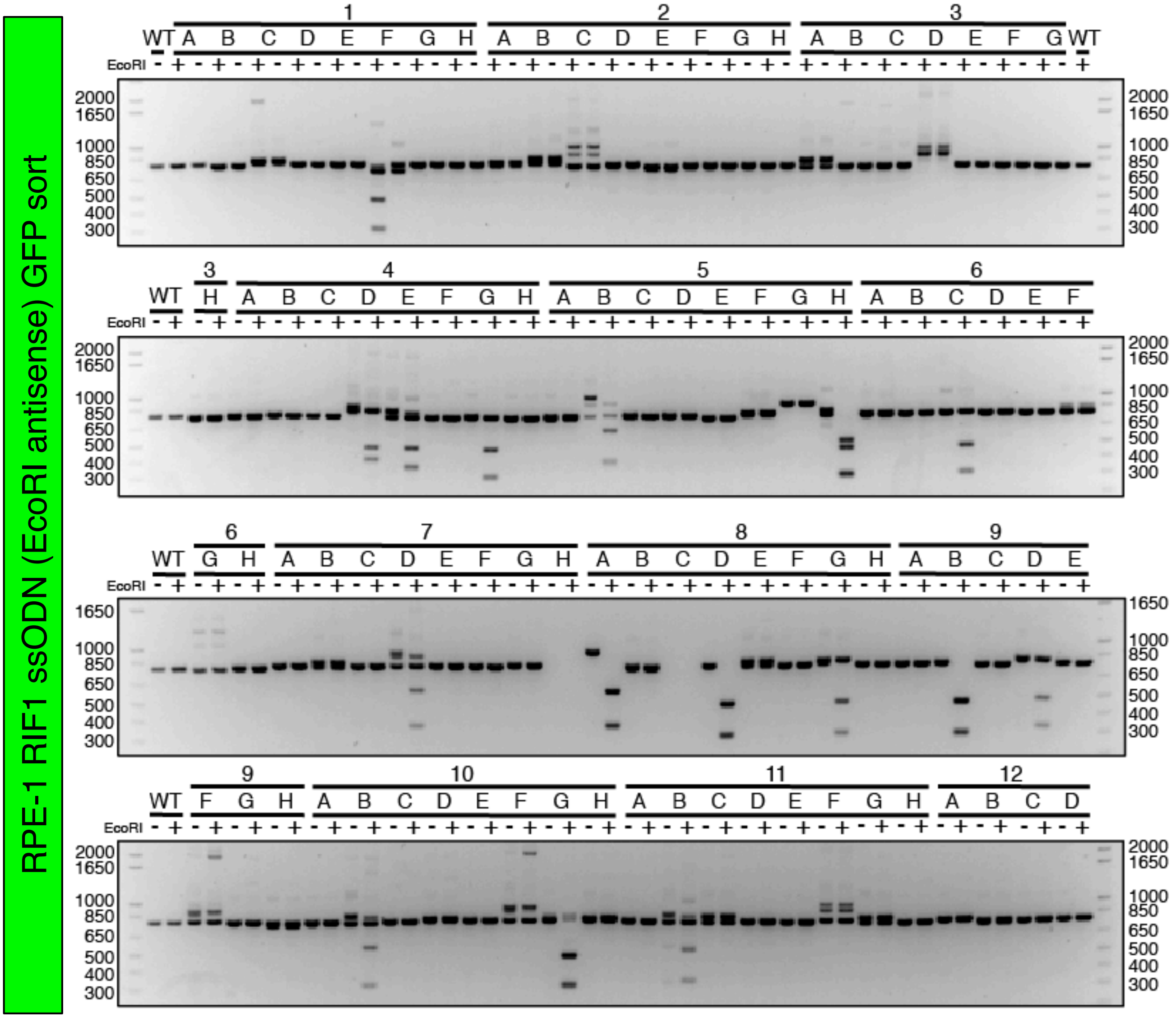

f

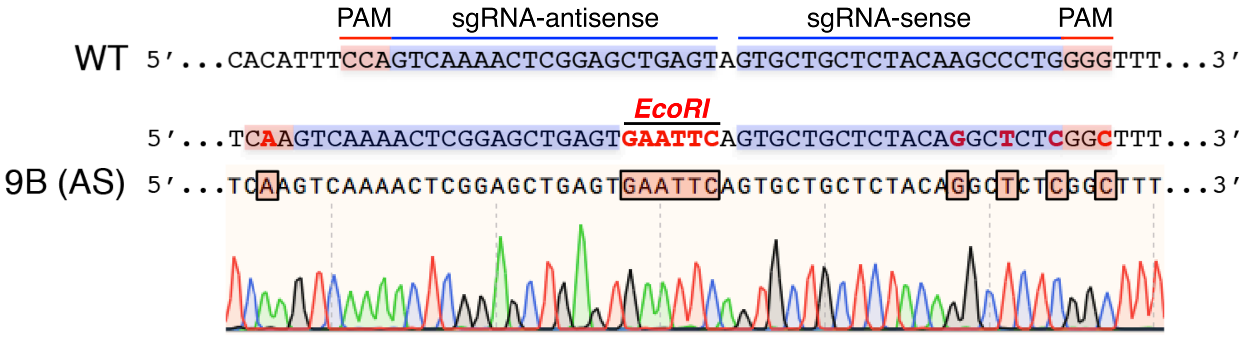

Figure S12

Supplementary Figure 12 *RIF1-EcoRI* site knock-in via HDR-mediated repair with sense or antisense ssODN.

(**a**) FACS data showing the sequential gating of healthy (all-sort) and high-EGFP expressing (GFP-sort) cell populations transfected with all-in-one Cas9^{D10A} nickase vector targeting the *RIF1* locus and sense strand ssODN. Each of these populations was sorted into 96-well plates at a single-cell-per-well density. (**b**) PCR-genotyping and *EcoRI* restriction digestion of individual clones from the sense ssODN all-sorted population. WT product size is 791 bp. (**c**) FACS data of cells transfected with all-in-one Cas9^{D10A} nickase vector targeting the *RIF1* locus and antisense strand ssODN. (**d**) PCR-genotyping and *EcoRI* restriction digestion of individual clones from the antisense ssODN all-sort population. WT product size is 791 bp. (**e**) PCR-genotyping and *EcoRI* restriction digestion of individual clones from the antisense ssODN GFP-sort population. Successful knock-in allele size is 797 bp; *EcoRI* digestion produced 487 bp and 310 bp. (**f**) Sequencing of knock-in clone 9B from (e). PAM sequences are highlighted in red and sgRNA sequences are in blue. *EcoRI* sequence and mutations at both PAM and sgRNA sequences are indicated in red. The sequencing result of homozygous knock-in clone 9B is aligned at the bottom. Mutated nucleotides are indicated in red boxes.

Figure S13

a

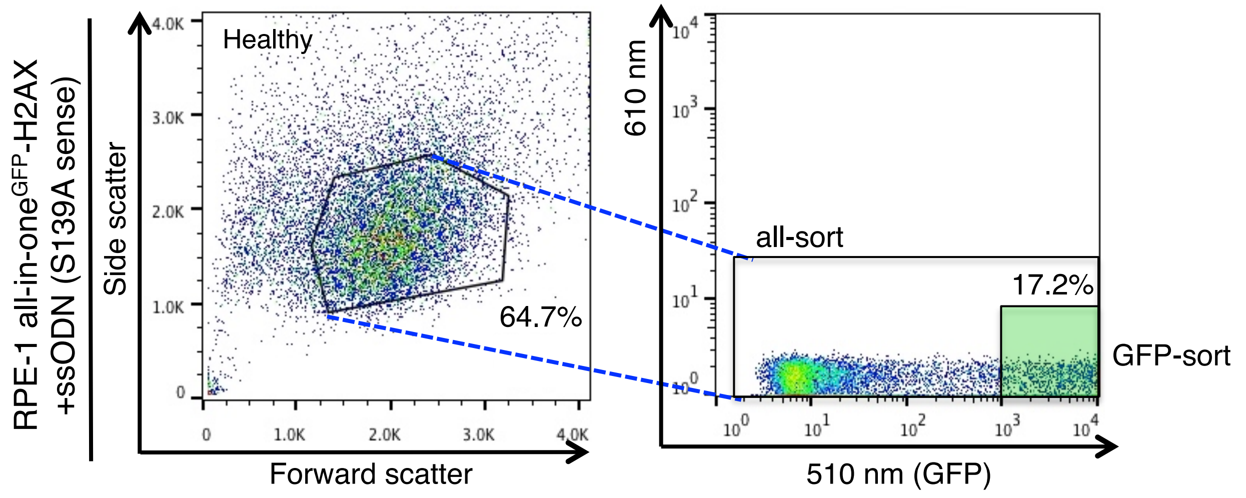

b

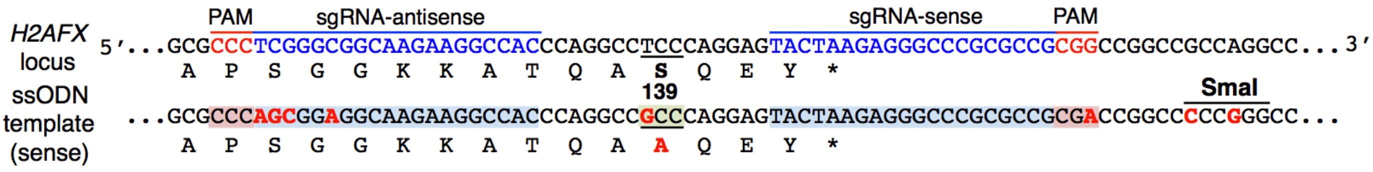

c

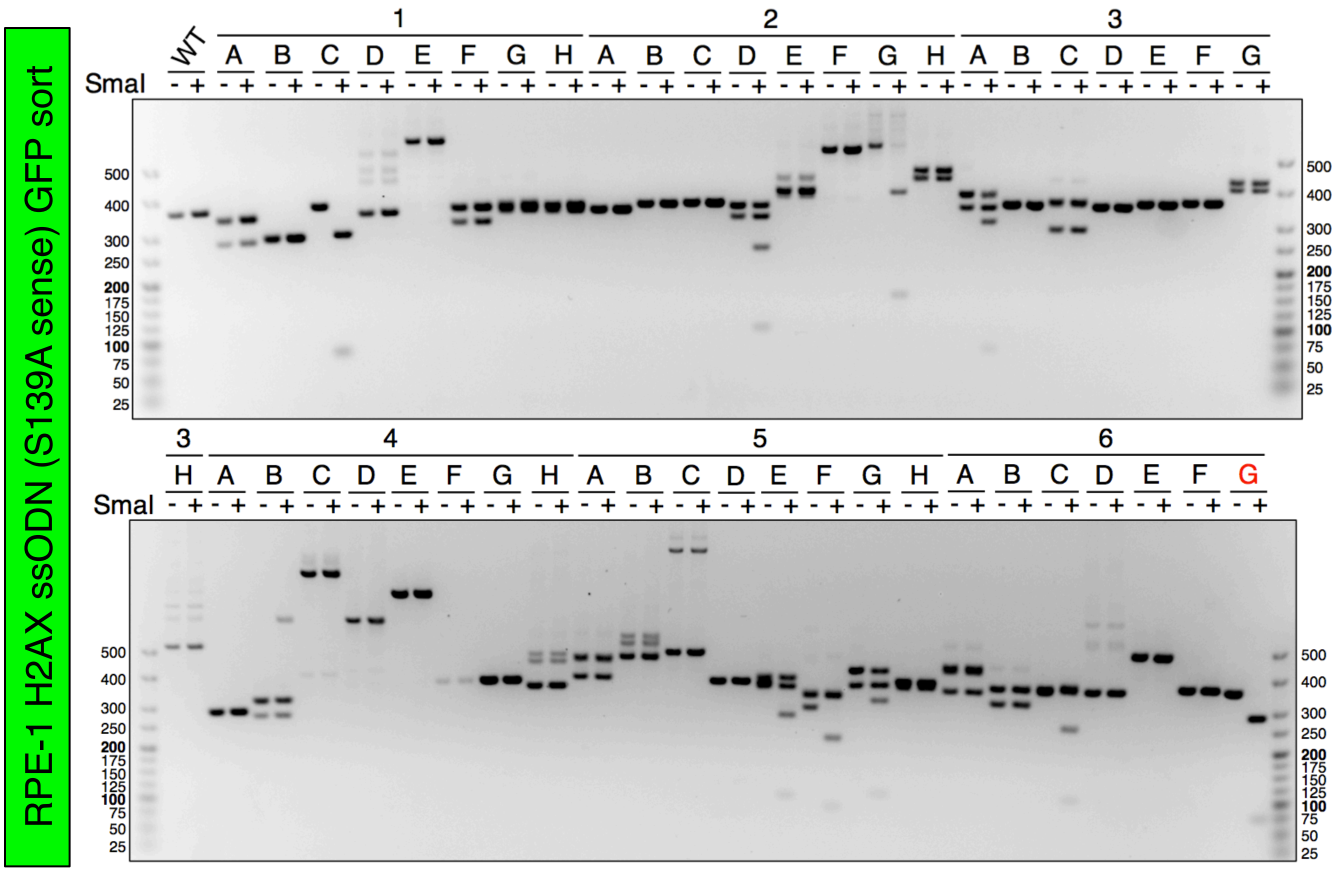

Figure S13

d

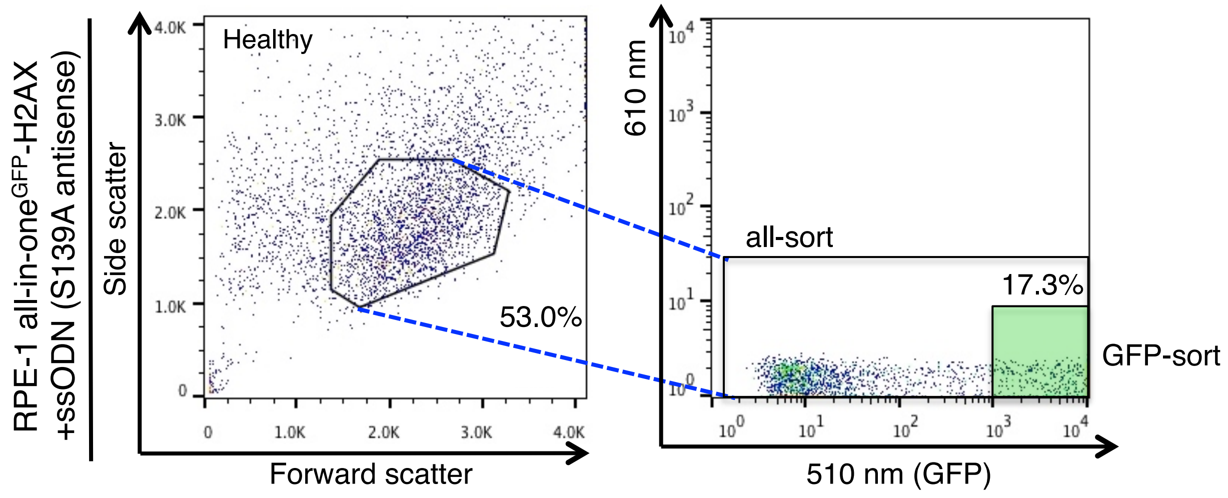

e

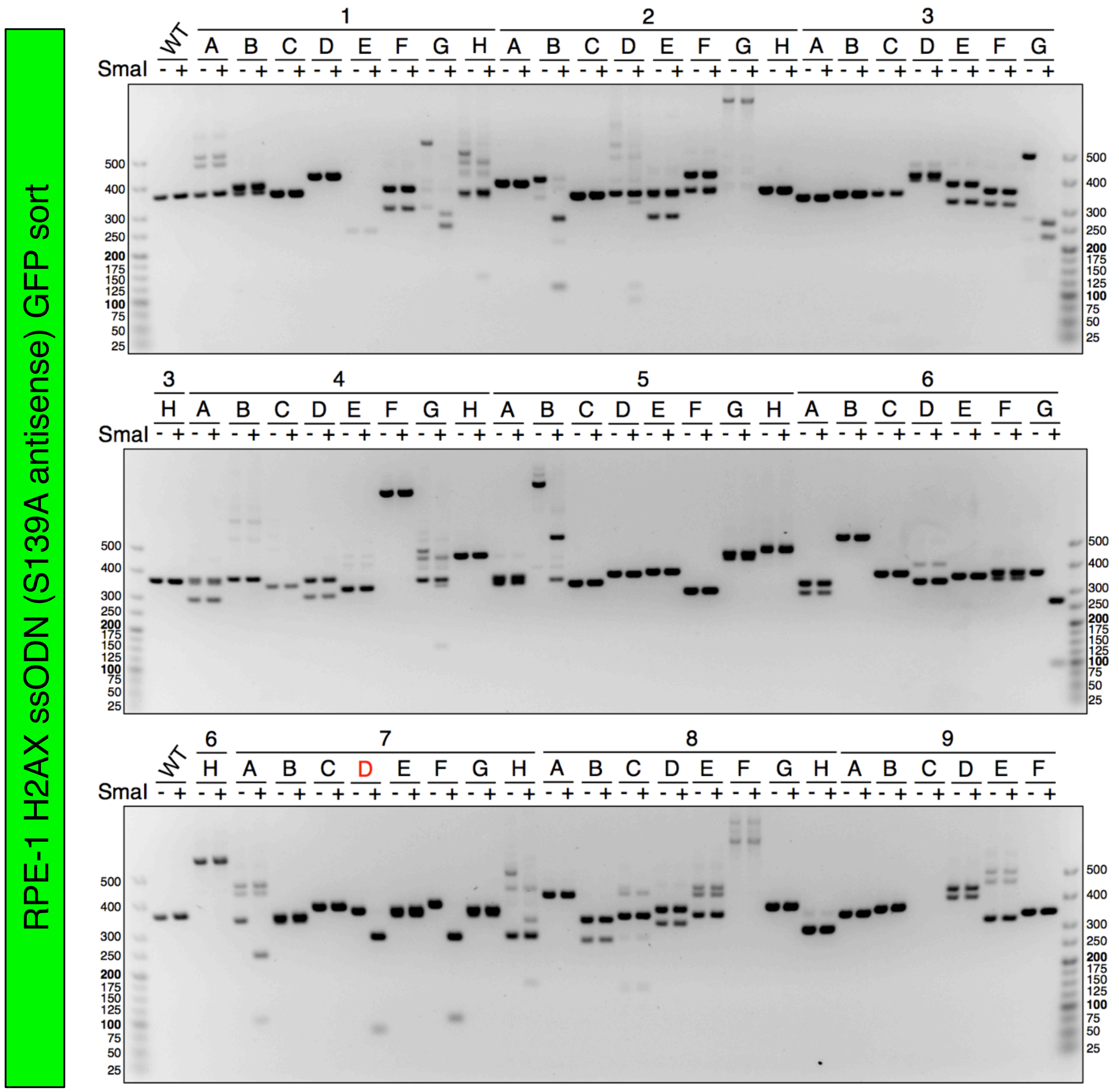

Supplementary Figure 13 H2AX^{S139A} point mutation knock-in via HR-mediated repair with ssODN.

(**a**) FACS data showing the sequential gating of healthy (all-sort) and high-EGFP expressing (GFP-sort) cell populations transfected with all-in-one Cas9^{D10A} nickase vector targeting the *H2AFX* locus and sense strand ssODN. Cells were sorted into 96-well plates at a single-cell-per-well density. (**b**) Schematic of knocking in H2AX^{S139A} point mutation and *SmaI* site for rapid genotypic screening as shown in Figure 7C. (**c**) PCR-genotyping and *SmaI* restriction digestion of individual clones from the sense ssODN GFP-sort population. WT product size is 363 bp; *SmaI* digestion produced 287 bp and 76 bp. (**d**) FACS data of cells transfected with all-in-one Cas9^{D10A} nickase vector targeting the *H2AFX* locus and antisense strand ssODN. (**e**) PCR-genotyping and *SmaI* restriction digestion of individual clones from the antisense ssODN GFP-sort population.

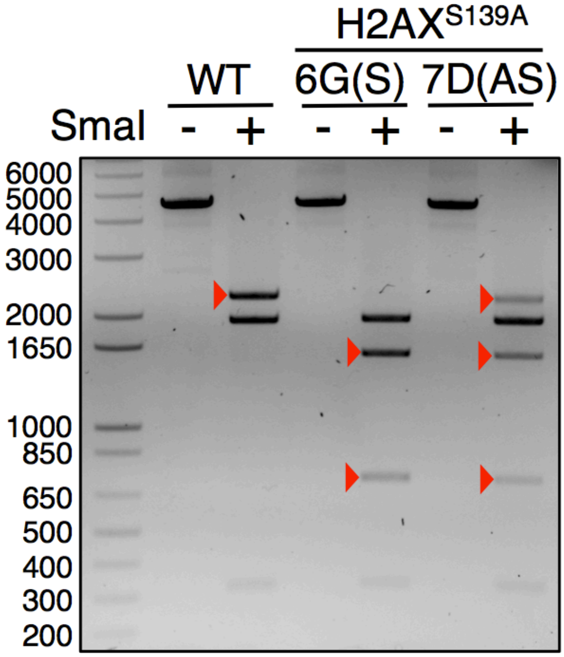

Supplementary Figure 14 Genotyping analysis of long PCR products across the *H2AFX* target locus by *SmaI* digestion. There are three intrinsic *SmaI* sites in this long PCR product of the wild-type allele where the undigested product length is 4571 bp and *SmaI*-digested products are 2258 bp, 1947 bp, 319 bp and 47 bp (off the gel). Successful knock-in allele after *SmaI* (four recognition sites) digestion showed bands of 1947 bp, 1553 bp, 705 bp, 319 bp and 47 bp (off the gel). Red triangles indicate the difference between the wild-type allele and the knock-in allele. Clone 6G (S): biallelic knock-in; 7D (AS): monoallelic knock-in with the other allele modified with a deletion of 76 bp at the target region (confirmed by DNA sequencing).

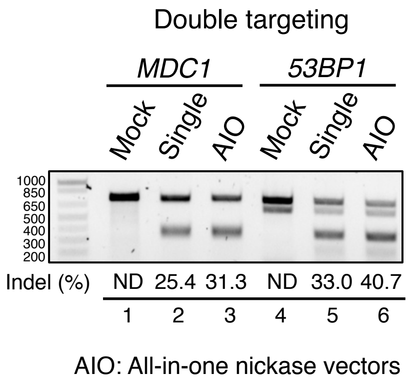

Supplementary Figure 15 Cas9^{D10A} nickase double-targeting mutagenic efficiencies by T7 EI assays in human HEK293FT cells. Cells were either transfected with or without (Mock, lanes 1 and 4) four single sgRNA nickase vectors each carrying Cas9^{D10A} nickase with either sense or antisense sgRNA targeting *MDC1* or *53BP1* locus (Single, lanes 2 and 5), or with two all-in-one dual sgRNA Cas9^{D10A} nickase vectors each targeting either *MDC1* or *53BP1* (AIO, lanes 3 and 6). Lanes 1-3 show PCR products across *MDC1* target locus while lanes 4-6 represent PCR products across *53BP1* locus. Indel frequencies are shown below. ND, not detected.

Supplementary Figure 16

The full sequences of the All-in-One-GFP/mCherry plasmids

AIO-GFP (9740 bp)

Colour code:

U6 promoter

Guide RNA+scaffold

$Cas9^{D10A}$

T2A ribosomal skipping peptide

EGFP

GAGGGCCTATTTCCCATGATTCCTTCATATTTGCATATACGATACAAGGCTGTTAGA
GAGATAATTGGAATTAATTTGACTGTAAACACAAAGATATTAGTACAAAATACGTGA
CGTAGAAAGTAATAATTTCTTGGGTAGTTTGCAGTTTTAAAATTATGTTTTAAAATG
GACTATCATATGCTTACCGTAACTTGAAAGTATTTCGATTTCTTGGCTTTATATATC
TTGTGGAAAGGACGAAACACCGGGGTCTTCGGATCCATGCGAAGACCTGTTTTAGAG
CTAGAAATAGCAAGTTAAAATAAGGCTAGTCCGTTATCAACTTGAAAAAGTGGCACC
GAGTCGGTGCTTTTTTGTTTTAGAGCTAGAAATAGCAAGTTAAAATAAGGCTAGTCC
GTTTTTAGCGCGTGCGCCAATTCTGCAGACAAATGGCTCTAGATTAACCCTCACTAA
AGGGAAAGGTCGGGCAGGAAGAGGGCCTATTTCCCATGATTCCTTCATATTTGCATA
TACGATACAAGGCTGTTAGAGAGATAATTAGAATTAATTTGACTGTAAACACAAAGA
TATTAGTACAAAATACGTGACGTAGAAAGTAATAATTTCTTGGGTAGTTTGCAGTTT
TAAAATTATGTTTTAAAATGGACTATCATATGCTTACCGTAACTTGAAAGTATTTCG
ATTTCTTGGCTTTATATATCTTGTGGAAAGGACGAAACACCGGGAGACCATCGATGA
GAGGGTCTCAGTTTTAGAGCTAGAAATAGCAAGTTAAAATAAGGCTAGTCCGTTATC
AACTTGAAAAAGTGGCACCGAGTCGGTGCTTTTTTTTTCTATAGTGTCACCTAAATT
GCAGCTCTGGCCCGTGTCTCAAAATCTCTGGTACCCGTTACATAACTTACGGTAAAT
GGCCCGCCTGGCTGACCGCCCAACGACCCCCGCCCATTGACGTCAATAGTAACGCCA
ATAGGGACTTTCCATTGACGTCAATGGGTGGAGTATTTACGGTAAACTGCCCACTTG
GCAGTACATCAAGTGTATCATATGCCAAGTACGCCCCCTATTGACGTCAATGACGGT
AAATGGCCCGCCTGGCATTGTGCCCAGTACATGACCTTATGGGACTTTCCTACTTGG
CAGTACATCTACGTATTAGTCATCGCTATTACCATGGTCGAGGTGAGCCCCACGTTC
TGCTTCACTCTCCCCATCTCCCCCCCCTCCCCACCCCCAATTTTGTATTTATTTATT
TTTTAATTATTTTGTGCAGCGATGGGGGCGGGGGGGGGGGGGGGGCGCGCGCCAGGC
GGGGCGGGGCGGGGCGAGGGGCGGGGCGGGGCGAGGCGGAGAGGTGCGGCGGCAGCC
AATCAGAGCGGCGCGCTCCGAAAGTTTCCTTTTATGGCGAGGCGGCGGCGGCGGCGG
CCCTATAAAAAGCGAAGCGCGCGGCGGGCGGGAGTCGCTGCGACGCTGCCTTCGCCC
CGTGCCCCGCTCCGCCGCCGCCTCGCGCCGCCCGCCCCGGCTCTGACTGACCGCGTT
ACTCCCACAGGTGAGCGGGCGGGACGGCCCTTCTCCTCCGGGCTGTAATTAGCTGAG
CAAGAGGTAAGGGTTTAAGGGATGGTTGGTTGGTGGGGTATTAATGTTTAATTACCT
GGAGCACCTGCCTGAAATCACTTTTTTTCAGGTTGGACCGGTGCCACCATGGACTAT
AAGGACCACGACGGAGACTACAAGGATCATGATATTGATTACAAAGACGATGACGAT
AAGATGGCCCCAAAGAAGAAGCGGAAGGTCGGTATCCACGGAGTCCCAGCAGCCGAC
AAGAAGTACAGCATCGGCCTGGCCATCGGCACCAACTCTGTGGGCTGGGCCGTGATC

ACCGACGAGTACAAGGTGCCCAGCAAGAAATTCAAGGTGCTGGGCAACACCGACCGG
CACAGCATCAAGAAGAACCTGATCGGAGCCCTGCTGTTCGACAGCGGCGAAACAGCC
GAGGCCACCCGGCTGAAGAGAACCGCCAGAAGAAGATACACCAGACGGAAGAACCGG
ATCTGCTATCTGCAAGAGATCTTCAGCAACGAGATGGCCAAGGTGGACGACAGCTTC
TTCCACAGACTGGAAGAGTCCTTCCTGGTGGAAGAGGATAAGAAGCACGAGCGGCAC
CCCATCTTCGGCAACATCGTGGACGAGGTGGCCTACCACGAGAAGTACCCCACCATC
TACCACCTGAGAAAGAAACTGGTGGACAGCACCGACAAGGCCGACCTGCGGCTGATC
TATCTGGCCCTGGCCCACATGATCAAGTTCCGGGGCCACTTCCTGATCGAGGGCGAC
CTGAACCCCGACAACAGCGACGTGGACAAGCTGTTCATCCAGCTGGTGCAGACCTAC
AACCAGCTGTTCGAGGAAAACCCCATCAACGCCAGCGGCGTGGACGCCAAGGCCATC
CTGTCTGCCAGACTGAGCAAGAGCAGACGGCTGGAAAATCTGATCGCCCAGCTGCCC
GGCGAGAAGAAGAATGGCCTGTTCGGCAACCTGATTGCCCTGAGCCTGGGCCTGACC
CCCAACTTCAAGAGCAACTTCGACCTGGCCGAGGATGCCAAACTGCAGCTGAGCAAG
GACACCTACGACGACGACCTGGACAACCTGCTGGCCCAGATCGGCGACCAGTACGCC
GACCTGTTTCTGGCCGCCAAGAACCTGTCCGACGCCATCCTGCTGAGCGACATCCTG
AGAGTGAACACCGAGATCACCAAGGCCCCCCTGAGCGCCTCTATGATCAAGAGATAC
GACGAGCACCACCAGGACCTGACCCTGCTGAAAGCTCTCGTGCGGCAGCAGCTGCCT
GAGAAGTACAAAGAGATTTTCTTCGACCAGAGCAAGAACGGCTACGCCGGCTACATT
GACGGCGGAGCCAGCCAGGAAGAGTTCTACAAGTTCATCAAGCCCATCCTGGAAAAG
ATGGACGGCACCGAGGAACTGCTCGTGAAGCTGAACAGAGAGGACCTGCTGCGGAAG
CAGCGGACCTTCGACAACGGCAGCATCCCCCACCAGATCCACCTGGGAGAGCTGCAC
GCCATTCTGCGGCGGCAGGAAGATTTTTACCCATTCCTGAAGGACAACCGGGAAAAG
ATCGAGAAGATCCTGACCTTCCGCATCCCCTACTACGTGGGCCCTCTGGCCAGGGGA
AACAGCAGATTCGCCTGGATGACCAGAAAGAGCGAGGAAACCATCACCCCCTGGAAC
TTCGAGGAAGTGGTGGACAAGGGCGCTTCCGCCCAGAGCTTCATCGAGCGGATGACC
AACTTCGATAAGAACCTGCCCAACGAGAAGGTGCTGCCCAAGCACAGCCTGCTGTAC
GAGTACTTCACCGTGTATAACGAGCTGACCAAAGTGAAATACGTGACCGAGGGAATG
AGAAAGCCCGCCTTCCTGAGCGGCGAGCAGAAAAAGGCCATCGTGGACCTGCTGTTC
AAGACCAACCGGAAAGTGACCGTGAAGCAGCTGAAAGAGGACTACTTCAAGAAAATC
GAGTGCTTCGACTCCGTGGAAATCTCCGGCGTGGAAGATCGGTTCAACGCCTCCCTG
GGCACATACCACGATCTGCTGAAAATTATCAAGGACAAGGACTTCCTGGACAATGAG
GAAAACGAGGACATTCTGGAAGATATCGTGCTGACCCTGACACTGTTTGAGGACAGA
GAGATGATCGAGGAACGGCTGAAAACCTATGCCCACCTGTTCGACGACAAAGTGATG
AAGCAGCTGAAGCGGCGGAGATACACCGGCTGGGGCAGGCTGAGCCGGAAGCTGATC
AACGGCATCCGGGACAAGCAGTCCGGCAAGACAATCCTGGATTTCCTGAAGTCCGAC
GGCTTCGCCAACAGAAACTTCATGCAGCTGATCCACGACGACAGCCTGACCTTTAAA
GAGGACATCCAGAAAGCCCAGGTGTCCGGCCAGGGCGATAGCCTGCACGAGCACATT
GCCAATCTGGCCGGCAGCCCCGCCATTAAGAAGGGCATCCTGCAGACAGTGAAGGTG
GTGGACGAGCTCGTGAAAGTGATGGGCCGGCACAAGCCCGAGAACATCGTGATCGAA
ATGGCCAGAGAGAACCAGACCACCCAGAAGGGACAGAAGAACAGCCGCGAGAGAATG
AAGCGGATCGAAGAGGGCATCAAAGAGCTGGGCAGCCAGATCCTGAAAGAACACCCC
GTGGAAAACACCCAGCTGCAGAACGAGAAGCTGTACCTGTACTACCTGCAGAATGGG
CGGGATATGTACGTGGACCAGGAACTGGACATCAACCGGCTGTCCGACTACGATGTG
GACCATATCGTGCCTCAGAGCTTTCTGAAGGACGACTCCATCGACAACAAGGTGCTG
ACCAGAAGCGACAAGAACCGGGGCAAGAGCGACAACGTGCCCTCCGAAGAGGTCGTG
AAGAAGATGAAGAACTACTGGCGGCAGCTGCTGAACGCCAAGCTGATTACCCAGAGA
AAGTTCGACAATCTGACCAAGGCCGAGAGAGGCGGCCTGAGCGAACTGGATAAGGCC
GGCTTCATCAAGAGACAGCTGGTGGAAACCCGGCAGATCACAAAGCACGTGGCACAG
ATCCTGGACTCCCGGATGAACACTAAGTACGACGAGAATGACAAGCTGATCCGGGAA
GTGAAAGTGATCACCCTGAAGTCCAAGCTGGTGTCCGATTTCCGGAAGGATTTCCAG
TTTTACAAAGTGCGCGAGATCAACAACTACCACCACGCCCACGACGCCTACCTGAAC
GCCGTCGTGGGAACCGCCCTGATCAAAAAGTACCCTAAGCTGGAAAGCGAGTTCGTG

TACGGCGACTACAAGGTGTACGACGTGCGGAAGATGATCGCCAAGAGCGAGCAGGAA
ATCGGCAAGGCTACCGCCAAGTACTTCTTCTACAGCAACATCATGAACTTTTTCAAG
ACCGAGATTACCCTGGCCAACGGCGAGATCCGGAAGCGGCCTCTGATCGAGACAAAC
GGCGAAACCGGGGAGATCGTGTGGGATAAGGGCCGGGATTTTGCCACCGTGCGGAAA
GTGCTGAGCATGCCCCAAGTGAATATCGTGAAAAAGACCGAGGTGCAGACAGGCGGC
TTCAGCAAAGAGTCTATCCTGCCCAAGAGGAACAGCGATAAGCTGATCGCCAGAAAG
AAGGACTGGGACCCTAAGAAGTACGGCGGCTTCGACAGCCCCACCGTGGCCTATTCT
GTGCTGGTGGTGGCCAAAGTGGAAAAGGGCAAGTCCAAGAAACTGAAGAGTGTGAAA
GAGCTGCTGGGGATCACCATCATGGAAAGAAGCAGCTTCGAGAAGAATCCCATCGAC
TTTCTGGAAGCCAAGGGCTACAAAGAAGTGAAAAAGGACCTGATCATCAAGCTGCCT
AAGTACTCCCTGTTCGAGCTGGAAAACGGCCGGAAGAGAATGCTGGCCTCTGCCGGC
GAACTGCAGAAGGGAAACGAACTGGCCCTGCCCTCCAAATATGTGAACTTCCTGTAC
CTGGCCAGCCACTATGAGAAGCTGAAGGGCTCCCCCGAGGATAATGAGCAGAAACAG
CTGTTTGTGGAACAGCACAAGCACTACCTGGACGAGATCATCGAGCAGATCAGCGAG
TTCTCCAAGAGAGTGATCCTGGCCGACGCTAATCTGGACAAAGTGCTGTCCGCCTAC
AACAAGCACCGGGATAAGCCCATCAGAGAGCAGGCCGAGAATATCATCCACCTGTTT
ACCCTGACCAATCTGGGAGCCCCTGCCGCCTTCAAGTACTTTGACACCACCATCGAC
CGGAAGAGGTACACCAGCACCAAAGAGGTGCTGGACGCCACCCTGATCCACCAGAGC
ATCACCGGCCTGTACGAGACACGGATCGACCTGTCTCAGCTGGGAGGCGACAAAAGG
CCGGCGGCCACGAAAAAGGCCGGCCAGGCAAAAAAGAAAAAGGAATTCGGCAGTGGA
GAGGGCAGAGGAAGTCTGCTAACATGCGGTGACGTCGAGGAGAATCCTGGCCCAGTG
AGCAAGGGCGAGGAGCTGTTCACCGGGGTGGTGCCCATCCTGGTCGAGCTGGACGGC
GACGTAAACGGCCACAAGTTCAGCGTGTCCGGCGAGGGCGAGGGCGATGCCACCTAC
GGCAAGCTGACCCTGAAGTTCATCTGCACCACCGGCAAGCTGCCCGTGCCCTGGCCC
ACCCTCGTGACCACCCTGACCTACGGCGTGCAGTGCTTCAGCCGCTACCCCGACCAC
ATGAAGCAGCACGACTTCTTCAAGTCCGCCATGCCCGAAGGCTACGTCCAGGAGCGC
ACCATCTTCTTCAAGGACGACGGCAACTACAAGACCCGCGCCGAGGTGAAGTTCGAG
GGCGACACCCTGGTGAACCGCATCGAGCTGAAGGGCATCGACTTCAAGGAGGACGGC
AACATCCTGGGGCACAAGCTGGAGTACAACTACAACAGCCACAACGTCTATATCATG
GCCGACAAGCAGAAGAACGGCATCAAGGTGAACTTCAAGATCCGCCACAACATCGAG
GACGGCAGCGTGCAGCTCGCCGACCACTACCAGCAGAACACCCCCATCGGCGACGGC
CCCGTGCTGCTGCCCGACAACCACTACCTGAGCACCCAGTCCGCCCTGAGCAAAGAC
CCCAACGAGAAGCGCGATCACATGGTCCTGCTGGAGTTCGTGACCGCCGCCGGGATC
ACTCTCGGCATGGACGAGCTGTACAAGGAATTCTAACTAGAGCTCGCTGATCAGCCT
CGACTGTGCCTTCTAGTTGCCAGCCATCTGTTGTTTGCCCCTCCCCCGTGCCTTCCT
TGACCCTGGAAGGTGCCACTCCCACTGTCCTTTCCTAATAAAATGAGGAAATTGCAT
CGCATTGTCTGAGTAGGTGTCATTCTATTCTGGGGGGTGGGGTGGGGCAGGACAGCA
AGGGGGAGGATTGGGAAGAGAATAGCAGGCATGCTGGGGAGCGGCCGCAGGAACCCC
TAGTGATGGAGTTGGCCACTCCCTCTCTGCGCGCTCGCTCGCTCACTGAGGCCGGGC
GACCAAAGGTCGCCCGACGCCCGGGCTTTGCCCGGGCGGCCTCAGTGAGCGAGCGAG
CGCGCAGCTGCCTGCAGGGGCGCCTGATGCGGTATTTTCTCCTTACGCATCTGTGCG
GTATTTCACACCGCATACGTCAAAGCAACCATAGTACGCGCCCTGTAGCGGCGCATT
AAGCGCGGCGGGTGTGGTGGTTACGCGCAGCGTGACCGCTACACTTGCCAGCGCCCT
AGCGCCCGCTCCTTTCGCTTTCTTCCCTTCCTTTCTCGCCACGTTCGCCGGCTTTCC
CCGTCAAGCTCTAAATCGGGGGCTCCCTTTAGGGTTCCGATTTAGTGCTTTACGGCA
CCTCGACCCCAAAAAACTTGATTTGGGTGATGGTTCACGTAGTGGGCCATCGCCCTG
ATAGACGGTTTTTCGCCCTTTGACGTTGGAGTCCACGTTCTTTAATAGTGGACTCTT
GTTCCAAACTGGAACAACACTCAACCCTATCTCGGGCTATTCTTTTGATTTATAAGG
GATTTTGCCGATTTCGGCCTATTGGTTAAAAAATGAGCTGATTTAACAAAAATTTAA
CGCGAATTTTAACAAAATATTAACGTTTACAATTTTATGGTGCACTCTCAGTACAAT
CTGCTCTGATGCCGCATAGTTAAGCCAGCCCCGACACCCGCCAACACCCGCTGACGC
GCCCTGACGGGCTTGTCTGCTCCCGGCATCCGCTTACAGACAAGCTGTGACCGTCTC

CGGGAGCTGCATGTGTCAGAGGTTTTCACCGTCATCACCGAAACGCGCGAGACGAAA
GGGCCTCGTGATACGCCTATTTTTATAGGTTAATGTCATGATAATAATGGTTTCTTA
GACGTCAGGTGGCACTTTTCGGGGAAATGTGCGCGGAACCCCTATTTGTTTATTTTT
CTAAATACATTCAAATATGTATCCGCTCATGAGACAATAACCCTGATAAATGCTTCA
ATAATATTGAAAAAGGAAGAGTATGAGTATTCAACATTTCCGTGTCGCCCTTATTCC
CTTTTTTGCGGCATTTTGCCTTCCTGTTTTTGCTCACCCAGAAACGCTGGTGAAAGT
AAAAGATGCTGAAGATCAGTTGGGTGCACGAGTGGGTTACATCGAACTGGATCTCAA
CAGCGGTAAGATCCTTGAGAGTTTTCGCCCCGAAGAACGTTTTCCAATGATGAGCAC
TTTTAAAGTTCTGCTATGTGGCGCGGTATTATCCCGTATTGACGCCGGGCAAGAGCA
ACTCGGTCGCCGCATACACTATTCTCAGAATGACTTGGTTGAGTACTCACCAGTCAC
AGAAAAGCATCTTACGGATGGCATGACAGTAAGAGAATTATGCAGTGCTGCCATAAC
CATGAGTGATAACACTGCGGCCAACTTACTTCTGACAACGATCGGAGGACCGAAGGA
GCTAACCGCTTTTTTGCACAACATGGGGGATCATGTAACTCGCCTTGATCGTTGGGA
ACCGGAGCTGAATGAAGCCATACCAAACGACGAGCGTGACACCACGATGCCTGTAGC
AATGGCAACAACGTTGCGCAAACTATTAACTGGCGAACTACTTACTCTAGCTTCCCG
GCAACAATTAATAGACTGGATGGAGGCGGATAAAGTTGCAGGACCACTTCTGCGCTC
GGCCCTTCCGGCTGGCTGGTTTATTGCTGATAAATCTGGAGCCGGTGAGCGTGGAAG
CCGCGGTATCATTGCAGCACTGGGGCCAGATGGTAAGCCCTCCCGTATCGTAGTTAT
CTACACGACGGGGAGTCAGGCAACTATGGATGAACGAAATAGACAGATCGCTGAGAT
AGGTGCCTCACTGATTAAGCATTGGTAACTGTCAGACCAAGTTTACTCATATATACT
TTAGATTGATTTAAAACTTCATTTTTAATTTAAAAGGATCTAGGTGAAGATCCTTTT
TGATAATCTCATGACCAAAATCCCTTAACGTGAGTTTTCGTTCCACTGAGCGTCAGA
CCCCGTAGAAAAGATCAAAGGATCTTCTTGAGATCCTTTTTTTCTGCGCGTAATCTG
CTGCTTGCAAACAAAAAAACCACCGCTACCAGCGGTGGTTTGTTTGCCGGATCAAGA
GCTACCAACTCTTTTTCCGAAGGTAACTGGCTTCAGCAGAGCGCAGATACCAAATAC
TGTCCTTCTAGTGTAGCCGTAGTTAGGCCACCACTTCAAGAACTCTGTAGCACCGCC
TACATACCTCGCTCTGCTAATCCTGTTACCAGTGGCTGCTGCCAGTGGCGATAAGTC
GTGTCTTACCGGGTTGGACTCAAGACGATAGTTACCGGATAAGGCGCAGCGGTCGGG
CTGAACGGGGGGTTCGTGCACACAGCCCAGCTTGGAGCGAACGACCTACACCGAACT
GAGATACCTACAGCGTGAGCTATGAGAAAGCGCCACGCTTCCCGAAGGGAGAAAGGC
GGACAGGTATCCGGTAAGCGGCAGGGTCGGAACAGGAGAGCGCACGAGGGAGCTTCC
AGGGGGAAACGCCTGGTATCTTTATAGTCCTGTCGGGTTTCGCCACCTCTGACTTGA
GCGTCGATTTTTGTGATGCTCGTCAGGGGGGCGGAGCCTATGGAAAAACGCCAGCAA
CGCGGCCTTTTTACGGTTCCTGGCCTTTTGCTGGCCTTTTGCTCACATGT

AIO-mCherry (9728 bp)

Colour code:

U6 promoter

Guide RNA+scaffold

Cas9^{D10A}

P2A ribosomal skipping peptide

mCherry

GAGGGCCTATTTCCCATGATTCCTTCATATTTGCATATACGATACAAGGCTGTTAGA
GAGATAATTGGAATTAATTTGACTGTAAACACAAAGATATTAGTACAAAATACGTGA
CGTAGAAAGTAATAATTTCTTGGGTAGTTTGCAGTTTTAAAATTATGTTTTAAAATG
GACTATCATATGCTTACCGTAACTTGAAAGTATTTCGATTTCTTGGCTTTATATATC
TTGTGGAAAGGACGAAACACCGGGGTCTTCGGATCCATGCGAAGACCTGTTTTAGAG
CTAGAAATAGCAAGTTAAAATAAGGCTAGTCCGTTATCAACTTGAAAAAGTGGCACC
GAGTCGGTGCTTTTTTGTTTTAGAGCTAGAAATAGCAAGTTAAAATAAGGCTAGTCC
GTTTTTAGCGCGTGCGCCAATTCTGCAGACAAATGGCTCTAGATTAACCCTCACTAA
AGGGAAAGGTCGGGCAGGAAGAGGGCCTATTTCCCATGATTCCTTCATATTTGCATA
TACGATACAAGGCTGTTAGAGAGATAATTAGAATTAATTTGACTGTAAACACAAAGA
TATTAGTACAAAATACGTGACGTAGAAAGTAATAATTTCTTGGGTAGTTTGCAGTTT
TAAAATTATGTTTTAAAATGGACTATCATATGCTTACCGTAACTTGAAAGTATTTCG
ATTTCTTGGCTTTATATATCTTGTGGAAAGGACGAAACACCGGGAGACCATCGATGA
GAGGGTCTCAGTTTTAGAGCTAGAAATAGCAAGTTAAAATAAGGCTAGTCCGTTATC
AACTTGAAAAAGTGGCACCGAGTCGGTGCTTTTTTTTTCTATAGTGTCACCTAAATT
GCAGCTCTGGCCCGTGTCTCAAAATCTCTGGTACCCGTTACATAACTTACGGTAAAT
GGCCCGCCTGGCTGACCGCCCAACGACCCCCGCCCATTGACGTCAATAGTAACGCCA
ATAGGGACTTTCCATTGACGTCAATGGGTGGAGTATTTACGGTAAACTGCCCACTTG
GCAGTACATCAAGTGTATCATATGCCAAGTACGCCCCCTATTGACGTCAATGACGGT
AAATGGCCCGCCTGGCATTGTGCCCAGTACATGACCTTATGGGACTTTCCTACTTGG
CAGTACATCTACGTATTAGTCATCGCTATTACCATGGTCGAGGTGAGCCCCACGTTC
TGCTTCACTCTCCCCATCTCCCCCCCCTCCCCACCCCCAATTTTGTATTTATTTATT
TTTTAATTATTTTGTGCAGCGATGGGGGCGGGGGGGGGGGGGGGGCGCGCGCCAGGC
GGGGCGGGGCGGGGCGAGGGGCGGGGCGGGGCGAGGCGGAGAGGTGCGGCGGCAGCC
AATCAGAGCGGCGCGCTCCGAAAGTTTCCTTTTATGGCGAGGCGGCGGCGGCGGCGG
CCCTATAAAAAGCGAAGCGCGCGGCGGGCGGGAGTCGCTGCGACGCTGCCTTCGCCC
CGTGCCCCGCTCCGCCGCCGCCTCGCGCCGCCCGCCCCGGCTCTGACTGACCGCGTT
ACTCCCACAGGTGAGCGGGCGGGACGGCCCTTCTCCTCCGGGCTGTAATTAGCTGAG
CAAGAGGTAAGGGTTTAAGGGATGGTTGGTTGGTGGGGTATTAATGTTTAATTACCT
GGAGCACCTGCCTGAAATCACTTTTTTTCAGGTTGGACCGGTGCCACCATGGACTAT
AAGGACCACGACGGAGACTACAAGGATCATGATATTGATTACAAAGACGATGACGAT
AAGATGGCCCCAAAGAAGAAGCGGAAGGTCGGTATCCACGGAGTCCCAGCAGCCGAC
AAGAAGTACAGCATCGGCCTGGCCATCGGCACCAACTCTGTGGGCTGGGCCGTGATC
ACCGACGAGTACAAGGTGCCCAGCAAGAAATTCAAGGTGCTGGGCAACACCGACCGG
CACAGCATCAAGAAGAACCTGATCGGAGCCCTGCTGTTCGACAGCGGCGAAACAGCC
GAGGCCACCCGGCTGAAGAGAACCGCCAGAAGAAGATACACCAGACGGAAGAACCGG
ATCTGCTATCTGCAAGAGATCTTCAGCAACGAGATGGCCAAGGTGGACGACAGCTTC
TTCCACAGACTGGAAGAGTCCTTCCTGGTGGAAGAGGATAAGAAGCACGAGCGGCAC

CCCATCTTCGGCAACATCGTGGACGAGGTGGCCTACCACGAGAAGTACCCCACCATC
TACCACCTGAGAAAGAAACTGGTGGACAGCACCGACAAGGCCGACCTGCGGCTGATC
TATCTGGCCCTGGCCCACATGATCAAGTTCCGGGGCCACTTCCTGATCGAGGGCGAC
CTGAACCCCGACAACAGCGACGTGGACAAGCTGTTCATCCAGCTGGTGCAGACCTAC
AACCAGCTGTTCGAGGAAAACCCCATCAACGCCAGCGGCGTGGACGCCAAGGCCATC
CTGTCTGCCAGACTGAGCAAGAGCAGACGGCTGGAAAATCTGATCGCCCAGCTGCCC
GGCGAGAAGAAGAATGGCCTGTTCGGCAACCTGATTGCCCTGAGCCTGGGCCTGACC
CCCAACTTCAAGAGCAACTTCGACCTGGCCGAGGATGCCAAACTGCAGCTGAGCAAG
GACACCTACGACGACGACCTGGACAACCTGCTGGCCCAGATCGGCGACCAGTACGCC
GACCTGTTTCTGGCCGCCAAGAACCTGTCCGACGCCATCCTGCTGAGCGACATCCTG
AGAGTGAACACCGAGATCACCAAGGCCCCCCTGAGCGCCTCTATGATCAAGAGATAC
GACGAGCACCACCAGGACCTGACCCTGCTGAAAGCTCTCGTGCGGCAGCAGCTGCCT
GAGAAGTACAAAGAGATTTTCTTCGACCAGAGCAAGAACGGCTACGCCGGCTACATT
GACGGCGGAGCCAGCCAGGAAGAGTTCTACAAGTTCATCAAGCCCATCCTGGAAAAG
ATGGACGGCACCGAGGAACTGCTCGTGAAGCTGAACAGAGAGGACCTGCTGCGGAAG
CAGCGGACCTTCGACAACGGCAGCATCCCCCACCAGATCCACCTGGGAGAGCTGCAC
GCCATTCTGCGGCGGCAGGAAGATTTTTACCCATTCCTGAAGGACAACCGGGAAAAG
ATCGAGAAGATCCTGACCTTCCGCATCCCCTACTACGTGGGCCCTCTGGCCAGGGGA
AACAGCAGATTCGCCTGGATGACCAGAAAGAGCGAGGAAACCATCACCCCCTGGAAC
TTCGAGGAAGTGGTGGACAAGGGCGCTTCCGCCCAGAGCTTCATCGAGCGGATGACC
AACTTCGATAAGAACCTGCCCAACGAGAAGGTGCTGCCCAAGCACAGCCTGCTGTAC
GAGTACTTCACCGTGTATAACGAGCTGACCAAAGTGAAATACGTGACCGAGGGAATG
AGAAAGCCCGCCTTCCTGAGCGGCGAGCAGAAAAAGGCCATCGTGGACCTGCTGTTC
AAGACCAACCGGAAAGTGACCGTGAAGCAGCTGAAAGAGGACTACTTCAAGAAAATC
GAGTGCTTCGACTCCGTGGAAATCTCCGGCGTGGAAGATCGGTTCAACGCCTCCCTG
GGCACATACCACGATCTGCTGAAAATTATCAAGGACAAGGACTTCCTGGACAATGAG
GAAAACGAGGACATTCTGGAAGATATCGTGCTGACCCTGACACTGTTTGAGGACAGA
GAGATGATCGAGGAACGGCTGAAAACCTATGCCCACCTGTTCGACGACAAAGTGATG
AAGCAGCTGAAGCGGCGGAGATACACCGGCTGGGGCAGGCTGAGCCGGAAGCTGATC
AACGGCATCCGGGACAAGCAGTCCGGCAAGACAATCCTGGATTTCCTGAAGTCCGAC
GGCTTCGCCAACAGAAACTTCATGCAGCTGATCCACGACGACAGCCTGACCTTTAAA
GAGGACATCCAGAAAGCCCAGGTGTCCGGCCAGGGCGATAGCCTGCACGAGCACATT
GCCAATCTGGCCGGCAGCCCCGCCATTAAGAAGGGCATCCTGCAGACAGTGAAGGTG
GTGGACGAGCTCGTGAAAGTGATGGGCCGGCACAAGCCCGAGAACATCGTGATCGAA
ATGGCCAGAGAGAACCAGACCACCCAGAAGGGACAGAAGAACAGCCGCGAGAGAATG
AAGCGGATCGAAGAGGGCATCAAAGAGCTGGGCAGCCAGATCCTGAAAGAACACCCC
GTGGAAAACACCCAGCTGCAGAACGAGAAGCTGTACCTGTACTACCTGCAGAATGGG
CGGGATATGTACGTGGACCAGGAACTGGACATCAACCGGCTGTCCGACTACGATGTG
GACCATATCGTGCCTCAGAGCTTTCTGAAGGACGACTCCATCGACAACAAGGTGCTG
ACCAGAAGCGACAAGAACCGGGGCAAGAGCGACAACGTGCCCTCCGAAGAGGTCGTG
AAGAAGATGAAGAACTACTGGCGGCAGCTGCTGAACGCCAAGCTGATTACCCAGAGA
AAGTTCGACAATCTGACCAAGGCCGAGAGAGGCGGCCTGAGCGAACTGGATAAGGCC
GGCTTCATCAAGAGACAGCTGGTGGAAACCCGGCAGATCACAAAGCACGTGGCACAG
ATCCTGGACTCCCGGATGAACACTAAGTACGACGAGAATGACAAGCTGATCCGGGAA
GTGAAAGTGATCACCCTGAAGTCCAAGCTGGTGTCCGATTTCCGGAAGGATTTCCAG
TTTTACAAAGTGCGCGAGATCAACAACTACCACCACGCCCACGACGCCTACCTGAAC
GCCGTCGTGGGAACCGCCCTGATCAAAAAGTACCCTAAGCTGGAAAGCGAGTTCGTG
TACGGCGACTACAAGGTGTACGACGTGCGGAAGATGATCGCCAAGAGCGAGCAGGAA
ATCGGCAAGGCTACCGCCAAGTACTTCTTCTACAGCAACATCATGAACTTTTTCAAG
ACCGAGATTACCCTGGCCAACGGCGAGATCCGGAAGCGGCCTCTGATCGAGACAAAC
GGCGAAACCGGGGAGATCGTGTGGGATAAGGGCCGGGATTTTGCCACCGTGCGGAAA
GTGCTGAGCATGCCCCAAGTGAATATCGTGAAAAAGACCGAGGTGCAGACAGGCGGC

TTCAGCAAAGAGTCTATCCTGCCCAAGAGGAACAGCGATAAGCTGATCGCCAGAAAG
AAGGACTGGGACCCTAAGAAGTACGGCGGCTTCGACAGCCCCACCGTGGCCTATTCT
GTGCTGGTGGTGGCCAAAGTGGAAAAGGGCAAGTCCAAGAAACTGAAGAGTGTGAAA
GAGCTGCTGGGGATCACCATCATGGAAAGAAGCAGCTTCGAGAAGAATCCCATCGAC
TTTCTGGAAGCCAAGGGCTACAAAGAAGTGAAAAAGGACCTGATCATCAAGCTGCCT
AAGTACTCCCTGTTCGAGCTGGAAAACGGCCGGAAGAGAATGCTGGCCTCTGCCGGC
GAACTGCAGAAGGGAAACGAACTGGCCCTGCCCTCCAAATATGTGAACTTCCTGTAC
CTGGCCAGCCACTATGAGAAGCTGAAGGGCTCCCCCGAGGATAATGAGCAGAAACAG
CTGTTTGTGGAACAGCACAAGCACTACCTGGACGAGATCATCGAGCAGATCAGCGAG
TTCTCCAAGAGAGTGATCCTGGCCGACGCTAATCTGGACAAAGTGCTGTCCGCCTAC
AACAAGCACCGGGATAAGCCCATCAGAGAGCAGGCCGAGAATATCATCCACCTGTTT
ACCCTGACCAATCTGGGAGCCCCTGCCGCCTTCAAGTACTTTGACACCACCATCGAC
CGGAAGAGGTACACCAGCACCAAAGAGGTGCTGGACGCCACCCTGATCCACCAGAGC
ATCACCGGCCTGTACGAGACACGGATCGACCTGTCTCAGCTGGGAGGCGACAAAAGG
CCGGCGGCCACGAAAAAGGCCGGCCAGGCAAAAAAGAAAAAGGGCTCCGGAGCCACG
AACTTCTCTCTGTTAAAGCAAGCAGGAGACGTGGAAGAAAACCCCGGTCCTGTGAGC
AAGGGCGAGGAGGATAACATGGCCATCATCAAGGAGTTCATGCGCTTCAAGGTGCAC
ATGGAGGGCTCCGTGAACGGCCACGAGTTCGAGATCGAGGGCGAGGGCGAGGGCCGC
CCCTACGAGGGCACCCAGACCGCCAAGCTGAAGGTGACCAAGGGTGGCCCCCTGCCC
TTCGCCTGGGACATCCTGTCCCCTCAGTTCATGTACGGCTCCAAGGCCTACGTGAAG
CACCCCGCCGACATCCCCGACTACTTGAAGCTGTCCTTCCCCGAGGGCTTCAAGTGG
GAGCGCGTGATGAACTTCGAGGACGGCGGCGTGGTGACCGTGACCCAGGACTCCTCC
CTGCAGGACGGCGAGTTCATCTACAAGGTGAAGCTGCGCGGCACCAACTTCCCCTCC
GACGGCCCCGTAATGCAGAAAAAGACCATGGGCTGGGAGGCCTCCTCCGAGCGGATG
TACCCCGAGGACGGCGCCCTGAAGGGCGAGATCAAGCAGAGGCTGAAGCTGAAGGAC
GGCGGCCACTACGACGCTGAGGTCAAGACCACCTACAAGGCCAAGAAGCCCGTGCAG
CTGCCCGGCGCCTACAACGTCAACATCAAGTTGGACATCACCTCCCACAACGAGGAC
TACACCATCGTGGAACAGTACGAACGCGCCGAGGGCCGCCACTCCACCGGCGGCATG
GACGAGCTGTACAAGGAATTCTAACTAGAGCTCGCTGATCAGCCTCGACTGTGCCTT
CTAGTTGCCAGCCATCTGTTGTTTGCCCCTCCCCCGTGCCTTCCTTGACCCTGGAAG
GTGCCACTCCCACTGTCCTTTCCTAATAAAATGAGGAAATTGCATCGCATTGTCTGA
GTAGGTGTCATTCTATTCTGGGGGGTGGGGTGGGGCAGGACAGCAAGGGGGAGGATT
GGGAAGAGAATAGCAGGCATGCTGGGGAGCGGCCGCAGGAACCCCTAGTGATGGAGT
TGGCCACTCCCTCTCTGCGCGCTCGCTCGCTCACTGAGGCCGGGCGACCAAAGGTCG
CCCGACGCCCGGGCTTTGCCCGGGCGGCCTCAGTGAGCGAGCGAGCGCGCAGCTGCC
TGCAGGGGCGCCTGATGCGGTATTTTCTCCTTACGCATCTGTGCGGTATTTCACACC
GCATACGTCAAAGCAACCATAGTACGCGCCCTGTAGCGGCGCATTAAGCGCGGCGGG
TGTGGTGGTTACGCGCAGCGTGACCGCTACACTTGCCAGCGCCCTAGCGCCCGCTCC
TTTCGCTTTCTTCCCTTCCTTTCTCGCCACGTTCGCCGGCTTTCCCCGTCAAGCTCT
AAATCGGGGGCTCCCTTTAGGGTTCCGATTTAGTGCTTTACGGCACCTCGACCCCAA
AAAACTTGATTTGGGTGATGGTTCACGTAGTGGGCCATCGCCCTGATAGACGGTTTT
TCGCCCTTTGACGTTGGAGTCCACGTTCTTTAATAGTGGACTCTTGTTCCAAACTGG
AACAACACTCAACCCTATCTCGGGCTATTCTTTTGATTTATAAGGGATTTTGCCGAT
TTCGGCCTATTGGTTAAAAAATGAGCTGATTTAACAAAAATTTAACGCGAATTTTAA
CAAAATATTAACGTTTACAATTTTATGGTGCACTCTCAGTACAATCTGCTCTGATGC
CGCATAGTTAAGCCAGCCCCGACACCCGCCAACACCCGCTGACGCGCCCTGACGGGC
TTGTCTGCTCCCGGCATCCGCTTACAGACAAGCTGTGACCGTCTCCGGGAGCTGCAT
GTGTCAGAGGTTTTCACCGTCATCACCGAAACGCGCGAGACGAAAGGGCCTCGTGAT
ACGCCTATTTTTATAGGTTAATGTCATGATAATAATGGTTTCTTAGACGTCAGGTGG
CACTTTTCGGGGAAATGTGCGCGGAACCCCTATTTGTTTATTTTTCTAAATACATTC
AAATATGTATCCGCTCATGAGACAATAACCCTGATAAATGCTTCAATAATATTGAAA
AAGGAAGAGTATGAGTATTCAACATTTCCGTGTCGCCCTTATTCCCTTTTTTGCGGC

ATTTTGCCTTCCTGTTTTTGCTCACCCAGAAACGCTGGTGAAAGTAAAAGATGCTGA
AGATCAGTTGGGTGCACGAGTGGGTTACATCGAACTGGATCTCAACAGCGGTAAGAT
CCTTGAGAGTTTTCGCCCCGAAGAACGTTTTCCAATGATGAGCACTTTTAAAGTTCT
GCTATGTGGCGCGGTATTATCCCGTATTGACGCCGGGCAAGAGCAACTCGGTCGCCG
CATACACTATTCTCAGAATGACTTGGTTGAGTACTCACCAGTCACAGAAAAGCATCT
TACGGATGGCATGACAGTAAGAGAATTATGCAGTGCTGCCATAACCATGAGTGATAA
CACTGCGGCCAACTTACTTCTGACAACGATCGGAGGACCGAAGGAGCTAACCGCTTT
TTTGCACAACATGGGGGATCATGTAACTCGCCTTGATCGTTGGGAACCGGAGCTGAA
TGAAGCCATACCAAACGACGAGCGTGACACCACGATGCCTGTAGCAATGGCAACAAC
GTTGCGCAAACTATTAACTGGCGAACTACTTACTCTAGCTTCCCGGCAACAATTAAT
AGACTGGATGGAGGCGGATAAAGTTGCAGGACCACTTCTGCGCTCGGCCCTTCCGGC
TGGCTGGTTTATTGCTGATAAATCTGGAGCCGGTGAGCGTGGAAGCCGCGGTATCAT
TGCAGCACTGGGGCCAGATGGTAAGCCCTCCCGTATCGTAGTTATCTACACGACGGG
GAGTCAGGCAACTATGGATGAACGAAATAGACAGATCGCTGAGATAGGTGCCTCACT
GATTAAGCATTGGTAACTGTCAGACCAAGTTTACTCATATATACTTTAGATTGATTT
AAAACTTCATTTTTAATTTAAAAGGATCTAGGTGAAGATCCTTTTTGATAATCTCAT
GACCAAAATCCCTTAACGTGAGTTTTCGTTCCACTGAGCGTCAGACCCCGTAGAAAA
GATCAAAGGATCTTCTTGAGATCCTTTTTTTCTGCGCGTAATCTGCTGCTTGCAAAC
AAAAAAACCACCGCTACCAGCGGTGGTTTGTTTGCCGGATCAAGAGCTACCAACTCT
TTTTCCGAAGGTAACTGGCTTCAGCAGAGCGCAGATACCAAATACTGTCCTTCTAGT
GTAGCCGTAGTTAGGCCACCACTTCAAGAACTCTGTAGCACCGCCTACATACCTCGC
TCTGCTAATCCTGTTACCAGTGGCTGCTGCCAGTGGCGATAAGTCGTGTCTTACCGG
GTTGGACTCAAGACGATAGTTACCGGATAAGGCGCAGCGGTCGGGCTGAACGGGGGG
TTCGTGCACACAGCCCAGCTTGGAGCGAACGACCTACACCGAACTGAGATACCTACA
GCGTGAGCTATGAGAAAGCGCCACGCTTCCCGAAGGGAGAAAGGCGGACAGGTATCC
GGTAAGCGGCAGGGTCGGAACAGGAGAGCGCACGAGGGAGCTTCCAGGGGGAAACGC
CTGGTATCTTTATAGTCCTGTCGGGTTTCGCCACCTCTGACTTGAGCGTCGATTTTT
GTGATGCTCGTCAGGGGGGCGGAGCCTATGGAAAAACGCCAGCAACGCGGCCTTTTT
ACGGTTCCTGGCCTTTTGCTGGCCTTTTGCTCACATGT

Table S1

Gene	Exon	Orientation	Forward oligo (5' -> 3')	Reverse oligo (5' -> 3')
VEGFA	1	S	accgGAAACTTTTCGTCCAACTTC	aaacGAAGTTGGACGAAAAGTTTC
		AS	accgGACCCCCTCCACCCCGCCTC	aaacGAGGCGGGGTGGAGGGGGTC
MDC1	9	S	accgCAGGATCAGAGCTGCTGAGA	aaacTCTCAGCAGCTCTGATCCTG
		AS	accgCAGGTGGCATCTTGCAATTC	aaacGAATTGCAAGATGCCACCTG
53BP1	10	S	accgGCAGTCCCACAGAGCAAGAA	aaacTTCTTGCTCTGTGGGACTGC
		AS	accgGAACGATAAAAGGAGTAGAT	aaacATCTACTCCTTTTATCGTTC
RIF1	3	S	accgGTGCTGCTCTACAAGCCCTG	aaacCAGGGCTTGTAGAGCAGCAC
		AS	accgACTCAGCTCCGAGTTTTGAC	aaacGTCAAAACTCGGAGCTGAGT
LMNA	2	S	accgTCTCAGTGAGAAGCGCACGC	aaacGCGTGCGCTTCTCACTGAGA
		AS	accgAGTGCTCAGTGCGGCCTCCT	aaacAGGAGGCCGCACTGAGCACT
TP53	6	S	accgAAATTTGCGTGTGGAGTATT	aaacAATACTCCACACGCAAATTT
		AS	accgTCCACTCGGATAAGATGCTG	aaacCAGCATCTTATCCGAGTGGA
H2AFX	1	S	accgTACTAAGAGGGCCCGCGCCG	aaacCGGCGCGGGCCCTCTTAGTA
		AS	accgGTGGCCTTCTTGCCGCCCGA	aaacTCGGGCGGCAAGAAGGCCAC

Supplementary Table 1

DNA oligos used to form duplexes (forward oligo is complementary with reverse oligo) for cloning sense or antisense sgRNA sequences into all-in-one vector plasmids. Lowercase sequences (accg and aaac) are the 5' overhangs for direct ligation with *BsaI*- or *BbsI*-digested all-in-one vectors.

Table S2

Target	Sequence (5' -> 3')	Length (nt)	Amplicon (bp)	Gene_Chromosome
VEGFA_On-target_F	AGAGAAGTCGAGGAAGAGAGAG	22	712 bp	*VEGFA_ch06*
VEGFA_On-target_R	CAGCAGAAAGTTCATGGTTTCG	22		
Off-target_AS-01_F	TGGGTTGAAAACAGTGCGGT	20	351 bp	*FMN1_ch15*
AS-01_R	AACCCTCAGCACCGGTAGAA	20		
AS-02_F	AAAGTTTGCCAACGCTCCTG	20	467 bp	*PAX6_ch11*
AS-02_R	TGCCTAAACTCTGACTGGGC	20		
AS-06_F	AAGGGGCTGCTGGGTAGGAC	20	197 bp	*PAPD7_ch05*
AS-06_R	CGTGATTCGAGTTCCTGGCA	20		
AS-09_F	GAGGTGACAGCAGTGGACAG	20	316 bp	*LAMA3_ch18*
AS-09_R	TGCTTCTTGCTCTGACCTTGTT	22		
AS-15_F	ATCCGGACCAGAAAACTGCC	20	430 bp	*SPNS3_ch17*
AS-15_R	CCTCCCTTCCCGACATCCTA	20		
AS-17_F	CCCATGAGGGGTTTGAGTGC	20	290 bp	*ch09*
AS-17_R	TGAAGATGGGCAGTTTGGGG	20		
AS-19_F	GTCTCCCGAAGTTCTTGAGTCT	22	323 bp	*HDLBP_ch02*
AS-19_R	GCCCACTAGTGTAGAGGGGA	20		
AS-20_F	GCCAGTCCAGGAACTATATC	20	352 bp	*ABLIM1_ch10*
AS-20_R	GGAAGTCAACCAGGATTATTG	21		
AS-23_F	GTCACGTCACAGACTCGCTT	20	870 bp	*CALY_ch10*
AS-23_R	GAGCCGCCTGTGAGCTATT	19		
AS-23_F2	TGCTGCAGGTGGTTCCGGAG	20	637 bp	*CALY_ch10*
AS-23_R2	CTGGAACCGCATCCTCCGCA	20		
AS-24_F	TTCTAGCCCATTGATCCGCC	20	840 bp	*ch04*
AS-24_R	CAGTCTCTGCCCCCAAAGAG	20		
AS-29_F	AGGCAGGTATTTCGGAAGCC	20	913 bp	*ACLY_ch17*
AS-29_R	TATGGCCCAAGGCAAAACCT	20		
AS-34_F	CGGAGCGTGGCTTTATTTGC	20	943 bp	*ch04*
AS-34_R	GCGTGAGGGTCACACTGTAG	20		
MDC1_On-target_F	CCAAAGTGATCCTGGAGAGAGATAC	25	745 bp	*MDC1_ch06*
MDC1_On-target_R	CTCTCCTCCATTAGACTGGGATCTA	25		
MDC1_Off-target_F	GTGCGGAGGGAGTTCCTATG	20	1050 bp	*LAMA3_ch18*
MDC1_Off-target_R	GGGGGTTTCAGGGTCTCAAT	20		
53BP1_On-target_F	TATTTCCTAGCACTGCTCATTTTGC	25	727 bp	*53BP1_ch15*
53BP1_On-target_R	CTGAAGGGCTCCTCAAGTGC	20		

Supplementary Table 2

Primers used for PCRs across on- or off-target sites in T7 EI assays.

Table S3

Gene	Orientation	Sequence (5' -> 3')	Length (nt)	Amplicon (bp)	Purpose
MDC1	F	CTGCTTGGAACTCAGCCACC	20	164 bp	Genotypic screening
	R	GAAGATATAGAGATGACTTGTGGAATAGGAGG	32		
	F	CCAAAGTGATCCTGGAGAGAGATAC	25	745 bp	DNA sequencing
	R	CTCTCCTCCATTAGACTGGGATCTA	25		
53BP1	F	AAGGAATTCTTCAGATCTTGTTGC	24	162 bp	Genotypic screening
	R	CAAGGCAGAAAAAGTGTTGCTC	22		
	F	TATTTCCTAGCACTGCTCATTTTGC	25	727 bp	DNA sequencing
	R	CTGAAGGGCTCCTCAAGTGC	20		
RIF1	F1	GCTTGTTCTGTAATCAACTTCTGC	24	182 bp	Genotypic screening
	R1	CCTGATAATTCTGAGGTAATTTTGGG	26		
	F2	ATTGATTTACAGTTGTGCTTGTTTG	25	244 bp	Genotypic screening
	R2	CCAGACATAACAGTACTTTTAACAGCATC	29		
	F	TTCATCTGTTGACCTCTTTGCTTTC	25	791 bp	DNA sequencing
	R	GTCTCACTTATCACCCAGGTTAGAG	25		
LMNA	F	GCAATACCAAGAAGGAGGGTG	21	207 bp	Genotypic screening
	R	GTACATGTGTTAGGTGGGGCC	21		
	F	CAGGCACGTTCCTGAGCC	18	702 bp	DNA sequencing
	R	TCGATCTCCTGACCTCGTGAT	21		
TP53	F	GAGACGACAGGGCTGGTTG	19	221 bp	Genotypic screening
	R	ACCACCCTTAACCCCTCCTC	20		
	F	GCCAACTCTCTCTAGCTCGC	20	703 bp	DNA sequencing
	R	AGGCCCTTAGCCTCTGTAAGC	21		
H2AFX	F	TACCTCACCGCTGAGATCCTG	21	363 bp	Genotypic screening
	R	TCTGAAGCGGCTCAGCTCTTT	21		
	F	AGTGGCCCAGTCACATGAAG	20	4571 bp	DNA sequencing/Long PCR
	R	CCAGGTACCAAAGCTGGAGG	20		

Supplementary Table 3

PCR-primers used for the purposes of genotypic screening or DNA sequencing.

Table S4

Target	Host	Supplier	Reference	Dilution factor (IF)	Dilution factor (IB)
RIF1	Rabbit	Bethyl Laboratories	A300-569A	1/500	1/1000
MDC1	Rabbit	Abcam	ab11171	1/500	1/5000
53BP1	Rabbit	Novus Biologicals	NB100-304	1/500	1/1000
53BP1	Mouse	A gift from J. Chen	University of Texas (USA)	1/100	-
Lamin A/C	Goat	Santa Cruz Biotechnology	sc-6215	1/100	1/200
α-tubulin	Mouse	Sigma-Aldrich	T9026	-	1/5000
P53	Mouse	Santa Cruz Biotechnology	DO1/sc-126	-	1/1000
$P53^{pS15}$	Rabbit	Cell Signaling Technology	9284L	-	1/1000
KAP1	Rabbit	Abcam	ab10483	-	1/1000
$KAP1^{pS824}$	Rabbit	Bethyl Laboratories	IHC-00073	-	1/1000
P21(CIP1)	Rabbit	Santa Cruz Biotechnology	sc-397	-	1/1000
H2AX	Rabbit	Abcam	ab11175	-	1/2000
$H2AX^{pS139}$	Mouse	Millipore	05-636	1/500	1/2000

Supplementary Table 4

Primary antibodies used for immunofluorescence (IF) staining or immunoblotting (IB) in this study. All secondary (Alexa Fluor 488/594) antibodies were purchased from ThermoFisher and used in 1/1000 dilution with appropriate host species against target species.
